# Chinese and Global Burdens of Gastrointestinal Cancers From 1990 to 2019

**DOI:** 10.3389/fpubh.2022.941284

**Published:** 2022-07-13

**Authors:** Wangcheng Xie, Tingsong Yang, Jieliang Zuo, Zhilong Ma, Weidi Yu, Zhengyu Hu, Zhenshun Song

**Affiliations:** ^1^Department of General Surgery, Shanghai Tenth People's Hospital, Tongji University School of Medicine, Shanghai, China; ^2^Department of General Surgery, Tongren Hospital, Shanghai Jiao Tong University School of Medicine, Shanghai, China; ^3^Department of General Surgery, Shanghai Fourth People's Hospital, Tongji University School of Medicine, Shanghai, China

**Keywords:** death, disability-adjusted life-years, gastrointestinal cancer, incidence, risk factor

## Abstract

**Background:**

Gastrointestinal (GI) cancers are an important component of the tumor. This study aimed to investigate the burden of six major GI cancers in China and globally from 1990 to 2019.

**Methods:**

We conducted a cross-sectional study based on the Global Burden of Disease Study (GBD) 2019. Indicators on incidence, deaths, disability-adjusted life-years (DALYs), and risk factors for esophageal, stomach, liver, pancreatic, colon and rectum, and gallbladder and biliary tract cancers were collected and analyzed for time trends. The contribution of each cancer and the proportion of cases in China among global cases were further reported.

**Results:**

Global incidence cases, death cases, and DALYs of GI cancers showed an overall ascending trend over the past 30 years, but there was temporal and geographical variation across cancer types. By 2019, colon and rectum cancer had overtaken stomach cancer as the most burdensome GI cancer globally. However, stomach cancer narrowly continued to be the most burdensome GI in China. In addition, the proportion of incidence and death cases of stomach, pancreatic, colon and rectum, and gallbladder and biliary tract cancers among global cases had further increased. It was noteworthy that the burden of liver cancer in China has been alleviated significantly.

**Conclusion:**

GI cancers remain a major public health problem in China and globally. Despite the temporal and geographic diversity of different cancers, targeted primary and secondary prevention are still necessary for the future to face these unknown challenges.

## Introduction

Gastrointestinal (GI) cancers mainly include esophageal, stomach, liver, pancreatic, colon and rectum, and gallbladder and biliary tract cancers. According to GLOBOCAN 2020, there would be an estimated 19.3 million new cancer cases and 10 million deaths worldwide in 2020, with GI cancers accounting for 26.4% of incidences and a serious 36.3% of deaths (1). Furthermore, multiple GI cancers are in the top 10 in incidence and mortality, constituting a serious burden for many countries (1).

The incidence and mortality of GI cancers also vary between countries and regions. Asia suffers from the largest number of cases of incidence and deaths, with esophageal, stomach, and liver cancers being the most common (2). As the country with the largest population, the new cases in China greatly exceed others in the world, showing a heavy burden of GI cancers (1). Moreover, China is experiencing an unprecedented population aging, which has the potential to pose a huge challenge to the prevention of GI cancers (3). However, a recent study pointed out that the burden of liver, stomach, and esophageal cancers would diminish in China in 2022 and the future trend of GI cancers would be similar to that of America (4).

Anyway, it is beneficial to identify patterns and trends in incidence and mortality for alleviation of the GI cancer burden. Therefore, the cross-sectional research provided a comprehensive description of the burden of six major GI cancers in China and globally from 1990 to 2019. Although some indicators for certain cancers have been reported, our findings not only serve as a complement to previous results but also provide help for decision-makers to evaluate the total burden of GI cancers for formulating targeting prevention strategies and allocating public health resources (5, 6).

## Methods

### Data Collection

All data were obtained from the Global Burden of Disease Study (GBD) 2019 by utilizing the Global Health Data Exchange (GHDx) query tool (http://ghdx.healthdata.org/gbd-results-tool) (7, 8). Indicators of incidence, deaths, disability-adjusted life-years (DALYs), and risk factors were collected between 1990 and 2019 by sex, country, and region for six GI cancers, including esophageal, stomach, liver, pancreatic, colon and rectum, and gallbladder and biliary tract cancers. The 204 countries and territories are classified into five groups based on the socio-demographic index (SDI) ranging from high, high-middle, middle, low-middle, and low. In addition, they were subdivided into 21 regions in terms of geographical location.

This study was based on a publicly available database and exempted by the Ethics Committee of Shanghai Tenth People's Hospital.

### Statistical Analysis

First, we analyzed the burden of GI cancers at the national level in China and globally. To offset demographic differences, age-standardized incidence rates (ASIR), death rates, and DALY rates were used to better reflect actual incidence and mortality profiles. All rates are expressed as numbers per 100,000 population. In addition, an estimated annual percentage change (EAPC) was calculated *via* a linear regression equation: y = α + βx + ε (y represents ln (ASIR) and x refers to calendar year), to evaluate the trend in ASIR. The exact calculation was EAPC = 100^*^(exp(β)-1) (9). Both the EAPC and its 95% confidence interval (CI) above 0 indicated an increasing trend in ASIR over the years, while both below 0 implied a decreasing trend, otherwise, it illustrated a stabilizing ASIR (10).

Next, we explored risk factors for GI cancers and analyzed their evolution over the last 30 years in China and globally. The risk factors included body mass index (BMI), smoking, alcohol use, and diet.

In addition, we performed clustering of 204 countries and territories by combining the EAPC for each cancer incidence and death rate. Finally, we analyzed the changes in the contribution of the six GI cancers in 1990 and 2019, as well as the changes in the proportion of incidence and death cases in China among global cases.

All statistical analysis was performed with R software (version 4.0.5).

## Results

### Esophageal Cancer

**Figure 1:**
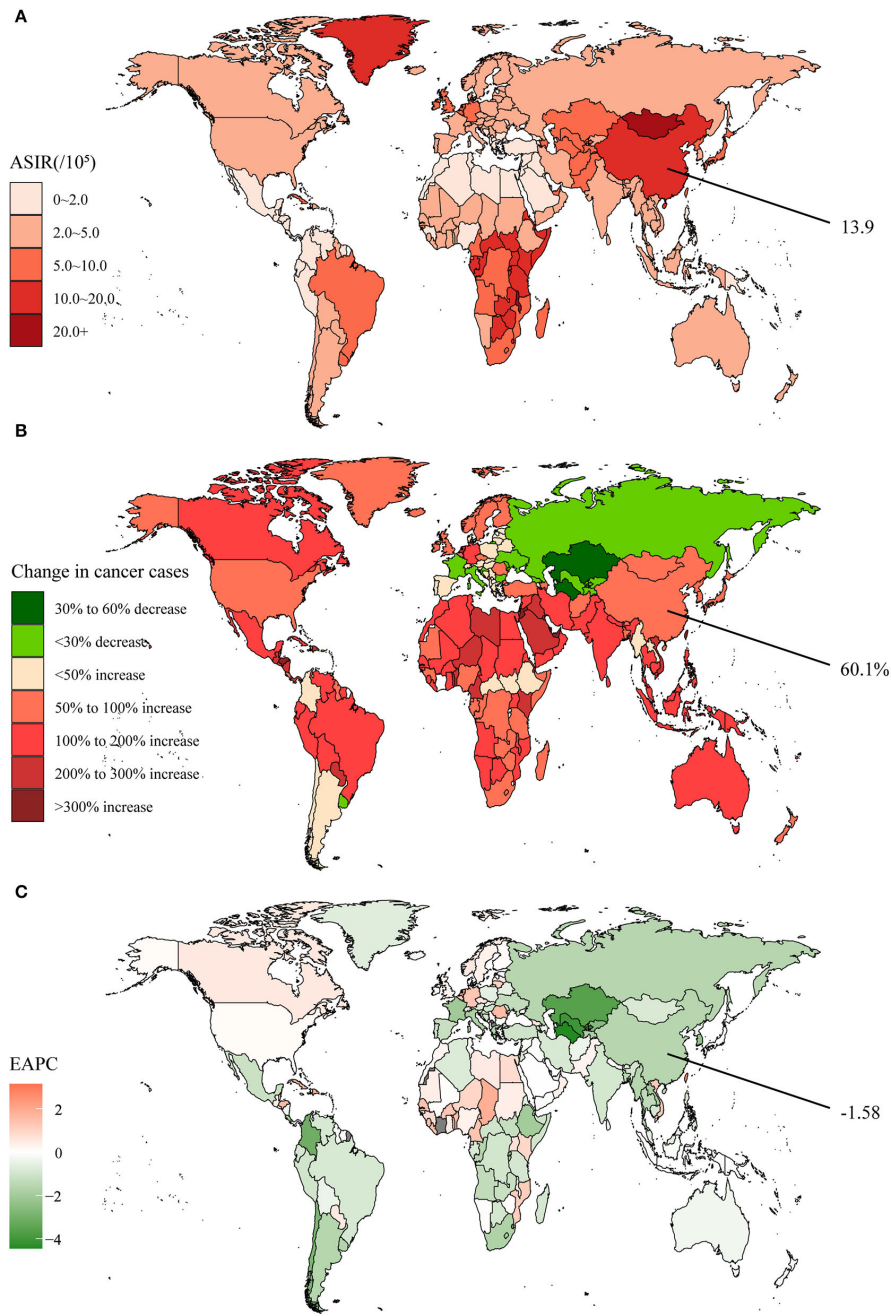
The global disease burden of esophageal cancer in 204 countries and territories. **(A)** The ASIR in 2019; **(B)** The relative change in incident cases between 1990 and 2019; **(C)** The EAPC of ASIR from 1990 to 2019. Outcomes of China were specifically annotated. ASIR, age-standardized incidence rate; EAPC, estimated annual percentage change.

Over the past 30 years, despite decreases in a few countries such as Russia and Kazakhstan, the global incidence cases of esophageal cancer had increased from 320.0 × 10^3^ to 534.6 × 10^3^ with an average increase of 67.1% (Supplementary Table S1, Figure 1B). In 2019, the ASIR varied considerably around the world, being the highest in Malawi (Figure 1A). But overall, since global EAPC was−0.90, ASIR was declining annually in most countries (Supplementary Table S1, Figure 1C). The incidence cases of esophageal cancer in China were only 173.7 × 10^3^ in 1990 and reached 278.1 × 10^3^ in 2019, with an increase of 60.1% (Supplementary Table S1). The ASIR was 13.9 per 100,000 population and had an above-average decline over 30 years (EAPC = −1.58).

In addition, after a temporary reduction, death cases and DALYs restarted a slight increase in 2014, both globally and in China (Supplementary Figures S1A,B). However, since the peak in 2004, the age-standardized death and DALY rates had decreased year by year and leveled off in recent years. It was evident that the death and DALY rates were still higher in China compared to the global average in 2019, with a margin of 7.0 and 137.7 per 100,000 population, respectively. By clustering, we found that the integrated EAPC of incidence and deaths were relatively similar among China and other 38 countries and territories such as Greece and Italy (Supplementary Figure S2).

Globally, most deaths and DALYs could be attributed to the following six risk factors, including smoking, alcohol use, high BMI, diet low in fruit, diet low in vegetables, and chewing tobacco. The impact of these risk factors varied by region and time, for instance, chewing tobacco mainly affected South Asia (Supplementary Figures S3A,B). In addition, the contribution of high BMI had surpassed diet low in fruit in 2019, with global attribution rates approaching 20%. The major risk factors for death and DALYs currently in China were smoking (47.8% and 48.6%), alcohol use (24.0% and 26.2%), and high BMI (14.1% and 14.9%).

### Stomach Cancer

**Figure 2:**
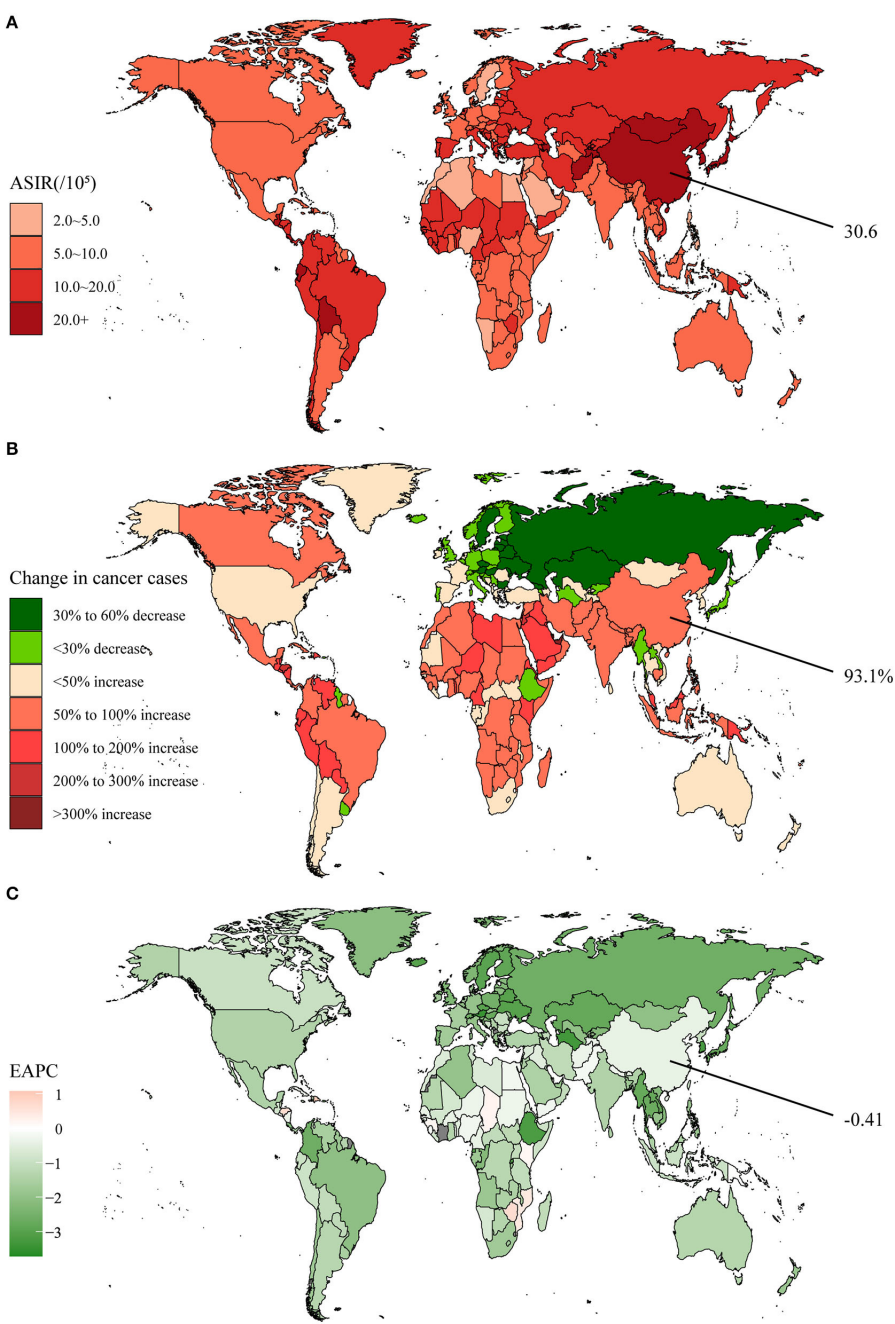
The global disease burden of stomach cancer in 204 countries and territories. **(A)** The ASIR in 2019; **(B)** The relative change in incident cases between 1990 and 2019; **(C)** The EAPC of ASIR from 1990 to 2019. Outcomes of China were specifically annotated. ASIR, age-standardized incidence rate; EAPC, estimated annual percentage change.

Stomach cancer, an important component of GI cancers, has been characterized by high incidence and mortality. Between 1990 and 2019, the global incidence cases of stomach cancer increased from 883.4 × 10^3^ to 1,269.8 × 10^3^, representing an increase of 43.7% (Supplementary Table S2). However, several countries in Europe experienced negative growth in incidence in recent years (Figure 2B). The global stomach cancer ASIR was 15.6 per 100,000 population in 2019 (Supplementary Table S2, Figure 2A). With an average EAPC of −1.22, the ASIR dropped yearly in almost all the countries (Supplementary Table S2, Figure 2C). The incidence cases in China were 317.3 × 10^3^ in 1990 and had grown to 612.8 × 10^3^ in 2019, nearly doubling in 30 years (Supplementary Table S2). The ASIR was 30.6 per 100,000 population in 2019, ranking third globally, and the EAPC was only −0.41. It was notable that the ASIR for Chinese males remained stable in recent years, without a downward trend.

In 2019, the global death cases of stomach cancer reached a new record of 957.2 × 10^3^. Even though peaking in 2005, it still amounted to 421.5 × 10^3^ in 2019 in China (Supplementary Figure S4A). DALYs showed a steady trend in China during the past decade, in contrast to the global re-emergence of slow growth (Supplementary Figure S4B). Nevertheless, the age-standardized death and DALY rates had been decreasing both globally and in China (Supplementary Figures S4A,B). Even though there was still a distinct gap with the average, the decrease rate was proved to be faster in China. By clustering, we found that the integrated EAPC of incidence and deaths were relatively similar among China and other 110 countries and territories such as Lebanon, France, and the United States of America (Supplementary Figure S5).

GBD 2019 tallied two risk factors, smoking and a diet high in sodium, for stomach cancer deaths and DALYs. Smoking influenced more countries than a diet high in sodium, even though the attributed rate decreased by ~1% in 2019, which was mainly in the high SDI region (Supplementary Figures S6A,B). Conversely, the attributed rate of smoking increased by around 3% in China over 30 years.

### Liver Cancer

**Figure 3:**
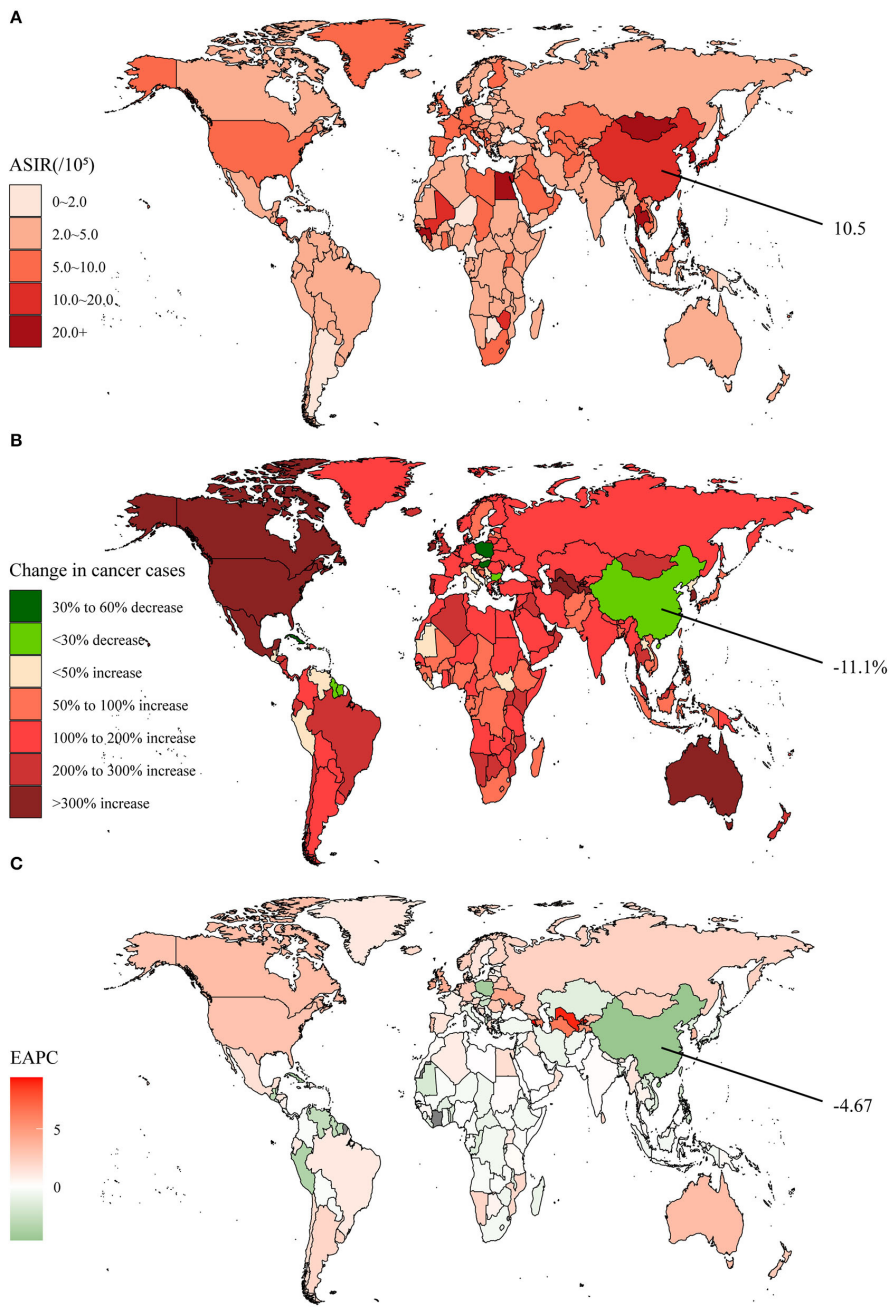
The global disease burden of liver cancer in 204 countries and territories. **(A)** The ASIR in 2019; **(B)** The relative change in incident cases between 1990 and 2019; **(C)** The EAPC of ASIR from 1990 to 2019. Outcomes of China were specifically annotated. ASIR, age-standardized incidence rate; EAPC, estimated annual percentage change.

Liver cancer was the 7th most commonly diagnosed cancer, with 534.4 × 10^3^ new cases worldwide in 2019, increasing by 43.1% from 1990 (Supplementary Table S3). Developed countries such as the United States of America, Canada, and Australia elevated even more than 3-fold (Figure 3B). The 2019 global ASIR was 6.5 per 100,000 population, and the highest observed remained in Mongolia at 105.2 per 100,000 population (Supplementary Table S3, Figure 3A). The ASIR declined annually in all regions except for the high SDI regions, as the global average EAPC was −1.93 (Supplementary Table S3, Figure 3C). The incidence cases of liver cancer reduced from 236.8 × 10^3^ in 1990 to 210.5 × 10^3^ in 2019 with a decline of 11.1% in China, one of the few countries worldwide experiencing a decline (Supplementary Table S3). Despite a relatively high level of 10.5 per 100,000 population in 2019, the ASIR declined much faster than the global level (EAPC = −4.67), ranking second.

After experiencing a temporary downslope in the early 20th century, the global and Chinese liver cancer deaths and DALYs reappeared in a fluctuating upward trend (Supplementary Figures S7A,B). However, the death cases and DALYs were still well below the peaks until 2019 in China, at 187.7 × 10^3^ and 5,325.5 × 10^3^, respectively. It was apparent that the global age-standardized death and DALY rates decreased significantly around 2,000, especially in China, and were maintained relatively stable in recent years. By clustering, we found that the integrated EAPC of incidence and deaths were relatively similar among China and other 51 countries and territories such as Poland and Peru (Supplementary Figure S8).

In exclusion of viral infections, GBD 2019 categorized the risk factors for liver cancer deaths and DALYs into five groups, as follows: smoking, alcohol use, high BMI, drug use, and high fasting plasma glucose. Compared to smoking, the attributed rate of alcohol use, high BMI, and drug use were all moderately upward in 2019 (Supplementary Figures S9A,B). Conversely, the rates of smoking-influenced deaths and DALYs in China were 20.0 and 19.1% respectively, both higher than in 1990. In addition, high BMI had been a rapidly developing and important risk factor in recent years (10.1% of deaths and 10.7% of DALYs).

### Pancreatic Cancer

**Figure 4:**
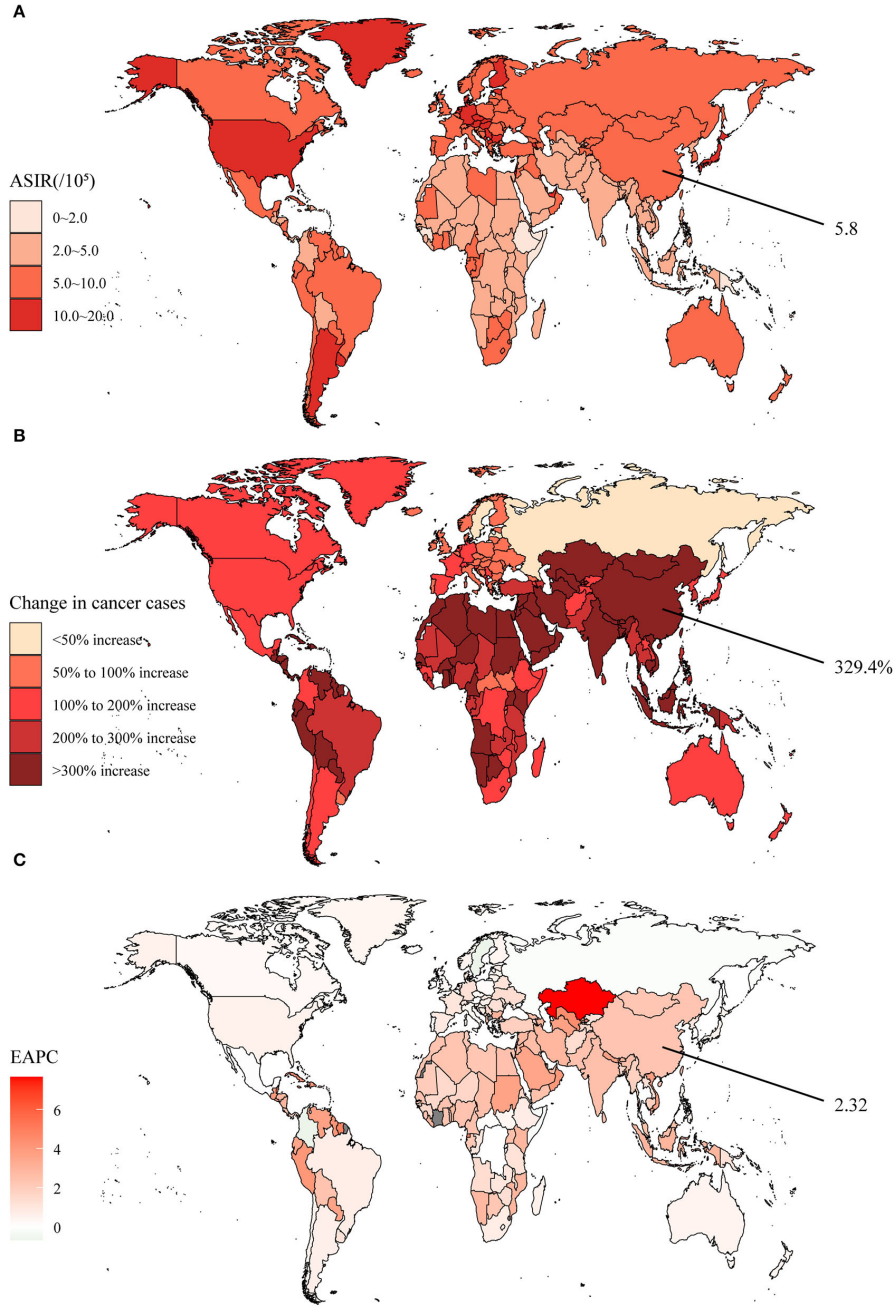
The global disease burden of pancreatic cancer in 204 countries and territories. **(A)** The ASIR in 2019; **(B)** The relative change in incident cases between 1990 and 2019; **(C)** The EAPC of ASIR from 1990 to 2019. Outcomes of China were specifically annotated. ASIR, age-standardized incidence rate; EAPC, estimated annual percentage change.

The pancreas is unique in being both an endocrine and an exocrine gland and serves as an important component of the gastrointestinal tract. Between 1990 and 2019, the global incidence cases of pancreatic cancer evolved from 197.3 × 10^3^ to 530.3 × 10^3^, and the new cases in both sexes were similar (Supplementary Table S4). The global increase was 168.7%, with several countries in Asia, Africa, and Latin America even increasing more than 3-fold (Supplementary Table S4, Figure 4B). In 2019, the ASIR was 6.6 per 100,000 population, as the higher regions continued to be Europe and North America (Supplementary Table S4, Figure 4A). In general, the ASIR was escalating year by year in almost all countries, with a global EAPC of 0.83, among which Kazakhstan even reached 7.66 (Supplementary Table S4, Figure 4C). In China, the number of pancreatic cancer incidence cases had climbed by 329.4% over 30 years, developing from 26.8 × 10^3^ to 115.0 × 10^3^ (Supplementary Table S4). The 2019 ASIR of 5.8 per 100,000 population was slightly below the global level, but the rise speed in ASIR was much quicker (EAPC = 2.32).

Both globally and in China, the death cases and DALYs were escalating over the years (Supplementary Figures S10A,B). In 2019, the number of death cases caused by pancreatic cancer was 531.1 × 10^3^ globally, of which 117.4 × 10^3^ occurred in China. In addition, age-standardized death rates and DALY rates also showed an increasing trend, and the rate of increase in China was significantly ahead of worldwide. By clustering, we found that the integrated EAPC of incidence and deaths were relatively similar among China and other 72 countries and territories such as Mongolia and Togo (Supplementary Figure S11).

Pancreatic cancer deaths and DALYs were predominantly attributed to smoking, high BMI, and high fasting plasma glucose. While smoking persisted as the main risk factor in most regions globally, the rate attributable to high BMI and high fasting plasma glucose also increased in 2019 (Supplementary Figures S12A,B). In China, the current proportions of deaths and DALYs attributed to these three exposures were 22.6% and 21.8% for smoking, 6.9% and 6.3% for high fasting plasma glucose, and 3.6% and 3.8% for high BMI.

### Colon and Rectum Cancer

**Figure 5:**
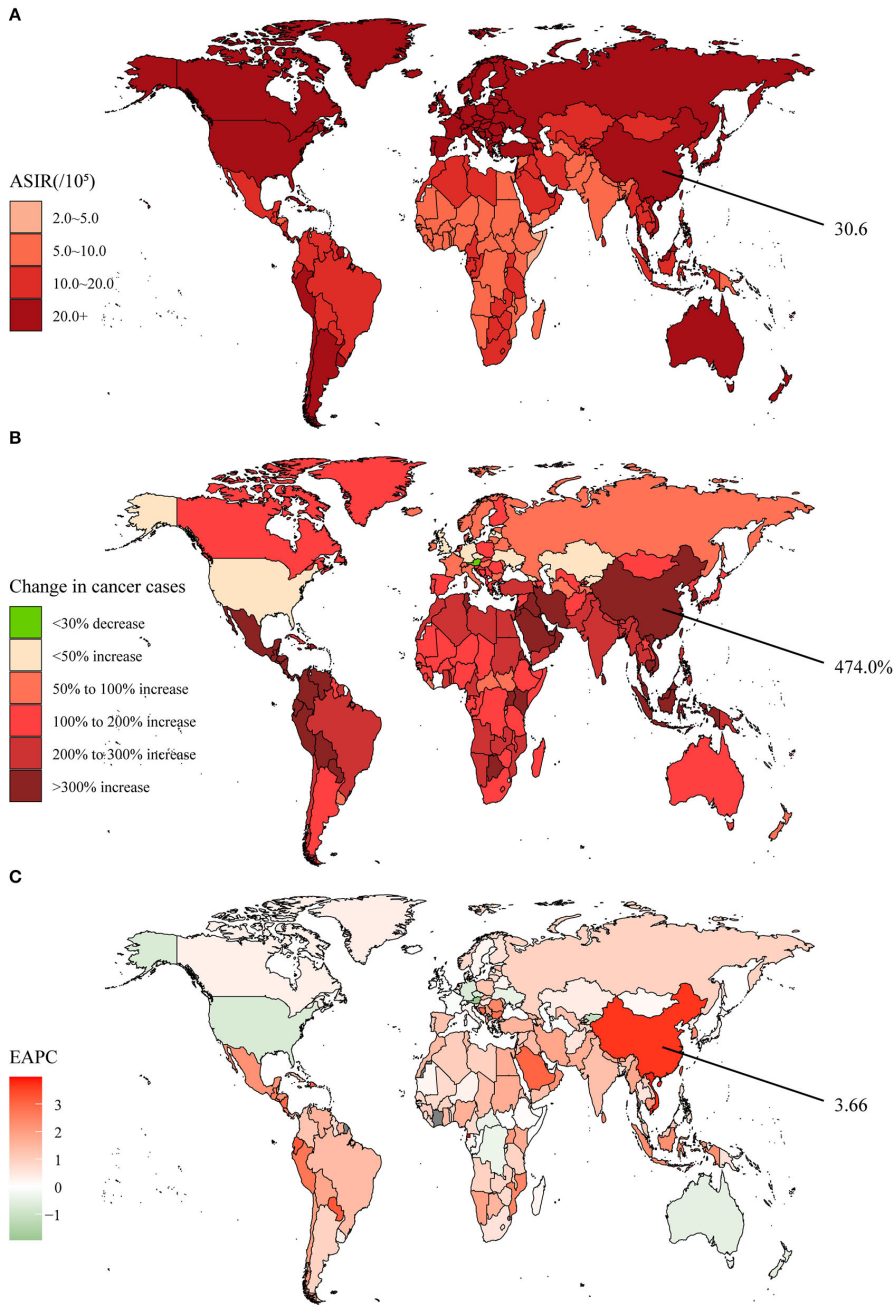
The global disease burden of colon and rectum cancer in 204 countries and territories. **(A)** The ASIR in 2019; **(B)** The relative change in incident cases between 1990 and 2019; **(C)** The EAPC of ASIR from 1990 to 2019. Outcomes of China were specifically annotated. ASIR, age-standardized incidence rate; EAPC, estimated annual percentage change.

Colon and rectum cancer has become the most common GI cancer. Between 1990 and 2019, the global new cases increased by 157.2%, developing from 842.1 × 10^3^ to 2,166.2 × 10^3^ (Supplementary Table S5). Surprisingly, Austria became the only country with negative growth, albeit by only 0.6% (Figure 5B). The global ASIR achieved 26.7 per 100,000 population in 2019, with the highest being Taiwan (Province of China) at 62.0 per 100,000 population (Supplementary Table S5, Figure 5A). The EAPC of 0.58 indicated a yearly increment in the global ASIR, while the high SDI regions showed a decreasing tendency (Supplementary Table S5, Figure 5C). Chinese new cases amounted to 607.9 × 10^3^ in 2019, representing a growth of 474.0% (Supplementary Table S5). The ASIR was 30.6 per 100,000 population and the male ASIR was nearly twice as high as the female. The EAPC was much higher than the global standard over 30 years, reaching a level of 3.66.

Both global and Chinese deaths and DALY cases had been increasing for 30 years (Supplementary Figures S13A,B). In addition, global age-standardized death rates and DALY rates showed a downward trend. However, overall China represented an increase, although there was a short-lived decrease in the last 10 years. By clustering, we found that the integrated EAPC of incidence and deaths were relatively similar among China and other 19 countries and territories such as Lebanon and Saudi Arabia (Supplementary Figure S14).

There were numerous risk factors associated with colon and rectum cancer deaths and DALYs, and GBD 2019 categorized the following eight, including smoking, alcohol use, high BMI, high fasting plasma glucose, diet low in fiber, diet low in whole grains, diet high in red meat, and diet high in processed meat. Compared with 1990, the risk factors attribution ratio did not change significantly (Supplementary Figures S15A,B). For China, the two main influencing factors were smoking (15.9% of deaths and 15.4% of DALYs) and diet low in whole grains (15.9% of deaths and 15.9% of DALYs).

### Gallbladder and Biliary Tract Cancer

**Figure 6:**
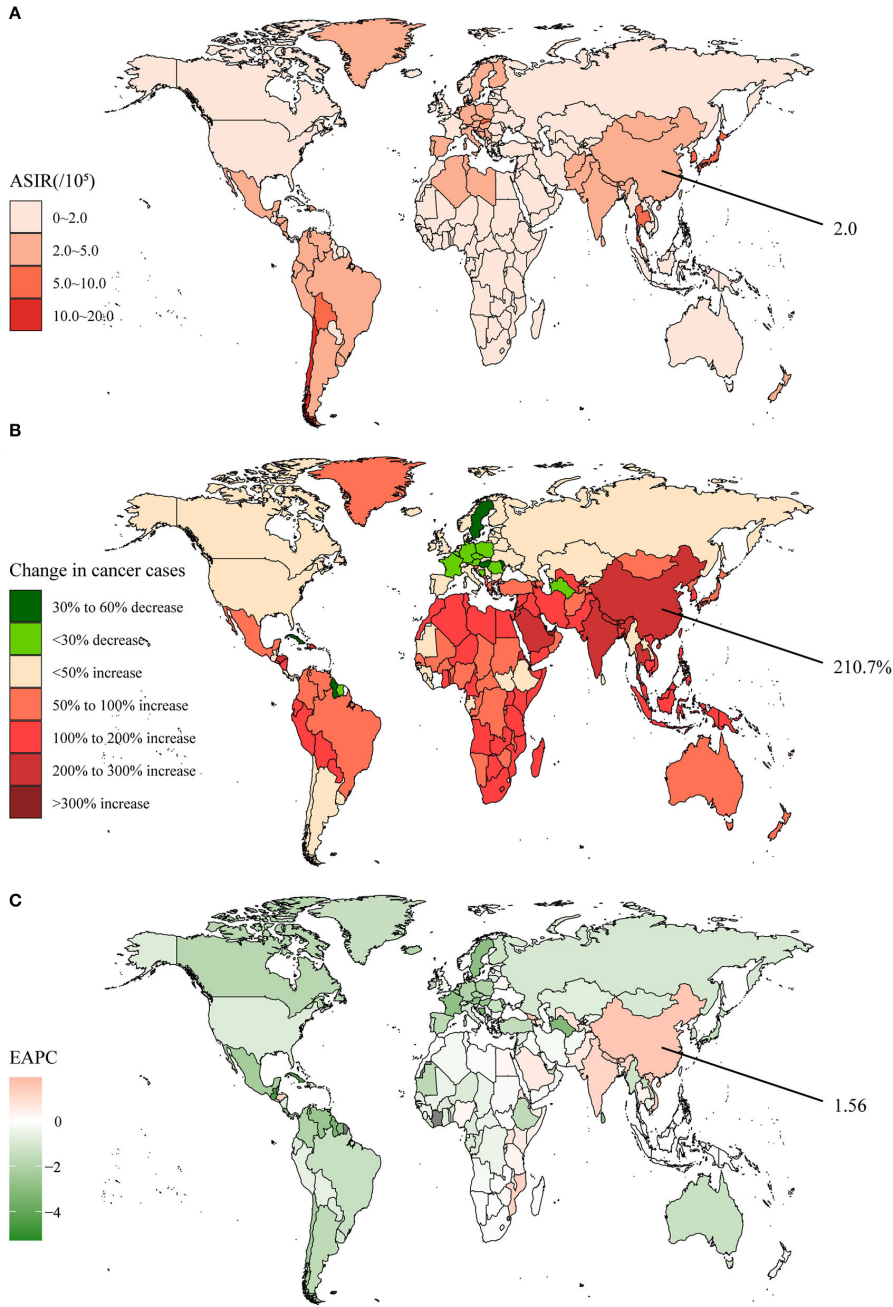
The global disease burden of the gallbladder and biliary tract cancer in 204 countries and territories. **(A)** The ASIR in 2019; **(B)** The relative change in incident cases between 1990 and 2019; **(C)** The EAPC of ASIR from 1990 to 2019. Outcomes of China were specifically annotated. ASIR, age-standardized incidence rate; EAPC, estimated annual percentage change.

The incidence of the gallbladder and biliary tract cancer was the lowest compared to the other five types of GI cancer. In 2019, the global incidence cases of the gallbladder and biliary tract cancer was 199.2 × 10^3^, and it was the only type of cancer with more female than male patients (Supplementary Table S6). In addition, the faster-growing countries were mostly in Asia and Africa, while many countries in Europe showed negative growth (Figure 6B). The global ASIR was 2.5 per 100,000 population, with Chile being the highest (11.1 per 100,000 population) (Supplementary Table S6, Figure 6A). In the 30 years, the ASIR has dropped in more than half of the countries worldwide, with an average EAPC of −0.48 (Supplementary Table S6, Figure 6C). The Chinese incidence cases of the gallbladder and biliary tract cancer were merely 12.4 × 10^3^ in 1990, but reached 38.6 × 10^3^ in 2019 and had a growth rate of 210.7%, ranking 13th in the world (Supplementary Table S6). The 2019 ASIR was 2.0 per 100,000 population, which was lower than the global level, but displayed an elevated tendency over 30 years (EAPC = 1.56).

Despite a continuous increase in death cases and DALYs, global age-standardized death and DALY rates appeared on the decline (Supplementary Figures S16A,B). While there was a rapid rise in Chinese age-standardized death and DALY rates around the year 2000, a downward trend returned in the last decade. By clustering, we found that the integrated EAPC of incidence and deaths were relatively similar among China and other 73 countries and territories such as Vietnam and India (Supplementary Figure S17).

For risk factors for gallbladder and bile duct cancer deaths and DALYs, GBD 2019 only summarized high BMI. As could be seen, a higher rate was attributed to high BMI in 2019 (Supplementary Figures S18A,B). In China, 10.2% of deaths and 10.7% of DALYs became attributable to high BMI.

### Contribution of Six GI Cancers

In terms of incidence, the most significant change in the contribution of GI cancers globally over the past 30 years was the decrease in the proportion of stomach cancer and the increase in the proportion of colon and rectum cancer, which had become the most common GI cancer (Supplementary Figure S19A). The situation mentioned above was most typical in the high-middle and middle SDI regions. However, the proportion of esophageal cancer incidence remained considerably high in some low SDI regions, such as Central, Southern, and Eastern Sub-Saharan Africa. In China, although the contribution of colon and rectum cancer increased, stomach cancer did not decline significantly and was still the most common GI cancer. Instead, liver cancer diminished from 27.1 to 11.3%.

Globally, deaths showed a similar pattern to incidence, with colon and rectum cancer replacing stomach cancer as the largest burden of deaths (Supplementary Figure S19B). Even in the high SDI region, pancreatic cancer also overtook stomach cancer as the leading cause of death, reaching 21.0%. But actually, stomach cancer remained the deadliest cancer in the middle SDI, low-middle SDI, and low SDI regions. In China, deaths associated with liver cancer declined, and the three most burdensome cancers were stomach cancer (32.9%), colon and rectum cancer (20.4%), and esophageal cancer (20.1%) at present.

Further, we described the proportion of incidence and death cases in China among global cases for each GI cancer (Supplementary Figures S20A,B). Compared to 1990, the Chinese percentages of liver cancer incidence and deaths in 2019 were both significantly declined, from 63.4 to 39.4% and 63.7 to 38.7%, respectively. Esophageal cancer also showed a decreasing trend. In contrast, the rates of stomach, pancreatic, colon and rectum, and gallbladder and biliary tract cancers all appeared to be elevated to varying degrees.

## Discussion

In this study, we reported incidence, deaths, DALYs, and risk factors for six major gastrointestinal cancers including esophageal, stomach, liver, pancreatic, colon and rectum, and gallbladder and biliary tract cancers. On a global scale, the total burden of GI cancers had been increasing from 1990 to 2019, while the epidemiology of each cancer varied.

Over the past 30 years, ASIR for esophageal cancer declined globally which might be associated with SDI (11). East Asia and Southern Sub-Saharan Africa remained high-risk regions with high levels of ASIR, as previously reported in the literature (1). A similar decline was not observed in high SDI regions, and this might be due to changes in the incidence pattern of esophageal cancer subtypes (12). In general, esophageal squamous cell carcinoma (ESCC) accounts for 90% of cases and is predicted to a continued decrease in most countries in the future, but the burden of esophageal adenocarcinoma (EAC) will increase dramatically in high-income countries and may exceed the incidence of ESCC in the coming years (13, 14). Smoking and alcohol consumption are recognized as traditional risk factors for esophageal cancer and could even impact disease progression in the genetic level (15). The consequences attributed to high BMI were escalating in recent years. Obesity is a widespread health problem, especially in western countries, and is strongly associated with the progression of esophageal cancer (16). Distinctively, the severe burden of chewing tobacco and a diet low in fruits in South Asia denote the necessity for decision-makers to manage the different regions in accordance with their specificities.

Despite the global decline in incidence and death rates, stomach cancer remains the sixth most common cancer and the third cause of cancer death (1). The decrease in new cases was only observed in European countries, which was probably attributed to the changing age structure and the growing population (5). Recently, a novel view has been proposed that ASIR would fall below the rare disease threshold in multiple countries, indicating that stomach cancer potentially becomes a rare disease in the future (17). In fact, high-risk regions such as Korea and Japan have adopted national screening for better prevention (18, 19). Early diagnosis and treatment, while temporarily increasing the incidence rate, bring long-term benefits in the form of improved 5-year survival rates (20). GBD 2019 did not investigate *H. pylori*, a risk factor affecting 89% of stomach cancer cases, which once treated and eradicated could substantially reduce the incidence and mortality of stomach cancer (21, 22). The high prevalence of *H. pylori* infection in Asia, South America, and Eastern Europe is similar to the geographical distribution of stomach cancer in our findings which deserves attention (23).

We discovered that ASIR for liver cancer in high SDI regions was increasing annually, and this might be strongly related to HBV infection (6). While East and Southeast Asia, a traditionally high-risk region for liver cancer, still maintained a high ASIR, the ASIR has been decreasing year by year in recent years, mainly benefiting from various preventive measures for HBV (24). As can be seen from the results, alcohol consumption was also a major risk factor for liver cancer. The high consumption of alcohol in Europe and North America also contributes to the high mortality in both regions (25). In addition, one of the reasons for the significant difference in incidence between the sexes might also be due to the unhealthy lifestyle of alcohol consumption. In recent years, non-alcoholic fatty liver disease (NASH) has been recognized as an important cause of liver cancer, which may result from obesity, dyslipidemia, and metabolic syndrome (26). However, the insidious onset and challenging early diagnosis of NASH has led to an escalating rate of patients developing liver cancer, particularly in high-income countries (27, 28). The risk factors that predispose to liver cancer are numerous and geographically diverse, making the management control difficult. For most countries, prevention of viral hepatitis should remain the mainstay at this stage.

Pancreatic cancer ASIR was ascending almost globally, the highest in the high SDI region. In addition, since the prognosis is extremely poor, with a 5-year survival rate of only 15 to 25%, pancreatic cancer death and DALY rates are rapidly increasing (29). Its early attack is very covert, and there is no effective approach to early screening today. Therefore, precautions against risk factors become extremely important, especially in individuals with a family history or susceptibility to genetic variants. Studies have stated that smoking, alcohol consumption, high BMI, pancreatitis, and diabetes are all likely to increase the risk of pancreatic cancer (29, 30). As a recognized risk factor, smoking had been attributed a high death and DALY rates in patients for 30 years. We also found that the high fasting plasma glucose attribution ratio increased. In the last few decades, the number of patients with diabetes has expanded to 463 million worldwide (31). A study pointed out that 0.85% of diabetic patients would probably evolve to pancreatic cancer within 3 years (32). This opinion goes some way to explaining the differences in burden across regions. No matter what, a further strengthening of the prevention and treatment of diabetics should be implemented in the future as living standards improve.

Around the world, new cases of colon and rectum cancer had doubled between 1990 and 2019 and had already replaced stomach cancer as the most common GI cancer in terms of incidence and mortality. However, ASIR declined in high SDI regions, among them Austria was the only country where the number of cases decreased, which was facilitated by the longstanding program of screening colonoscopy and fecal tests (33). Through the detection and removal of malignant tumors at an early stage, death rates were not raised markedly. Unhealthy diet, smoking, alcohol consumption, high BMI, and high fasting plasma glucose have always influenced the incidence and prognosis of colon and rectum cancer, and this is also reflected in the fact that the incidence of males has risen faster than that of females in recent years (34, 35). However, it should be emphasized that an important attributing factor for females might be a dietary risk, for which managers are advised to take the distinction into account (36). Nevertheless, the key strategies to address the challenge in the future should be lifestyle changes and early screening of high-risk populations.

Although relatively rare, gallbladder and biliary tract cancer has received increasing attention in recent years due to its high malignancy and poor prognosis. Previous studies had demonstrated that cholelithiasis was the main risk factor and others included chronic inflammation, polyps, obesity, and infection (37). In many high-income countries, laparoscopic and endoscopic techniques have become the first-line management option for cholelithiasis, avoiding malignant transformation (38). At the same time, early operation enables the early identification of cancerous lesions, which in turn reduces mortality. So, poverty, high incidence of cholelithiasis, and poor medical technology are perhaps important reasons for the yearly growth of ASIR in the medium, medium-low, and low SDI regions (39). Moreover, elevated BMI has increased cancer deaths and DALYs in recent years, and based on the dramatic expansion of the obese population, it is essential to further screen for high-risk groups within this population. Interestingly, gallbladder and biliary duct cancer is the only type with a higher incidence in women than in men, which is likely to be associated with female sex hormones (40).

In 2019, the Chinese incidence cases and death cases from many types of GI cancers accounted for a significantly increasing proportion of the global patients. Reassuringly, the incidence and death rates of esophageal, stomach, and liver cancers, which are traditionally high in China, are declining gradually in recent years. Along with pancreatic and colon and rectum cancers becoming more prevalent, the GI cancer situation in China is evolving to resemble that of developed countries (4). Health managers are therefore able to derive experience from the cancer prevention achievements of these countries. The National Central Cancer Registry (NCCR) of China was founded in 2002, perhaps contributing to the V-shaped change in death and DALY rates during that period (41). We speculate that the earlier data are potentially underestimated, thus reflecting the tremendous success of gastrointestinal cancer prevention and treatment in China over the last 30 years. A particularly typical example is the series of HBV prevention policies, such as immunization and promotion of safe injection practices, which have reduced HBV infection and consequent liver cancer (42). In addition, China is striving to promote early screening and precision diagnosis and treatment, taking the prevention and management of GI cancers to a new dimension (43–45). With the economic transition, population growth, and accelerated rate of aging, it is undeniable that the burden of GI cancers would become increasingly serious in China. More efforts are obligatory for China to provide reasonable and effective interventions in the future, such as improving the dietary habits of the population, focusing on strengthening the control of risk factors, and targeted cancer screening.

In conclusion, nowadays, gastrointestinal cancers are a considerable contributor to the cancer burden in China and even globally, and pose numerous challenges for public health management. Given that GI cancer is an aging-related disease, the future burden might be increasingly heavy in the context of global aging (46). Therefore, primary and secondary prevention measures are especially important considering the serious consequences that GI cancer would bring to patients and the healthcare system.

Some limitations of this study should be stated. First, there are inherent biases in the GBD database, such as the uneven quality of data from different national sources. Within the same country, representative data are potentially underestimated because of the omission of health surveys in remote areas. Second, there are multiple risk factors for gastrointestinal cancers, including environment, infection, age, smoking, alcohol consumption, diet, chronic disease, and psychological stress, of which GBD 2019 describes only a small proportion, thus other factors are required for further investigation in the future (47–49). Finally, we neglected to explore the effect of viruses on liver cancer since it had been reported by studies (28).

## Conclusion

Gastrointestinal cancers are evolving increasingly prevalent along with serious consequences, and pose numerous challenges to public health management in China and globally. Targeted primary and secondary prevention measures are particularly important in the future.

## Data Availability Statement

Publicly available datasets were analyzed in this study. This data can be found here: The Global Burden of Disease Study (GBD) 2019 by utilizing the Global Health Data Exchange (GHDx) query tool (http://ghdx.healthdata.org/gbd-results-tool).

## Ethics Statement

Written informed consent for participation was not provided by the participants' legal guardians/next of kin because this study was based on a publicly available database and exempted by the Ethics Committee of Shanghai Tenth People's Hospital.

## Author Contributions

ZS and WX contributed to the conceptualization and design of the study. WY and ZH contributed to the acquisition of data. WX, TY, and JZ contributed to the analysis and interpretation of data. WX drafted the manuscript. ZS and ZM carried out the writing—review and editing. All authors have contributed to the article and agreed to submit this version.

## Conflict of Interest

The authors declare that the research was conducted in the absence of any commercial or financial relationships that could be construed as a potential conflict of interest.

## Publisher's Note

All claims expressed in this article are solely those of the authors and do not necessarily represent those of their affiliated organizations, or those of the publisher, the editors and the reviewers. Any product that may be evaluated in this article, or claim that may be made by its manufacturer, is not guaranteed or endorsed by the publisher.
