# Supplementary material for: Chinese and Global Burdens of Gastrointestinal Cancers From 1990 to 2019
Source: Front Public Health. 2022 Jul 13;10:941284. doi: 10.3389/fpubh.2022.941284 (PMC9326121; doi:10.3389/fpubh.2022.941284)
Supplement: Supplementary file 1 [file Data_Sheet_1.ZIP › Supplementary Material/Supplementary Material.docx]

***Supplementary Material***

**Table s1. The incident cases and age-standardized incidence rate of esophageal cancer in 1990 and 2019, and its temporal trends from 1990 to 2019.**

| **Characteristics** | **1990** | | **2019** | | **1990-2019** |
| --- | --- | --- | --- | --- | --- |
|  | **Incident cases**  **No. ×10^3^ (95% UI)** | **ASIR per 100,000**  **No. (95% UI)** | **Incident cases**  **No. ×10^3^ (95% UI)** | **ASIR per 100,000**  **No. (95% UI)** | **EAPC**  **No. (95% CI)** |
| **Global** | 320.0 (253.4-351.2) | 8.1 (6.4-8.8) | 534.6 (466.5-595.3) | 6.5 (5.7-7.2) | -0.90 (-1.18--0.62) |
| **Male** | 216.1 (171.8-241.5) | 11.7 (9.3-13.1) | 388.8 (335.5-444.0) | 10.1 (8.7-11.6) | -0.59 (-0.85--0.33) |
| **Female** | 103.9 (74.9-116.8) | 4.9 (3.6-5.5) | 145.7 (120.0-165.1) | 3.3 (2.7-3.8) | -1.65 (-1.98--1.32) |
| **China** | 173.7 (112.1-203.4) | 21.0 (13.6-24.3) | 278.1 (213.5-331.6) | 13.9 (10.7-16.5) | -1.58 (-2.03--1.13) |
| **Male** | 115.5 (75.1-140.4) | 28.7 (19.2-34.4) | 207.9 (156.1-258.3) | 21.9 (16.4-27.0) | -0.96 (-1.35--0.56) |
| **Female** | 58.2 (32.2-69.8) | 13.9 (7.8-16.6) | 70.2 (46.0-88.6) | 6.8 (4.4-8.6) | -2.85 (-3.42--2.28) |
| **High SDI** | 52.2 (50.6-53.2) | 5.1 (4.9-5.2) | 95.9 (86.7-105.1) | 5.2 (4.6-5.7) | 0 (-0.14-0.14) |
| **High-middle SDI** | 86.7 (73.3-95.0) | 8.1 (6.9-8.8) | 145.3(113.2-169.3) | 7.1 (5.5-8.2) | -0.61 (-0.88--0.34) |
| **Middle SDI** | 136.2 (81.4-157.4) | 13.5 (8.1-15.5) | 205.2 (164.6-237.9) | 8.4 (6.7-9.7) | -1.83 (-2.25--1.42) |
| **Low-middle SDI** | 30.4 (26.9-39.3) | 5.1 (4.5-6.5) | 59.9 (52.7-84.1) | 4.4 (3.9-6.2) | -0.64 (-0.73--0.55) |
| **Low SDI** | 14.4 (11.9-16.6) | 6.0 (5.0-6.9) | 28.1 (23.2-33.4) | 5.4 (4.5-6.4) | -0.48 (-0.53--0.42) |
| **Andean Latin America** | 0.39 (0.33-0.44) | 2.0 (1.6-2.2) | 0.83 (0.67-1.02) | 1.5 (1.2-1.9) | -0.85 (-0.94--0.76) |
| **Australasia** | 1.1 (1.0-1.1) | 4.6 (4.4-4.8) | 2.2 (1.8-2.7) | 4.4 (3.5-5.5) | -0.30 (-0.39--0.20) |
| **Caribbean** | 1.0 (0.9-1.1) | 3.8 (3.6-4.1) | 1.9 (1.6-2.2) | 3.7 (3.2-4.2) | 0.07 (-0.13-0.27) |
| **Central Asia** | 6.4 (6.2-6.7) | 13.8 (13.3-14.3) | 4.8 (4.3-5.7) | 6.7 (6.0-7.8) | -2.85 (-3.08--2.61) |
| **Central Europe** | 4.3 (4.2-4.4) | 2.9 (2.8-3.0) | 5.9 (5.1-6.7) | 2.9 (2.5-3.3) | -0.17 (-0.27--0.07) |
| **Central Latin America** | 1.9 (1.8-2.0) | 2.4 (2.3-2.5) | 3.9 (3.3-4.5) | 1.7 (1.4-1.9) | -1.48 (-1.60--1.37) |
| **Central Sub-Saharan Africa** | 2.5 (1.1-3.3) | 10.8 (5.0-14.3) | 4.4 (2.4-6.0) | 8.4 (4.5-11.6) | -1.09 (-1.18--1.00) |
| **East Asia** | 176.2 (114.4-205.9) | 20.5 (13.4-23.7) | 284.9 (220.2-338.9) | 13.7 (10.6-16.3) | -1.54 (-1.98--1.09) |
| **Eastern Europe** | 12.2 (11.7-12.9) | 4.3 (4.1-4.5) | 11.1 (9.7-12.6) | 3.2 (2.8-3.7) | -1.38 (-1.62--1.14) |
| **Eastern Sub-Saharan Africa** | 8.4 (6.4-9.9) | 11.2 (8.5-13.0) | 16.4 (12.4-20.7) | 10.0 (7.7-12.6) | -0.46 (-0.54--0.38) |
| **High-income Asia Pacific** | 13.2 (12.6-13.6) | 6.5 (6.2-6.7) | 25.2 (21.2-29.6) | 5.7 (4.8-6.8) | -0.57 (-0.74--0.40) |
| **High-income North America** | 13.2 (12.8-13.6) | 3.9 (3.7-4.0) | 26.2 (22.5-30.6) | 4.2 (3.6-5.0) | 0.19 (0.07-0.31) |
| **North Africa and Middle East** | 4.4 (3.1-5.2) | 2.5 (1.8-3.0) | 10.0 (7.4-11.4) | 2.4 (1.8-2.7) | -0.32 (-0.36--0.28) |
| **Oceania** | 0.06 (0.05-0.09) | 2.2 (1.7-3.1) | 0.15 (0.11-0.20) | 2.2 (1.7-2.9) | -0.05 (-0.08--0.03) |
| **South Asia** | 25.6 (22.5-32.1) | 4.5 (4.0-5.7) | 53.5 (46.2-72.1) | 3.8 (3.3-5.1) | -0.86 (-0.98--0.73) |
| **Southeast Asia** | 7.1 (6.0-8.1) | 2.7 (2.4-3.1) | 15.5 (13.2-18.2) | 2.5 (2.2-3.0) | -0.34 (-0.39--0.30) |
| **Southern Latin America** | 3.4 (3.3-3.5) | 7.4 (7.1-7.7) | 3.9 (3.2-4.9) | 4.7 (3.8-5.9) | -1.93 (-2.09--1.77) |
| **Southern Sub-Saharan Africa** | 3.7 (2.8-4.6) | 13.3 (9.9-16.5) | 5.9 (5.3-6.9) | 10.7 (9.6-12.3) | -1.36 (-1.89--0.81) |
| **Tropical Latin America** | 6.1 (5.9-6.4) | 6.7 (6.4-6.9) | 12.7 (12.0-13.3) | 5.2 (4.9-5.4) | -0.84 (-0.89--0.79) |
| **Western Europe** | 27.0 (26.2-27.5) | 4.8 (4.7-4.9) | 40.2 (35.1-45.7) | 4.6 (4.1-5.3) | -0.23 (-0.34--0.12) |
| **Western Sub-Saharan Africa** | 1.8 (1.6-2.1) | 2.1 (1.8-2.4) | 5.0 (3.8-6.0) | 2.7 (2.1-3.2) | 1.16 (1.04-1.28) |

ASIR, age-standardized incidence rate; CI, confidence interval; EAPC, estimated annual percentage change; SDI, socio-demographic index; UI, uncertainty interval.

**Table s2. The incident cases and age-standardized incidence rate of stomach cancer in 1990 and 2019, and its temporal trends from 1990 to 2019.**

| **Characteristics** | **1990** | | **2019** | | **1990-2019** |
| --- | --- | --- | --- | --- | --- |
|  | **Incident cases**  **No. ×10^3^ (95% UI)** | **ASIR per 100,000**  **No. (95% UI)** | **Incident cases**  **No. ×10^3^ (95% UI)** | **ASIR per 100,000**  **No. (95% UI)** | **EAPC**  **No. (95% CI)** |
| **Global** | 883.4 (834.2-929.2) | 22.4 (21.2-23.6) | 1269.8 (1150.5-1399.8) | 15.6 (14.1-17.2) | -1.22 (-1.35--1.09) |
| **Male** | 548.1 (509.5-588.0) | 30.4 (28.4-32.5) | 846.9 (748.2-963.1) | 22.4 (19.8-25.3) | -0.96 (-1.11--0.81) |
| **Female** | 335.3 (312.4-356.9) | 15.8 (14.7-16.8) | 422.9 (377.4-467.1) | 9.7 (8.7-10.7) | -1.76 (-1.87--1.65) |
| **China** | 317.3 (277.9-359.3) | 37.6 (33.1-42.3) | 612.8 (513.0-728.9) | 30.6 (25.8-36.1) | -0.41 (-0.75--0.08) |
| **Male** | 207.5 (173.2-245.6) | 51.1 (43.0-59.9) | 451.3 (357.2-560.1) | 47.3 (38.0-57.9) | 0.14 (-0.19-0.48) |
| **Female** | 109.8 (92.1-127.9) | 25.6 (21.5-29.6) | 161.5 (130.6-198.3) | 15.8 (12.8-19.4) | -1.6 (-1.93--1.26) |
| **High SDI** | 227.6 (219.7-232.0) | 22.0 (21.2-22.4) | 237.8 (209.7-260.9) | 12.4 (11.1-13.6) | -2.12 (-2.18--2.06) |
| **High-middle SDI** | 291.6 (275.0-308.0) | 27.3 (25.7-28.8) | 381.1 (337.3-427.2) | 18.8 (16.6-21.0) | -1.25 (-1.41--1.08) |
| **Middle SDI** | 251.4 (226.7-278.0) | 24.5 (22.2-27.1) | 458.2 (398.8-527.4) | 18.7 (16.4-21.4) | -0.77 (-1.00--0.54) |
| **Low-middle SDI** | 85.8 (78.5-92.3) | 14.2 (13.0-15.2) | 149.2 (136.1-163.4) | 11.0 (10.0-12.0) | -0.89 (-0.97--0.81) |
| **Low SDI** | 26.8 (23.6-29.6) | 11.3 (10.0-12.5) | 43.2 (38.6-48.1) | 8.4 (7.6-9.3) | -1.06 (-1.09--1.02) |
| **Andean Latin America** | 6.0 (5.4-6.5) | 29.7 (26.9-32.4) | 12.4 (10.1-15.0) | 22.4 (18.3-27.2) | -0.98 (-1.08--0.88) |
| **Australasia** | 2.4 (2.3-2.5) | 10.2 (9.7-10.6) | 3.4 (2.8-4.2) | 7.0 (5.7-8.5) | -1.42 (-1.50--1.34) |
| **Caribbean** | 2.9 (2.7-3.1) | 11.3 (10.3-12.0) | 4.4 (3.8-5.0) | 8.4 (7.3-9.6) | -0.90 (-1.00--0.79) |
| **Central Asia** | 13.4 (13.0-13.8) | 28.0 (27.1-28.8) | 12.1 (11.0-13.4) | 16.4 (15.0-17.9) | -1.82 (-1.95--1.70) |
| **Central Europe** | 26.5 (25.8-26.9) | 18.1 (17.6-18.4) | 21.7 (19.1-24.4) | 10.3 (9.0-11.6) | -1.98 (-2.04--1.93) |
| **Central Latin America** | 15.5 (15.0-15.9) | 19.0 (18.2-19.6) | 30.5 (26.0-35.8) | 13.0 (11.1-15.2) | -1.60 (-1.69--1.51) |
| **Central Sub-Saharan Africa** | 2.7 (2.2-3.3) | 11.9 (9.9-14.1) | 4.3 (3.4-5.3) | 8.0 (6.5-9.8) | -1.45 (-1.50--1.41) |
| **East Asia** | 325.7 (285.5-367.3) | 37.1 (32.7-41.7) | 626.5 (526.6-741.3) | 30.2 (25.5-35.5) | -0.43 (-0.76--0.10) |
| **Eastern Europe** | 87.0 (84.2-88.7) | 30.9 (29.8-31.5) | 54.1 (48.8-59.8) | 16.1 (14.5-17.8) | -2.66 (-2.85--2.46) |
| **Eastern Sub-Saharan Africa** | 8.2 (7.1-9.2) | 10.7 (9.3-11.8) | 11.8 (10.2-13.4) | 7.2 (6.3-8.2) | -1.53 (-1.60--1.46) |
| **High-income Asia Pacific** | 123.7 (119.6-126.5) | 61.5 (59.3-63.0) | 128.2 (108.5-147.7) | 28.2 (24.2-32.3) | -2.82 (-2.90--2.74) |
| **High-income North America** | 30.3 (29.1-31.1) | 8.5 (8.2-8.7) | 37.6 (33.0-42.7) | 6.1 (5.4-7.0) | -1.33 (-1.41--1.26) |
| **North Africa and Middle East** | 23.5 (20.5-25.7) | 13.7 (11.9-15.0) | 42.3 (38.2-46.7) | 10.1 (9.1-11.1) | -0.94 (-1.15--0.73) |
| **Oceania** | 0.42 (0.33-0.51) | 13.8 (11.0-16.6) | 0.94 (0.71-1.19) | 12.9 (10.1-15.9) | -0.23 (-0.27--0.19) |
| **South Asia** | 57.6 (52.0-63.2) | 9.9 (8.9-11.0) | 99.4 (87.3-113.6) | 7.0 (6.2-8.0) | -1.31 (-1.39--1.23) |
| **Southeast Asia** | 28.1 (24.3-31.1) | 10.9 (9.5-12.1) | 40.1 (35.5-44.8) | 6.7 (6.0-7.5) | -1.90 (-1.98--1.81) |
| **Southern Latin America** | 8.5 (8.2-8.7) | 18.5 (17.9-19.1) | 10.7 (8.6-13.3) | 12.8 (10.2-16.0) | -1.29 (-1.36--1.23) |
| **Southern Sub-Saharan Africa** | 2.4 (2.2-2.6) | 8.7 (7.9-9.5) | 3.6 (3.3-4.0) | 6.5 (6.0-7.1) | -1.16 (-1.49--0.83) |
| **Tropical Latin America** | 16.1 (15.5-16.6) | 18.1 (17.2-18.7) | 24.5 (23.1-25.7) | 10.2 (9.6-10.7) | -2.03 (-2.11--1.95) |
| **Western Europe** | 94.0 (90.0-96.1) | 16.1 (15.5-16.5) | 86.5 (75.2-97.3) | 9.4 (8.2-10.7) | -1.96 (-2.02--1.90) |
| **Western Sub-Saharan Africa** | 8.5 (7.4-9.6) | 10.2 (8.9-11.4) | 15.0 (12.9-17.4) | 8.7 (7.5-9.8) | -0.36 (-0.44--0.28) |

ASIR, age-standardized incidence rate; CI, confidence interval; EAPC, estimated annual percentage change; SDI, socio-demographic index; UI, uncertainty interval.

**Table s3. The incident cases and age-standardized incidence rate of liver cancer in 1990 and 2019, and its temporal trends from 1990 to 2019.**

| **Characteristics** | **1990** | | **2019** | | **1990-2019** |
| --- | --- | --- | --- | --- | --- |
|  | **Incident cases**  **No. ×10^3^ (95% UI)** | **ASIR per 100,000**  **No. (95% UI)** | **Incident cases**  **No. ×10^3^ (95% UI)** | **ASIR per 100,000**  **No. (95% UI)** | **EAPC**  **No. (95% CI)** |
| **Global** | 373.4 (335.9-415.7) | 9.0 (8.1-10.0) | 534.4 (486.5-588.6) | 6.5 (5.9-7.2) | -1.93 (-2.29--1.57) |
| **Male** | 260.2 (226.9-297.8) | 13.1 (11.4-14.9) | 376.5 (335.0-422.0) | 9.7 (8.7-10.8) | -1.92 (-2.31--1.52) |
| **Female** | 113.2 (99.8-129.3) | 5.2 (4.6-6.0) | 157.9 (140.4-176.1) | 3.6 (3.2-4.0) | -1.87 (-2.14--1.60) |
| **China** | 236.8 (199.3-280.1) | 25.7 (21.7-30.3) | 210.5 (174.8-251.2) | 10.5 (8.7-12.4) | -4.67 (-5.47--3.88) |
| **Male** | 170.1 (137.5-206.7) | 36.4 (29.6-44.2) | 159.8 (127.3-198.8) | 16.4 (13.1-20.2) | -4.40 (-5.23--3.57) |
| **Female** | 66.7 (54.2-82.0) | 15.0 (12.3-18.3) | 50.7 (40.9-62.3) | 4.9 (4.0-6.1) | -5.16 (-5.83--4.48) |
| **High SDI** | 53.5 (51.8-54.7) | 5.3 (5.1-5.4) | 140.1 (125.5-154.0) | 7.6 (6.9-8.4) | 0.91 (0.49-1.33) |
| **High-middle SDI** | 108.7 (95.2-123.1) | 9.9 (8.7-11.2) | 106.9 (94.2-121.0) | 5.3 (4.7-6.0) | -3.33 (-3.86--2.79) |
| **Middle SDI** | 166.1 (144.1-193.6) | 14.7 (12.8-17.0) | 210.5 (184.2-242.2) | 8.3 (7.2-9.5) | -2.98 (-3.54--2.41) |
| **Low-middle SDI** | 34.7 (31.3-38.6) | 5.4 (4.9-5.9) | 56.3 (51.0-62.6) | 4.1 (3.7-4.5) | -1.59 (-1.89--1.28) |
| **Low SDI** | 10.2 (9.0-11.6) | 4.1 (3.6-4.6) | 20.3 (17.8-22.8) | 3.7 (3.3-4.1) | -0.46 (-0.52--0.40) |
| **Andean Latin America** | 1.0 (0.9-1.2) | 4.9 (4.3-5.5) | 1.7 (1.4-2.1) | 3.1 (2.5-3.8) | -2.01 (-2.44--1.58) |
| **Australasia** | 0.48 (0.46-0.49) | 2.0 (2.0-2.1) | 2.2 (1.8-2.7) | 4.6 (3.7-5.7) | 3.14 (2.88-3.39) |
| **Caribbean** | 1.6 (1.5-1.7) | 5.9 (5.5-6.3) | 1.6 (1.4-1.9) | 3.2 (2.6-3.8) | -2.09 (-2.85--1.32) |
| **Central Asia** | 1.5 (1.3-1.7) | 3.1 (2.8-3.5) | 6.1 (5.3-7.0) | 8.3 (7.2-9.4) | 2.78 (2.25-3.30) |
| **Central Europe** | 7.7 (7.4-7.9) | 5.3 (5.1-5.4) | 6.9 (6.0-8.0) | 3.3 (2.9-3.8) | -1.32 (-1.67--0.97) |
| **Central Latin America** | 3.0 (2.8-3.1) | 3.5 (3.2-3.7) | 8.0 (6.9-9.3) | 3.4 (3.0-4.0) | 0.11 (-0.21-0.42) |
| **Central Sub-Saharan Africa** | 0.69 (0.56-0.83) | 2.6 (2.2-3.1) | 1.4 (1.1-1.7) | 2.3 (1.8-2.9) | -0.61 (-0.68--0.54) |
| **East Asia** | 241.5 (204.2-284.8) | 25.3 (21.5-29.7) | 217.2 (181.4-257.5) | 10.4 (8.8-12.3) | -4.60 (-5.35--3.83) |
| **Eastern Europe** | 4.1 (4.0-4.3) | 1.5 (1.5-1.6) | 9.4 (8.2-10.7) | 2.8 (2.5-3.2) | 2.52 (2.28-2.77) |
| **Eastern Sub-Saharan Africa** | 2.4 (2.0-3.0) | 2.9 (2.4-3.7) | 5.4 (4.5-6.7) | 3.1 (2.6-3.8) | -0.03 (-0.15-0.10) |
| **High-income Asia Pacific** | 28.2 (27.3-29.1) | 13.8 (13.3-14.2) | 67.9 (58.1-77.6) | 15.6 (13.5-17.7) | -0.17 (-0.71-0.37) |
| **High-income North America** | 7.5 (7.3-7.7) | 2.2 (2.1-2.3) | 31.0 (25.7-37.0) | 5.2 (4.3-6.2) | 2.99 (2.80-3.19) |
| **North Africa and Middle East** | 10.7 (9.4-12.0) | 6.1 (5.3-6.8) | 27.5 (22.1-33.8) | 6.3 (5.1-7.7) | 0.49 (0.32-0.66) |
| **Oceania** | 0.11 (0.10-0.13) | 3.7 (3.1-4.2) | 0.23 (0.20-0.28) | 3.3 (2.8-3.9) | -0.22 (-0.27--0.17) |
| **South Asia** | 15.7 (13.2-18.0) | 2.7 (2.2-3.1) | 37.7 (32.8-43.3) | 2.7 (2.3-3.1) | -0.02 (-0.09-0.06) |
| **Southeast Asia** | 17.3 (15.4-19.0) | 6.4 (5.7-7.1) | 42.8 (35.2-52.1) | 7.1 (5.9-8.6) | 0.31 (0.26-0.36) |
| **Southern Latin America** | 0.72 (0.65-0.79) | 1.6 (1.4-1.7) | 1.9 (1.5-2.4) | 2.3 (1.8-2.9) | 2.05 (1.82-2.29) |
| **Southern Sub-Saharan Africa** | 1.9 (1.3-3.2) | 6.5 (4.5-10.7) | 4.0 (3.6-4.5) | 6.8 (6.1-7.6) | -0.43 (-0.97-0.11) |
| **Tropical Latin America** | 1.8 (1.8-1.9) | 2.0 (1.9-2.0) | 5.7 (5.3-6.0) | 2.4 (2.2-2.5) | 1.05 (0.90-1.20) |
| **Western Europe** | 20.2 (19.5-20.7) | 3.6 (3.4-3.6) | 45.9 (39.8-52.7) | 5.3 (4.6-6.1) | 1.37 (1.19-1.55) |
| **Western Sub-Saharan Africa** | 5.1 (4.3-6.0) | 5.4 (4.6-6.3) | 9.7 (8.2-11.4) | 4.9 (4.2-5.7) | -0.47 (-0.54--0.40) |

ASIR, age-standardized incidence rate; CI, confidence interval; EAPC, estimated annual percentage change; SDI, socio-demographic index; UI, uncertainty interval.

**Table s4. The incident cases and age-standardized incidence rate of pancreatic cancer in 1990 and 2019, and its temporal trends from 1990 to 2019.**

| **Characteristics** | **1990** | | **2019** | | **1990-2019** |
| --- | --- | --- | --- | --- | --- |
|  | **Incident cases**  **No. ×10^3^ (95% UI)** | **ASIR per 100,000**  **No. (95% UI)** | **Incident cases**  **No. ×10^3^ (95% UI)** | **ASIR per 100,000**  **No. (95% UI)** | **EAPC**  **No. (95% CI)** |
| **Global** | 197.3 (188.6-204.0) | 5.2 (5.0-5.4) | 530.3 (486.2-573.6) | 6.6 (6.0-7.1) | 0.83 (0.78-0.87) |
| **Male** | 104.1 (99.2-109.0) | 6.0 (5.7-6.3) | 279.9 (256.0-303.4) | 7.5 (6.8-8.1) | 0.84 (0.79-0.90) |
| **Female** | 93.3 (88.5-96.9) | 4.5 (4.3-4.7) | 250.4 (223.8-275.4) | 5.7 (5.1-6.3) | 0.81 (0.77-0.85) |
| **China** | 26.8 (23.1-30.3) | 3.2 (2.8-3.6) | 115.0 (98.0-133.7) | 5.8 (4.9-6.7) | 2.32 (2.13-2.51) |
| **Male** | 15.8 (12.8-19.1) | 3.9 (3.2-4.6) | 69.6 (55.2-86.1) | 7.4 (6.0-9.1) | 2.64 (2.44-2.84) |
| **Female** | 10.9 (9.2-12.7) | 2.6 (2.2-3.0) | 45.3 (36.4-55.8) | 4.4 (3.5-5.4) | 1.88 (1.71-2.06) |
| **High SDI** | 91.2 (87.2-93.1) | 8.7 (8.3-8.9) | 196.9 (174.8-215.5) | 10.2 (9.1-11.1) | 0.62 (0.58-0.67) |
| **High-middle SDI** | 64.8 (62.4-67.2) | 6.1 (5.9-6.4) | 156.5 (142.6-170.4) | 7.7 (7.0-8.3) | 0.74 (0.66-0.83) |
| **Middle SDI** | 27.4 (25.5-29.4) | 2.7 (2.5-2.9) | 117.1 (104.6-130.6) | 4.8 (4.3-5.3) | 2.03 (1.97-2.10) |
| **Low-middle SDI** | 10.3 (8.9-11.8) | 1.8 (1.5-2.0) | 46.9 (43.0-51.0) | 3.5 (3.2-3.8) | 2.40 (2.36-2.45) |
| **Low SDI** | 3.6 (2.9-4.3) | 1.6 (1.3-1.9) | 12.6 (11.0-14.2) | 2.5 (2.2-2.9) | 1.67 (1.62-1.72) |
| **Andean Latin America** | 0.40 (0.35-0.45) | 2.0 (1.7-2.2) | 2.9 (2.3-3.5) | 5.2 (4.3-6.3) | 3.64 (3.01-4.27) |
| **Australasia** | 1.8 (1.7-1.9) | 7.8 (7.4-8.0) | 4.4 (3.6-5.4) | 8.7 (7.0-10.7) | 0.45 (0.38-0.52) |
| **Caribbean** | 0.42 (0.40-0.44) | 1.6 (1.5-1.7) | 2.6 (2.2-3.1) | 5.1 (4.3-6.0) | 3.84 (3.05-4.63) |
| **Central Asia** | 1.2 (1.0-1.3) | 2.5 (2.2-2.8) | 4.1 (3.8-4.5) | 5.8 (5.3-6.4) | 3.66 (3.27-4.04) |
| **Central Europe** | 12.3 (11.9-12.6) | 8.3 (8.1-8.5) | 22.0 (19.4-24.8) | 10.3 (9.1-11.7) | 0.79 (0.71-0.87) |
| **Central Latin America** | 3.5 (3.4-3.6) | 4.4 (4.2-4.5) | 12.5 (10.8-14.4) | 5.4 (4.6-6.2) | 0.45 (0.33-0.56) |
| **Central Sub-Saharan Africa** | 0.48 (0.38-0.60) | 2.2 (1.7-2.7) | 1.4 (1.1-1.8) | 2.7 (2.2-3.4) | 0.54 (0.26-0.82) |
| **East Asia** | 27.9 (24.3-31.5) | 3.2 (2.8-3.6) | 119.6 (102.2-138.5) | 5.8 (5.0-6.7) | 2.31 (2.13-2.50) |
| **Eastern Europe** | 18.9 (17.9-20.1) | 6.7 (6.4-7.1) | 27.3 (24.7-30.3) | 8.0 (7.2-8.8) | 0.28 (0.04-0.52) |
| **Eastern Sub-Saharan Africa** | 1.4 (1.2-1.6) | 1.9 (1.6-2.2) | 4.5 (3.8-5.2) | 2.8 (2.4-3.3) | 1.41 (1.35-1.47) |
| **High-income Asia Pacific** | 18.8 (17.9-19.3) | 9.5 (9.0-9.8) | 49.5 (41.2-56.4) | 10.2 (8.7-11.7) | 0.37 (0.27-0.48) |
| **High-income North America** | 31.9 (30.3-32.7) | 9.0 (8.6-9.2) | 65.3 (57.3-74.2) | 10.3 (9.1-11.7) | 0.50 (0.47-0.53) |
| **North Africa and Middle East** | 4.5 (3.8-5.3) | 2.7 (2.3-3.2) | 22.2 (19.3-25.7) | 5.3 (4.6-6.1) | 2.50 (2.33-2.67) |
| **Oceania** | 0.05 (0.04-0.06) | 1.8 (1.4-2.2) | 0.17 (0.13-0.21) | 2.5 (2.0-3.1) | 1.09 (0.97-1.21) |
| **South Asia** | 7.6 (6.1-8.9) | 1.4 (1.1-1.7) | 38.7 (34.0-43.7) | 2.9 (2.5-3.2) | 2.45 (2.35-2.56) |
| **Southeast Asia** | 6.0 (5.4-6.5) | 2.4 (2.2-2.6) | 25.4 (20.3-31.5) | 4.3 (3.5-5.3) | 2.04 (2.00-2.08) |
| **Southern Latin America** | 3.9 (3.7-4.2) | 8.6 (8.0-9.3) | 8.8 (7.0-11.0) | 10.5 (8.3-13.1) | 0.55 (0.40-0.70) |
| **Southern Sub-Saharan Africa** | 1.2 (1.0-1.4) | 4.4 (3.8-5.3) | 3.3 (3.0-3.7) | 6.1 (5.5-6.7) | 0.93 (0.63-1.22) |
| **Tropical Latin America** | 4.5 (4.3-4.6) | 5.1 (4.9-5.3) | 14.8 (13.6-15.6) | 6.2 (5.7-6.5) | 0.73 (0.68-0.78) |
| **Western Europe** | 48.8 (46.9-49.9) | 8.4 (8.1-8.6) | 92.8 (80.3-105.2) | 10.0 (8.7-11.4) | 0.69 (0.61-0.78) |
| **Western Sub-Saharan Africa** | 1.9 (1.6-2.2) | 2.2 (1.9-2.6) | 7.9 (6.5-9.4) | 4.5 (3.8-5.3) | 2.44 (2.39-2.50) |

ASIR, age-standardized incidence rate; CI, confidence interval; EAPC, estimated annual percentage change; SDI, socio-demographic index; UI, uncertainty interval.

**Table s5. The incident cases and age-standardized incidence rate of colon and rectum cancer in 1990 and 2019, and its temporal trends from 1990 to 2019.**

| **Characteristics** | **1990** | | **2019** | | **1990-2019** |
| --- | --- | --- | --- | --- | --- |
|  | **Incident cases**  **No. ×10^3^ (95% UI)** | **ASIR per 100,000**  **No. (95% UI)** | **Incident cases**  **No. ×10^3^ (95% UI)** | **ASIR per 100,000**  **No. (95% UI)** | **EAPC**  **No. (95% CI)** |
| **Global** | 842.1 (810.4-868.6) | 22.2 (21.3-23.0) | 2166.2 (1996.3-2342.8) | 26.7 (24.6-28.9) | 0.58 (0.52-0.65) |
| **Male** | 428.2 (413.5-444.3) | 25.2 (24.2-26.1) | 1239.7 (1133.2-1359.1) | 33.1 (30.2-36.2) | 0.95 (0.87-1.02) |
| **Female** | 413.9 (393.4-432.7) | 19.9 (18.8-20.8) | 926.4 (831.9-1011.6) | 21.2 (19.0-23.2) | 0.11 (0.03-0.18) |
| **China** | 105.9 (93.8-119.0) | 12.5 (11.2-14.0) | 607.9 (521.8-708.4) | 30.6 (26.4-35.5) | 3.66 (3.37-3.95) |
| **Male** | 56.9 (47.5-66.8) | 14.2 (12.0-16.4) | 390.2 (310.2-484.1) | 41.4 (33.4-50.9) | 4.46 (4.13-4.80) |
| **Female** | 49.0 (40.8-57.1) | 11.3 (9.5-13.1) | 217.7 (175.1-268.4) | 21.1 (17.0-26.0) | 2.50 (2.27-2.73) |
| **High SDI** | 443.9 (427.7-453.3) | 42.5 (40.9-43.4) | 798.6 (715.6-873.2) | 42.8 (38.7-46.6) | -0.17 (-0.28--0.06) |
| **High-middle SDI** | 237.9 (229.8-246.1) | 22.6 (21.8-23.4) | 656.8 (596.0-717.9) | 32.5 (29.5-35.4) | 1.29 (1.19-1.40) |
| **Middle SDI** | 104.8 (97.3-113.1) | 10.2 (9.5-11.0) | 517.6 (463.5-578.2) | 20.9 (18.8-23.3) | 2.77 (2.63-2.92) |
| **Low-middle SDI** | 40.8 (36.8-45.7) | 6.9 (6.2-7.7) | 153.1 (138.8-168.2) | 11.4 (10.3-12.4) | 1.74 (1.70-1.79) |
| **Low SDI** | 14.3 (12.0-16.8) | 6.2 (5.2-7.3) | 39.0 (34.9-43.4) | 7.7 (6.9-8.6) | 0.79 (0.71-0.86) |
| **Andean Latin America** | 2.0 (1.8-2.2) | 10.0 (8.8-11.1) | 11.1 (8.9-13.5) | 20.0 (16.1-24.2) | 2.70 (2.52-2.88) |
| **Australasia** | 12.0 (11.5-12.4) | 51.6 (49.3-53.2) | 23.7 (19.4-28.8) | 48.3 (39.6-59.1) | -0.52 (-0.65--0.39) |
| **Caribbean** | 4.7 (4.5-4.9) | 18.2 (17.3-18.8) | 13.8 (11.8-16.0) | 26.7 (22.9-30.9) | 1.43 (1.37-1.49) |
| **Central Asia** | 6.7 (6.5-7.0) | 14.0 (13.5-14.5) | 10.9 (10.0-12.0) | 15.2 (13.9-16.6) | 0.54 (0.33-0.76) |
| **Central Europe** | 41.6 (40.4-42.6) | 28.4 (27.5-29.1) | 84.5 (74.6-95.5) | 39.9 (35.2-45.1) | 1.23 (1.11-1.35) |
| **Central Latin America** | 7.5 (7.2-7.7) | 9.0 (8.6-9.3) | 37.5 (32.2-43.9) | 15.9 (13.7-18.6) | 1.92 (1.83-2.02) |
| **Central Sub-Saharan Africa** | 1.6 (1.3-2.0) | 7.4 (5.9-9.3) | 4.0 (3.0-5.1) | 7.7 (5.9-10.1) | 0.06 (-0.20-0.32) |
| **East Asia** | 112.3 (100.3-125.6) | 12.8 (11.4-14.3) | 637.1 (548.9-738.5) | 30.9 (26.8-35.7) | 3.61 (3.33-3.89) |
| **Eastern Europe** | 70.4 (68.3-72.7) | 25.1 (24.3-25.9) | 106.0 (96.2-117.1) | 31.1 (28.2-34.4) | 0.56 (0.40-0.73) |
| **Eastern Sub-Saharan Africa** | 5.2 (4.3-6.1) | 7.0 (5.8-8.2) | 14.2 (12.1-16.9) | 8.8 (7.6-10.4) | 0.83 (0.75-0.91) |
| **High-income Asia Pacific** | 77.2 (74.0-79.2) | 38.7 (36.9-39.8) | 196.4 (166.4-225.6) | 44.6 (38.4-51.1) | 0.35 (0.23-0.47) |
| **High-income North America** | 167.9 (160.8-172.3) | 47.5 (45.6-48.6) | 260.9 (229.9-295.7) | 42.7 (37.6-48.6) | -0.62 (-0.72--0.51) |
| **North Africa and Middle East** | 15.4 (13.0-18.2) | 9.0 (7.6-10.6) | 60.0 (53.4-67.6) | 13.9 (12.3-15.6) | 1.71 (1.50-1.91) |
| **Oceania** | 0.25 (0.19-0.30) | 8.3 (6.6-9.9) | 0.69 (0.56-0.86) | 10.0 (8.2-12.1) | 0.55 (0.49-0.62) |
| **South Asia** | 29.9 (26.4-34.1) | 5.4 (4.8-6.2) | 113.7 (98.2-129.4) | 8.3 (7.2-9.4) | 1.31 (1.18-1.44) |
| **Southeast Asia** | 27.9 (24.6-30.7) | 10.8 (9.6-11.8) | 117.0 (96.6-136.2) | 19.3 (16.0-22.4) | 1.93 (1.87-1.98) |
| **Southern Latin America** | 10.9 (10.5-11.3) | 24.1 (23.1-24.8) | 26.9 (21.5-33.6) | 32.2 (25.7-40.4) | 0.94 (0.83-1.05) |
| **Southern Sub-Saharan Africa** | 2.9 (2.5-3.3) | 10.7 (9.3-12.7) | 7.1 (6.4-7.9) | 13.1 (11.8-14.5) | 0.70 (0.53-0.88) |
| **Tropical Latin America** | 10.7 (10.3-11.0) | 12.0 (11.5-12.4) | 42.9 (40.1-44.9) | 17.8 (16.6-18.6) | 1.44 (1.23-1.65) |
| **Western Europe** | 229.5 (220.4-234.9) | 39.6 (38.0-40.5) | 382.4 (332.8-432.4) | 42.4 (37.1-48.3) | 0.04 (-0.17-0.24) |
| **Western Sub-Saharan Africa** | 5.4 (4.4-6.6) | 6.5 (5.3-8.0) | 15.3 (12.9-17.8) | 8.7 (7.4-10.0) | 1.21 (1.10-1.32) |

ASIR, age-standardized incidence rate; CI, confidence interval; EAPC, estimated annual percentage change; SDI, socio-demographic index; UI, uncertainty interval.

**Table s6. The incident cases and age-standardized incidence rate of gallbladder and biliary tract cancer in 1990 and 2019, and its temporal trends from 1990 to 2019.**

| **Characteristics** | **1990** | | **2019** | | **1990-2019** |
| --- | --- | --- | --- | --- | --- |
|  | **Incident cases**  **No. ×10^3^ (95% UI)** | **ASIR per 100,000**  **No. (95% UI)** | **Incident cases**  **No. ×10^3^ (95% UI)** | **ASIR per 100,000**  **No. (95% UI)** | **EAPC**  **No. (95% CI)** |
| **Global** | 107.8 (96.9-119.9) | 2.9 (2.6-3.2) | 199.2 (166.8-219.6) | 2.5 (2.1-2.7) | -0.48 (-0.55--0.40) |
| **Male** | 40.4 (36.4-45.4) | 2.5 (2.2-2.7) | 86.4 (69.4-95.9) | 2.4 (1.9-2.7) | 0.04 (-0.06-0.15) |
| **Female** | 67.4 (57.8-78.7) | 3.3 (2.8-3.8) | 112.8 (91.6-129.8) | 2.6 (2.1-3.0) | -0.82 (-0.87--0.76) |
| **China** | 12.4 (10.3-20.0) | 1.6 (1.3-2.5) | 38.6 (27.4-46.5) | 2.0 (1.4-2.4) | 1.56 (1.12-2.00) |
| **Male** | 5.7 (4.4-9.5) | 1.6 (1.2-2.5) | 19.8 (13.2-24.9) | 2.2 (1.5-2.8) | 2.10 (1.68-2.53) |
| **Female** | 6.8 (5.3-11.6) | 1.6 (1.3-2.8) | 18.9 (11.3-24.7) | 1.8 (1.1-2.4) | 1.07 (0.62-1.53) |
| **High SDI** | 44.9 (36.4-47.0) | 4.2 (3.4-4.4) | 63.8 (52.1-72.3) | 3.2 (2.6-3.6) | -1.09 (-1.14--1.03) |
| **High-middle SDI** | 29.9 (26.7-32.4) | 2.9 (2.6-3.2) | 50.8 (37.7-57.5) | 2.5 (1.9-2.8) | -0.48 (-0.60--0.36) |
| **Middle SDI** | 18.3 (16.4-23.9) | 1.9 (1.7-2.5) | 45.8 (39.6-53.4) | 1.9 (1.7-2.2) | 0.23 (0.11-0.34) |
| **Low-middle SDI** | 10.9 (9.1-15.6) | 1.9 (1.6-2.7) | 29.6 (26.0-33.9) | 2.2 (2.0-2.6) | 0.58 (0.51-0.66) |
| **Low SDI** | 3.7 (2.9-4.9) | 1.6 (1.3-2.2) | 9.1 (7.7-10.5) | 1.9 (1.6-2.1) | 0.54 (0.45-0.64) |
| **Andean Latin America** | 1.1 (0.9-1.2) | 5.5 (4.6-6.2) | 2.5 (2.0-3.2) | 4.6 (3.6-5.8) | -0.68 (-0.80--0.56) |
| **Australasia** | 0.57 (0.51-0.66) | 2.4 (2.2-2.8) | 0.90 (0.71-1.09) | 1.8 (1.4-2.1) | -1.19 (-1.26--1.12) |
| **Caribbean** | 0.81 (0.52-0.91) | 3.2 (2.0-3.5) | 0.86 (0.71-1.08) | 1.7 (1.4-2.1) | -2.41 (-2.92--1.90) |
| **Central Asia** | 0.50 (0.43-0.59) | 1.1 (0.9-1.3) | 0.74 (0.61-0.86) | 1.1 (0.9-1.3) | -0.09 (-0.20-0.02) |
| **Central Europe** | 6.7 (5.7-7.2) | 4.6 (3.9-4.9) | 6.4 (5.4-7.4) | 2.9 (2.5-3.4) | -1.71 (-1.76--1.66) |
| **Central Latin America** | 3.5 (2.7-3.7) | 4.4 (3.3-4.7) | 5.9 (4.9-7.8) | 2.5 (2.1-3.3) | -2.27 (-2.42--2.12) |
| **Central Sub-Saharan Africa** | 0.25 (0.18-0.32) | 1.2 (0.9-1.5) | 0.50 (0.37-0.68) | 1.0 (0.8-1.4) | -0.48 (-0.53--0.42) |
| **East Asia** | 13.3 (11.1-21.1) | 1.6 (1.4-2.6) | 40.5 (29.2-48.5) | 2.0 (1.5-2.4) | 1.44 (1.02-1.86) |
| **Eastern Europe** | 4.7 (3.9-5.1) | 1.7 (1.4-1.9) | 5.1 (4.3-5.9) | 1.5 (1.3-1.7) | -0.77 (-0.96--0.57) |
| **Eastern Sub-Saharan Africa** | 0.96 (0.76-1.17) | 1.3 (1.1-1.6) | 1.8 (1.5-2.2) | 1.2 (1.0-1.5) | -0.33 (-0.38--0.29) |
| **High-income Asia Pacific** | 18.1 (14.8-19.1) | 9.3 (7.6-9.9) | 32.8 (24.2-38.6) | 6.4 (4.9-7.4) | -1.45 (-1.49--1.41) |
| **High-income North America** | 7.9 (6.6-8.4) | 2.2 (1.8-2.3) | 10.7 (9.1-13.1) | 1.7 (1.4-2.1) | -0.97 (-1.02--0.93) |
| **North Africa and Middle East** | 3.1 (2.5-3.8) | 1.9 (1.5-2.4) | 6.9 (6.0-8.3) | 1.7 (1.5-2.1) | -0.39 (-0.43--0.36) |
| **Oceania** | 0.03 (0.02-0.04) | 1.1 (0.9-1.3) | 0.07 (0.05-0.09) | 1.0 (0.8-1.3) | -0.28 (-0.31--0.25) |
| **South Asia** | 10.7 (8.9-15.2) | 2.0 (1.7-2.9) | 35.7 (28.0-40.9) | 2.6 (2.1-3.0) | 0.96 (0.81-1.11) |
| **Southeast Asia** | 4.5 (3.6-5.2) | 1.9 (1.5-2.2) | 11.4 (9.1-13.9) | 2.0 (1.6-2.4) | 0.18 (0.08-0.28) |
| **Southern Latin America** | 4.4 (3.5-4.7) | 9.6 (7.6-10.3) | 5.3 (4.1-7.1) | 6.3 (4.9-8.4) | -1.62 (-1.69--1.55) |
| **Southern Sub-Saharan Africa** | 0.25 (0.21-0.29) | 0.9 (0.8-1.1) | 0.49 (0.42-0.56) | 0.9 (0.8-1.0) | -0.04 (-0.26-0.18) |
| **Tropical Latin America** | 3.1 (2.6-3.3) | 3.6 (3.0-3.9) | 5.7 (4.9-6.6) | 2.4 (2.1-2.8) | -1.41 (-1.48--1.33) |
| **Western Europe** | 22.4 (17.7-23.7) | 3.8 (3.0-4.0) | 23.5 (19.3-27.4) | 2.4 (2.0-2.8) | -1.63 (-1.71--1.56) |
| **Western Sub-Saharan Africa** | 0.89 (0.73-1.15) | 1.1 (0.9-1.4) | 1.6 (1.3-2.4) | 1.0 (0.8-1.4) | -0.34 (-0.42--0.26) |

ASIR, age-standardized incidence rate; CI, confidence interval; EAPC, estimated annual percentage change; SDI, socio-demographic index; UI, uncertainty interval.

**Supplementary figure legends**

**Figure s1.** The trends of Chinese and the global number and age-standardized rates of deaths and DALYs of esophageal cancer from 1990 to 2019. (A) The total number of deaths and death rate per 100000 population; (B) The total number of DALYs and DALY rate per 100000 population. Shading indicates the upper and lower limits of the 95% UI. DALY, disability-adjusted life-year; UI, uncertainty interval.

**Figure s2.** The four clusters of 204 countries and territories integrating both EAPC of age-standardized incidence rate and death rate of esophageal cancer. EAPC, estimated annual percentage change.

**Figure s3.** Proportion of esophageal cancer deaths and DALYs attributable to different risk factors in 1990 and 2019. (A) 1990; (B) 2019. BMI, body mass index; DALY, disability-adjusted life-year; SDI, socio-demographic index.

**Figure s4.** The trends of Chinese and the global number and age-standardized rates of deaths and DALYs of stomach cancer from 1990 to 2019. (A) The total number of deaths and death rate per 100000 population; (B) The total number of DALYs and DALY rate per 100000 population. Shading indicates the upper and lower limits of the 95% UI. DALY, disability-adjusted life-year; UI, uncertainty interval.

**Figure s5.** The four clusters of 204 countries and territories integrating both EAPC of age-standardized incidence rate and death rate of stomach cancer. EAPC, estimated annual percentage change.

**Figure s6.** Proportion of stomach cancer deaths and DALYs attributable to different risk factors in 1990 and 2019. (A) 1990; (B) 2019. DALY, disability-adjusted life-year; SDI, socio-demographic index.

**Figure s7.** The trends of Chinese and the global number and age-standardized rates of deaths and DALYs of liver cancer from 1990 to 2019. (A) The total number of deaths and death rate per 100000 population; (B) The total number of DALYs and DALY rate per 100000 population. Shading indicates the upper and lower limits of the 95% UI. DALY, disability-adjusted life-year; UI, uncertainty interval.

**Figure s8.** The four clusters of 204 countries and territories integrating both EAPC of age-standardized incidence rate and death rate of liver cancer. EAPC, estimated annual percentage change.

**Figure s9.** Proportion of liver cancer deaths and DALYs attributable to different risk factors in 1990 and 2019. (A) 1990; (B) 2019. BMI, body mass index; DALY, disability-adjusted life-year; SDI, socio-demographic index.

**Figure s10.** The trends of Chinese and the global number and age-standardized rates of deaths and DALYs of pancreatic cancer from 1990 to 2019. (A) The total number of deaths and death rate per 100000 population; (B) The total number of DALYs and DALY rate per 100000 population. Shading indicates the upper and lower limits of the 95% UI. DALY, disability-adjusted life-year; UI, uncertainty interval.

**Figure s11.** The four clusters of 204 countries and territories integrating both EAPC of age-standardized incidence rate and death rate of pancreatic cancer. EAPC, estimated annual percentage change.

**Figure s12.** Proportion of pancreatic cancer deaths and DALYs attributable to different risk factors in 1990 and 2019. (A) 1990; (B) 2019. BMI, body mass index; DALY, disability-adjusted life-year; SDI, socio-demographic index.

**Figure s13.** The trends of Chinese and the global number and age-standardized rates of deaths and DALYs of colon and rectum cancer from 1990 to 2019. (A) The total number of deaths and death rate per 100000 population; (B) The total number of DALYs and DALY rate per 100000 population. Shading indicates the upper and lower limits of the 95% UI. DALY, disability-adjusted life-year; UI, uncertainty interval.

**Figure s14.** The four clusters of 204 countries and territories integrating both EAPC of age-standardized incidence rate and death rate of colon and rectum cancer. EAPC, estimated annual percentage change.

**Figure s15.** Proportion of colon and rectum cancer deaths and DALYs attributable to different risk factors in 1990 and 2019. (A) 1990; (B) 2019. BMI, body mass index; DALY, disability-adjusted life-year; SDI, socio-demographic index.

**Figure s16.** The trends of Chinese and the global number and age-standardized rates of deaths and DALYs of gallbladder and biliary tract cancer from 1990 to 2019. (A) The total number of deaths and death rate per 100000 population; (B) The total number of DALYs and DALY rate per 100000 population. Shading indicates the upper and lower limits of the 95% UI. DALY, disability-adjusted life-year; UI, uncertainty interval.

**Figure s17.** The four clusters of 204 countries and territories integrating both EAPC of age-standardized incidence rate and death rate of gallbladder and biliary tract cancer. EAPC, estimated annual percentage change.

**Figure s18.** Proportion of gallbladder and biliary tract cancer deaths and DALYs attributable to high BMI in 1990 and 2019. (A) 1990; (B) 2019. BMI, body mass index; DALY, disability-adjusted life-year; SDI, socio-demographic index.

**Figure s19.** Contribution of the incidence cases and death cases of six gastrointestinal cancers in China and globally in 1990 and 2019. (A) Incidence; (B) Death. SDI, socio-demographic index.

**Figure s20.** The proportion of incidence and death cases of six gastrointestinal cancers in China among global cases in 1990 and 2019. (A) Incidence; (B) Death.
