# Supplementary figures and images for: Chinese and Global Burdens of Gastrointestinal Cancers From 1990 to 2019
Source: Front Public Health. 2022 Jul 13;10:941284. doi: 10.3389/fpubh.2022.941284 (PMC9326121; doi:10.3389/fpubh.2022.941284)

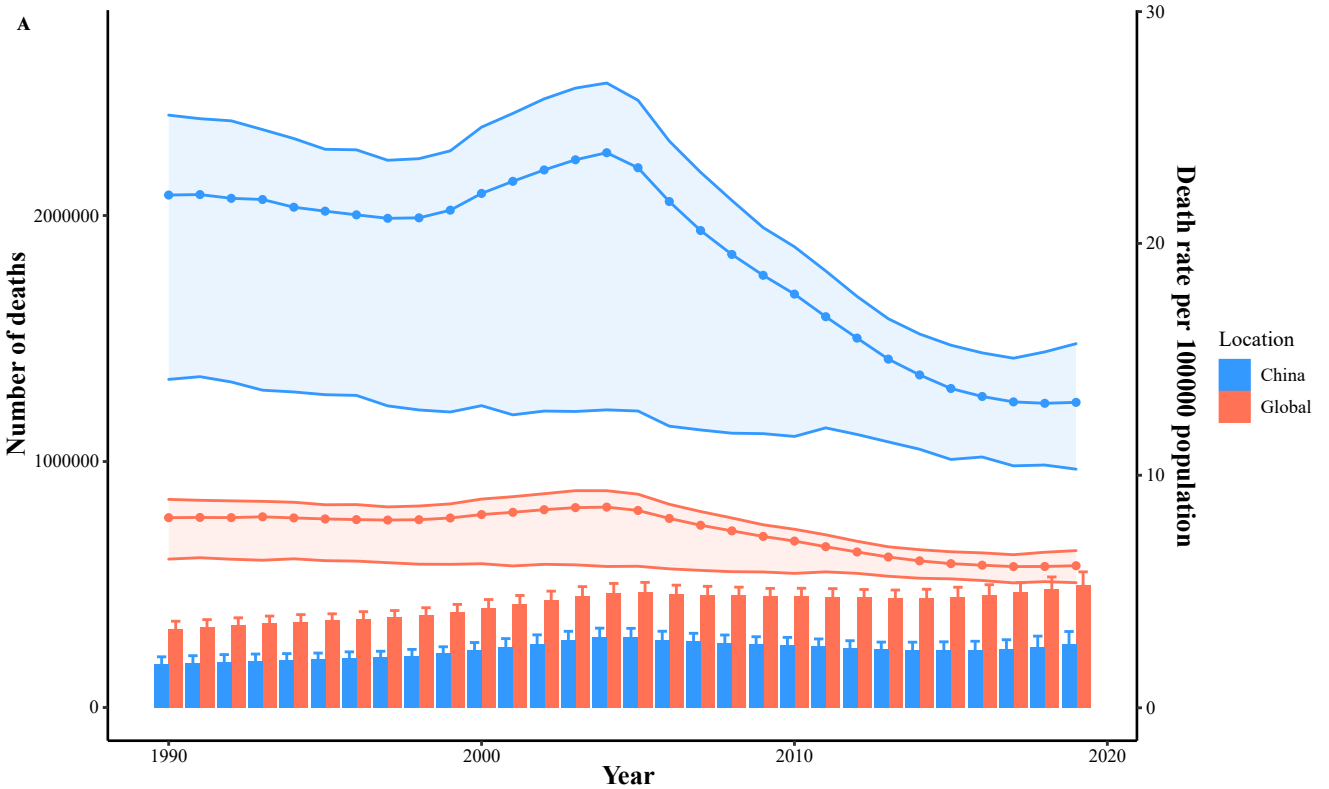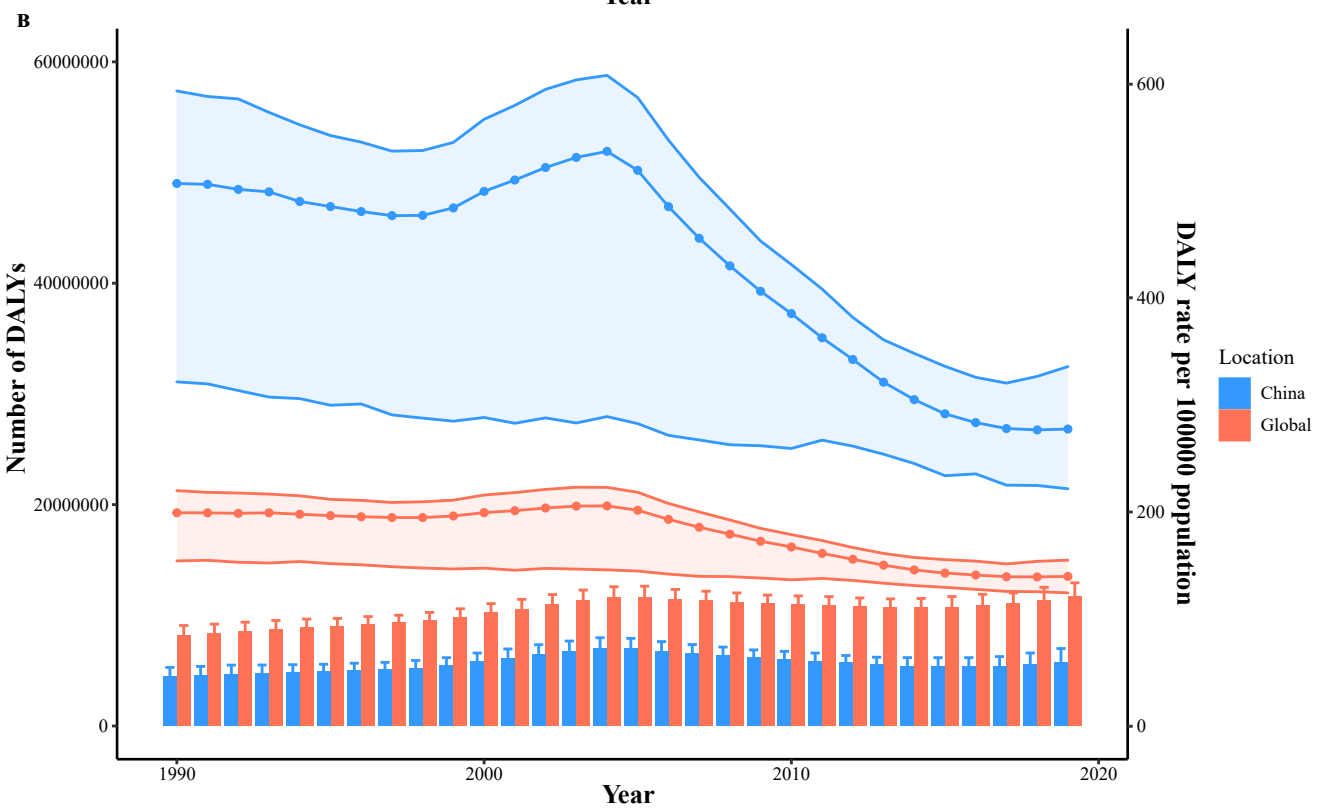

Supplement: Supplementary file 1 [file Data_Sheet_1.ZIP › Supplementary Material/Fig. s1.pdf]

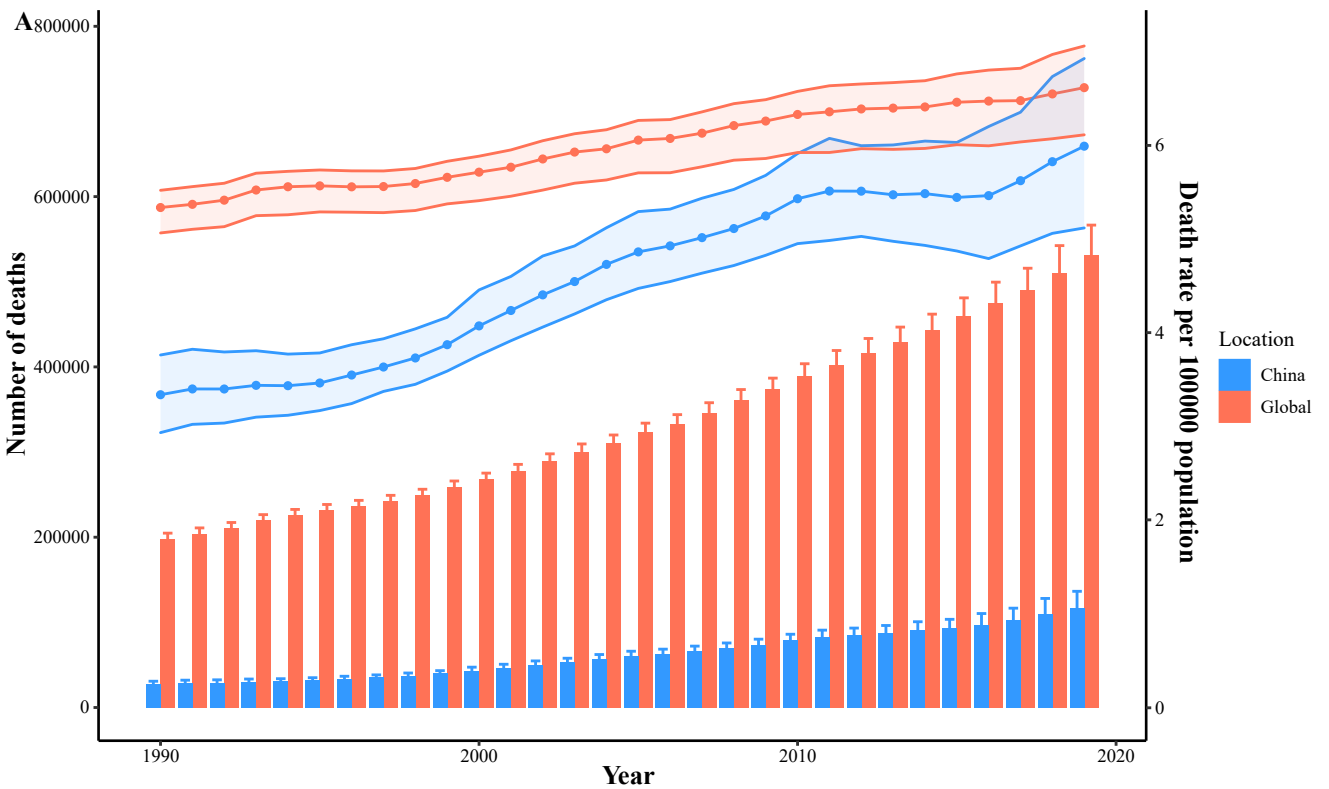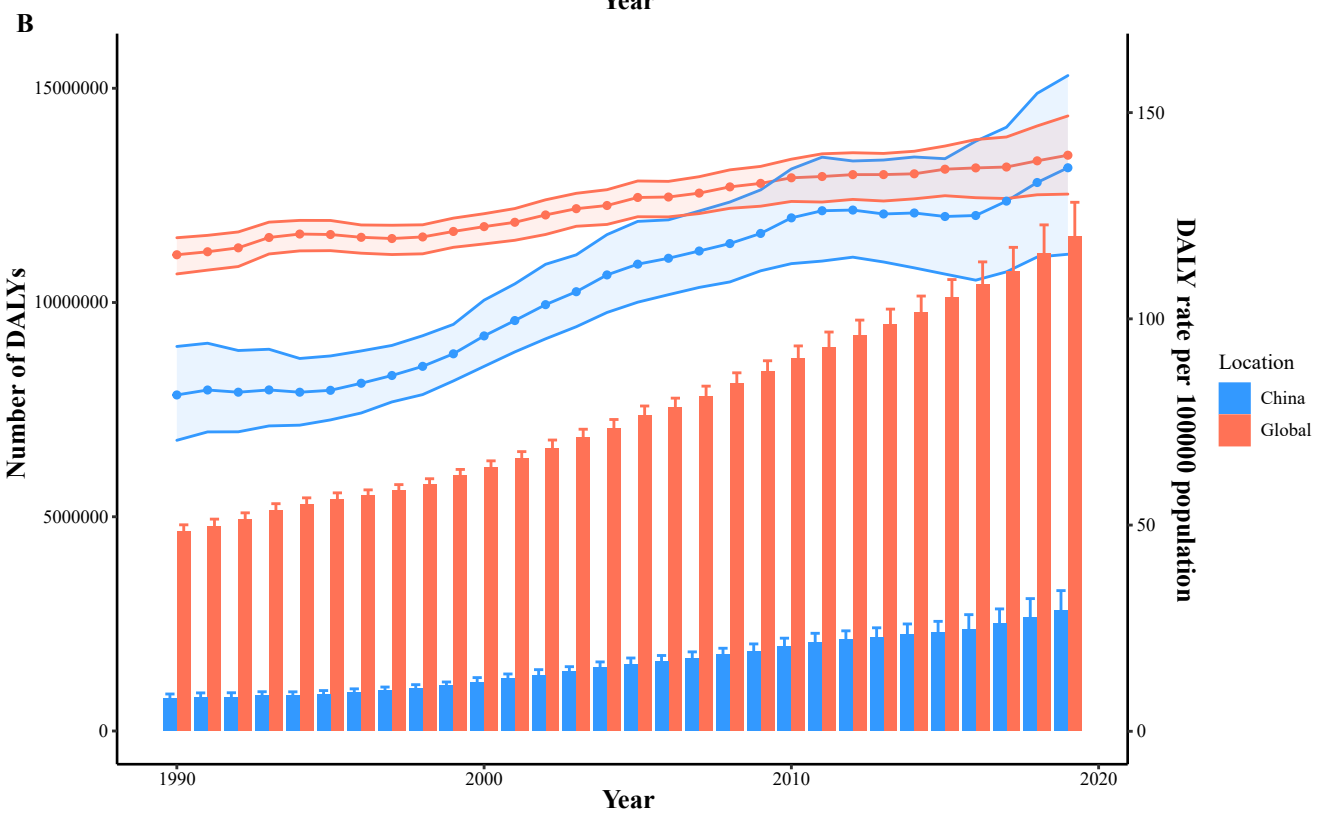

Supplement: Supplementary file 1 [file Data_Sheet_1.ZIP › Supplementary Material/Fig. s10.pdf]

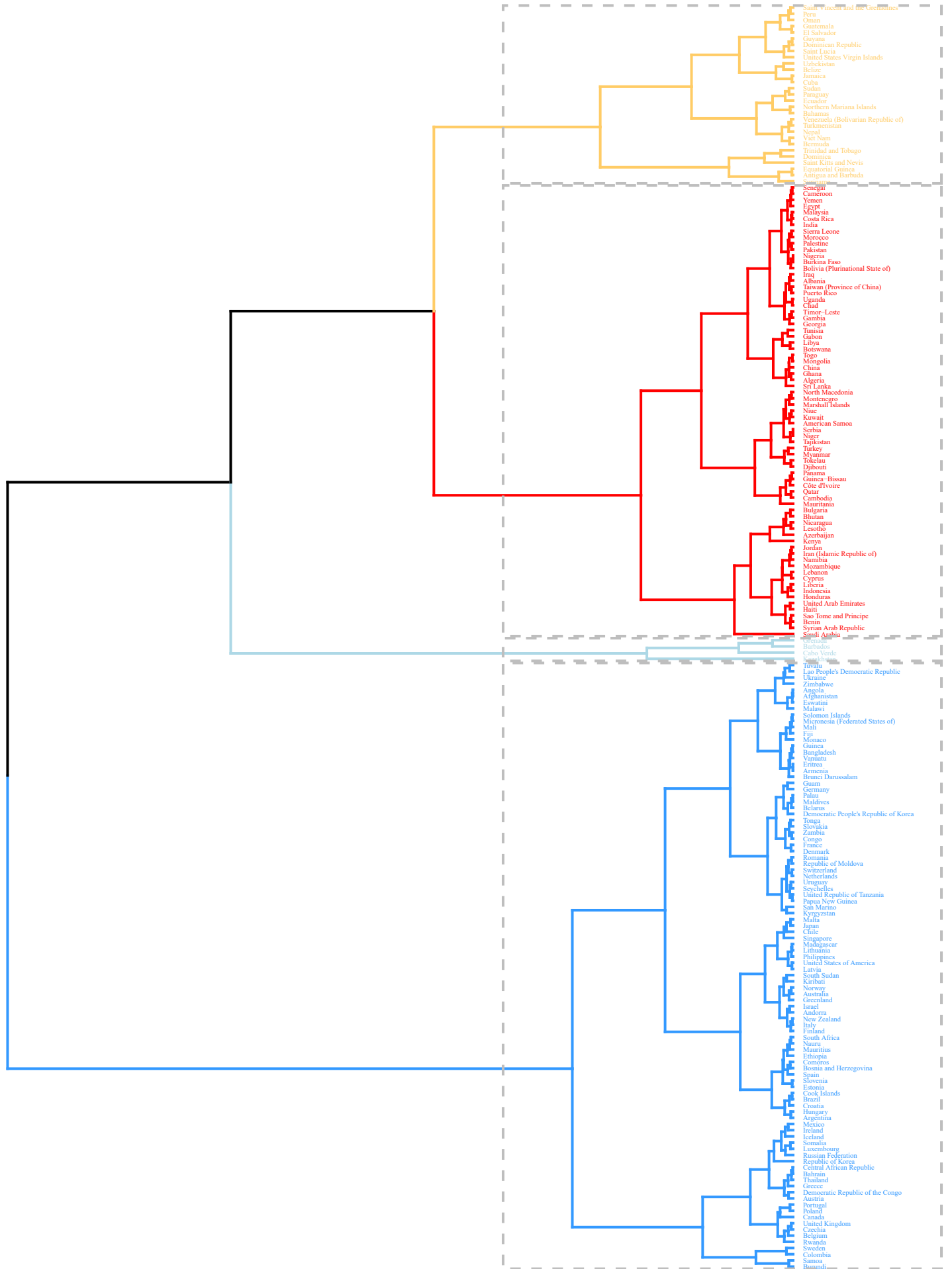

Supplement: Supplementary file 1 [file Data_Sheet_1.ZIP › Supplementary Material/Fig. s11.pdf]

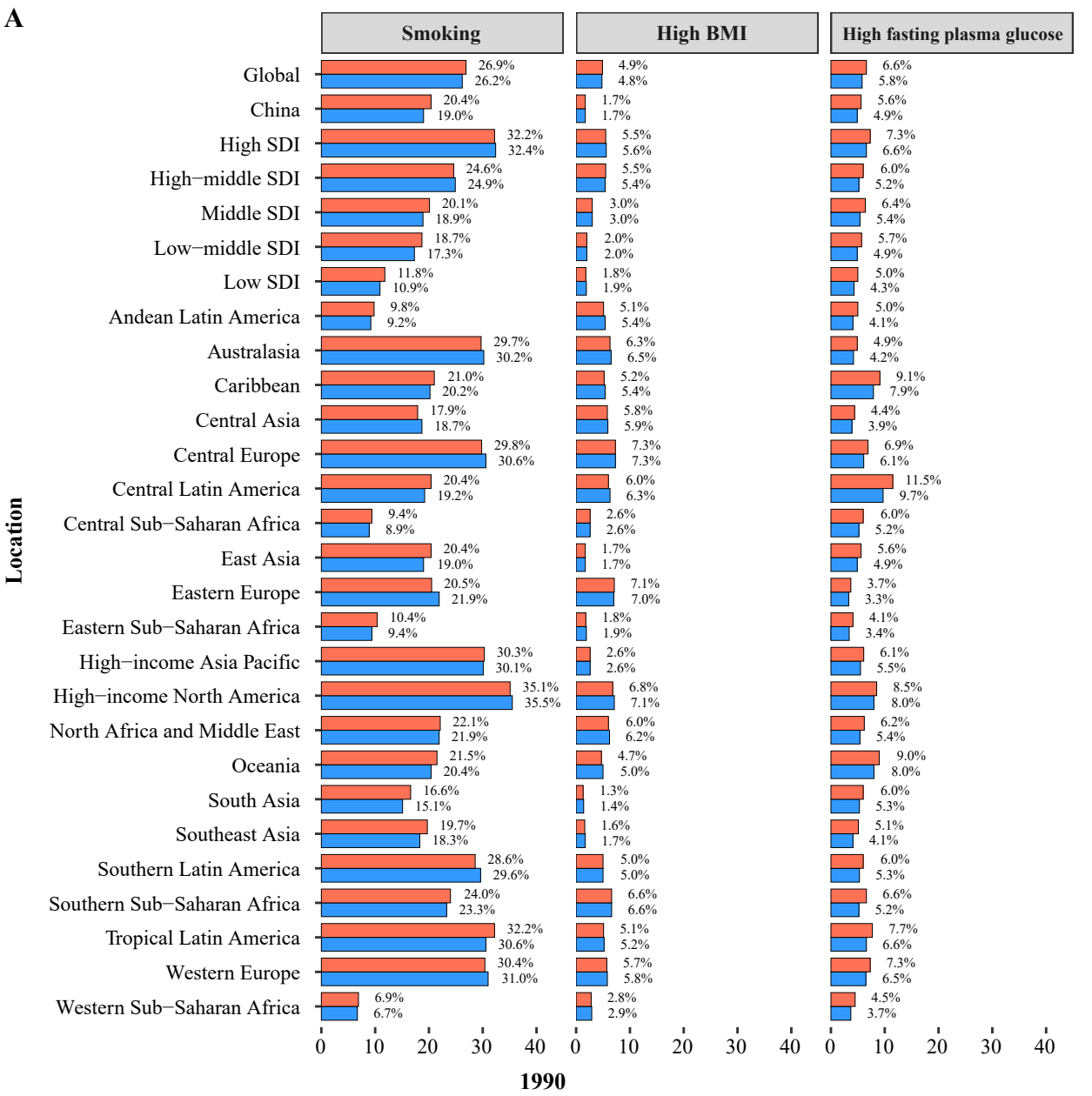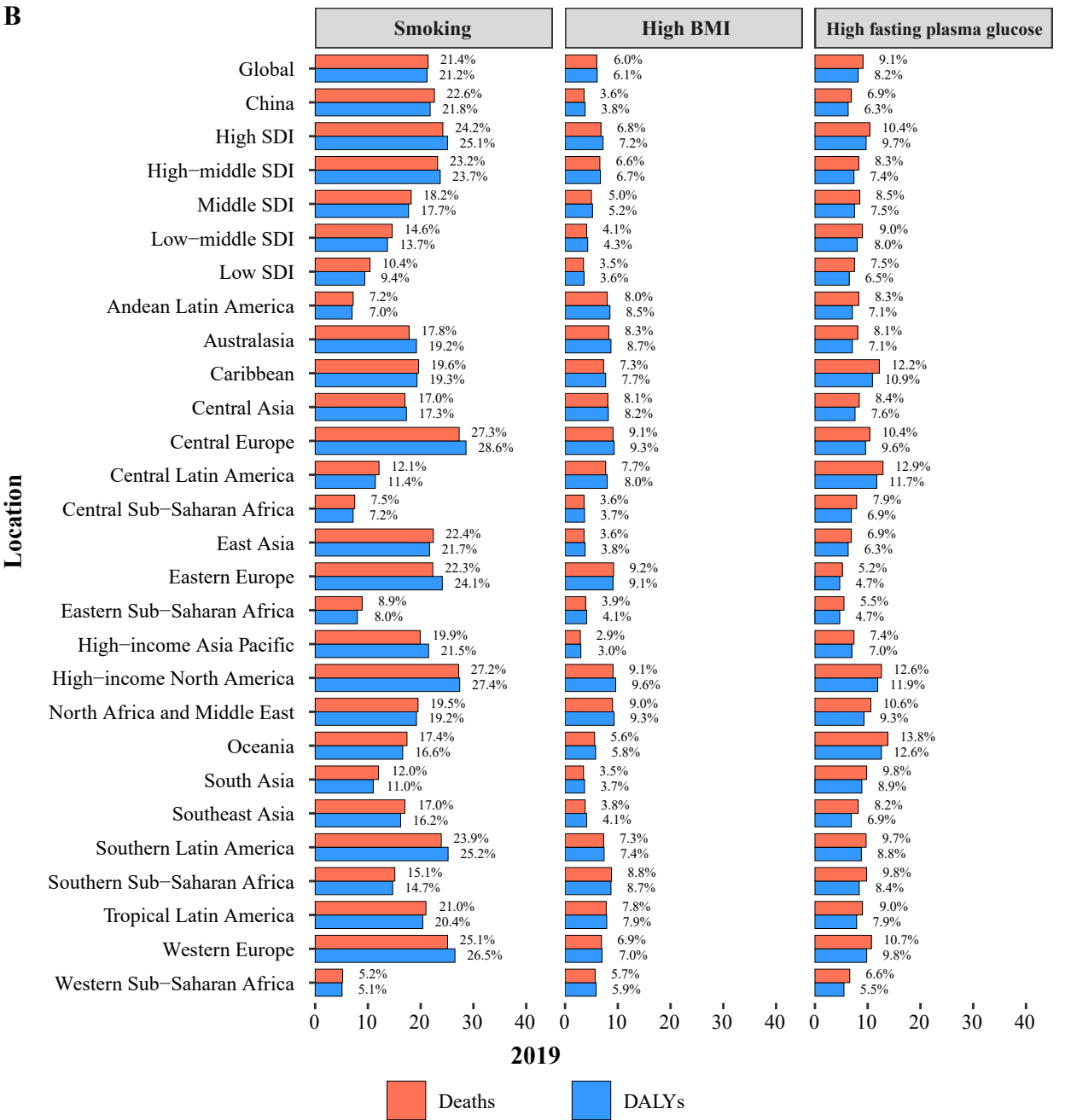

Supplement: Supplementary file 1 [file Data_Sheet_1.ZIP › Supplementary Material/Fig. s12.pdf]

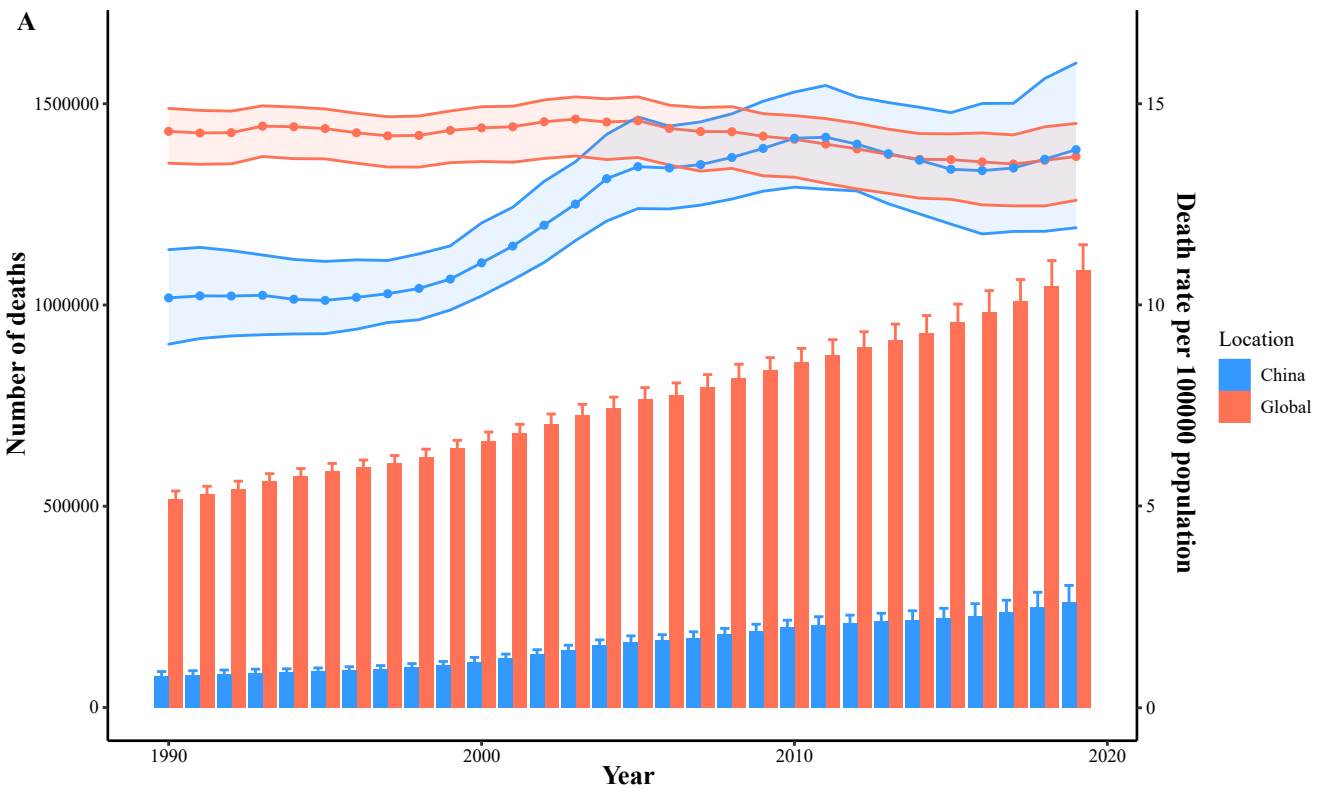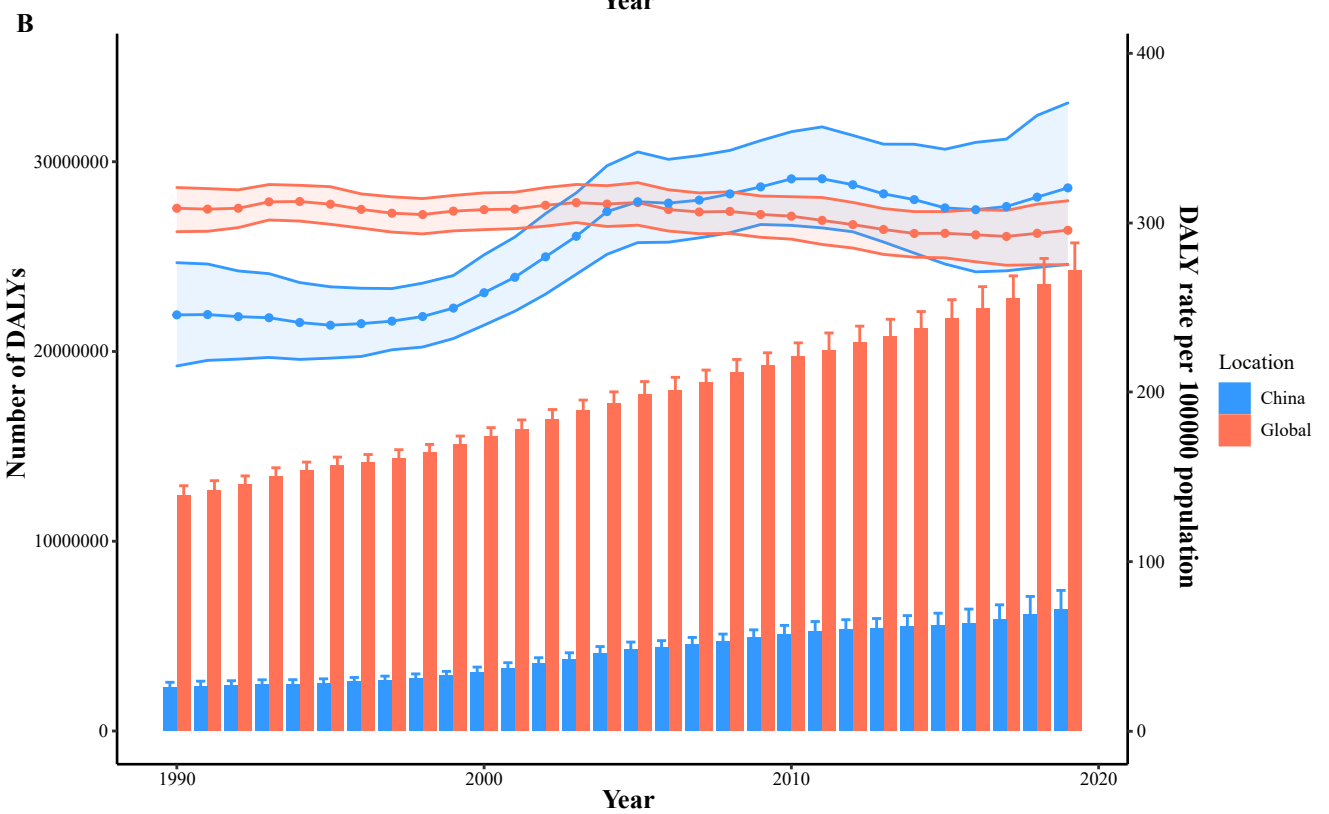

Supplement: Supplementary file 1 [file Data_Sheet_1.ZIP › Supplementary Material/Fig. s13.pdf]

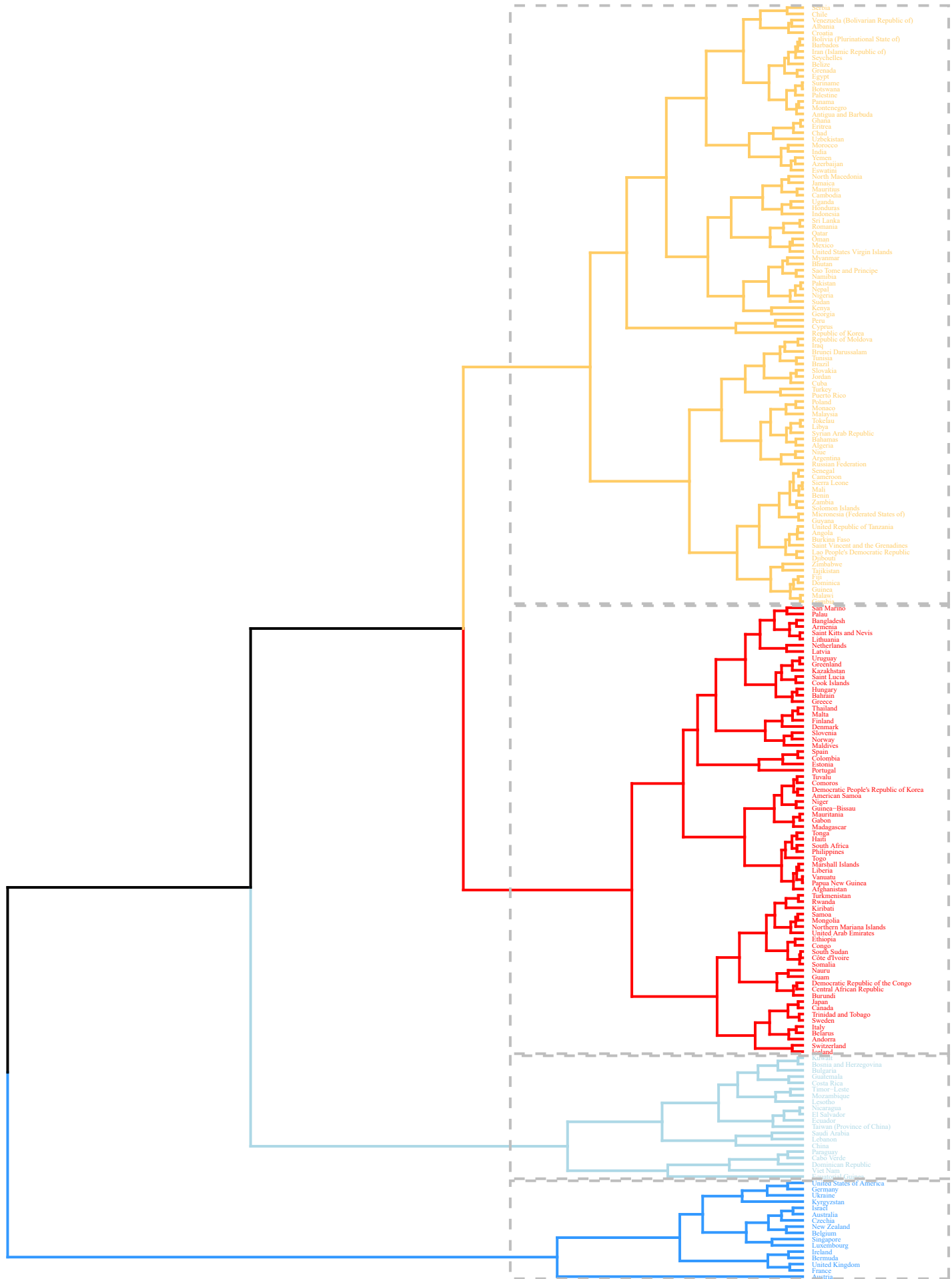

Supplement: Supplementary file 1 [file Data_Sheet_1.ZIP › Supplementary Material/Fig. s14.pdf]

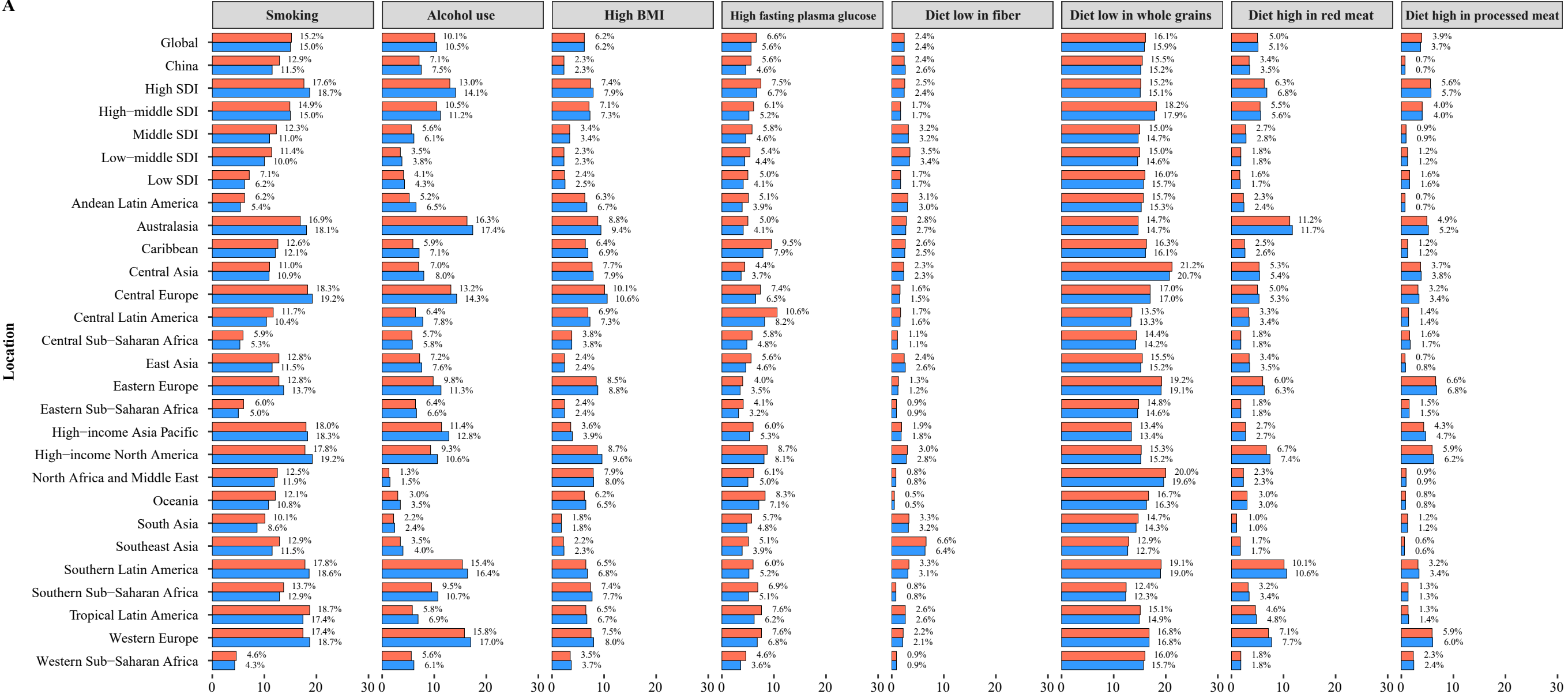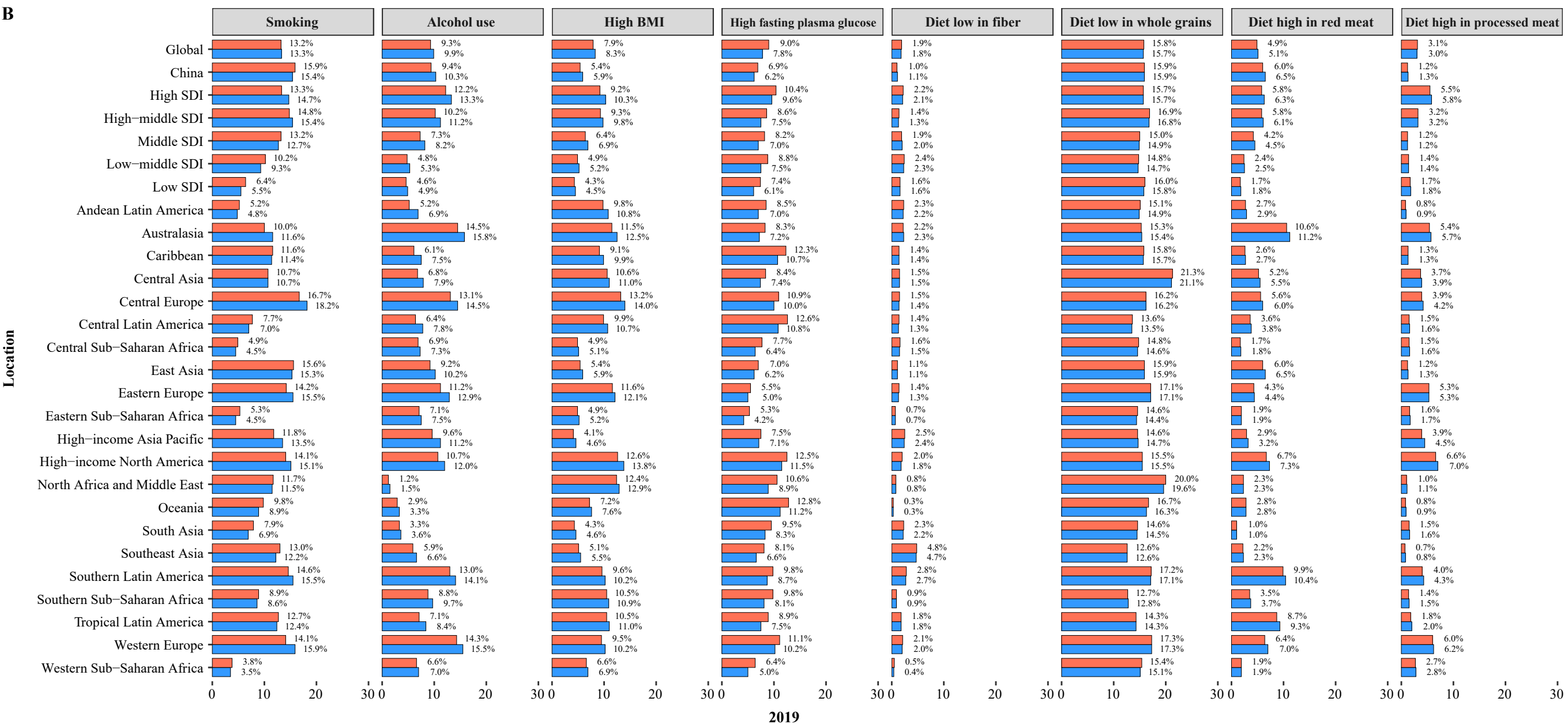

Deaths DALYs

Supplement: Supplementary file 1 [file Data_Sheet_1.ZIP › Supplementary Material/Fig. s15.pdf]

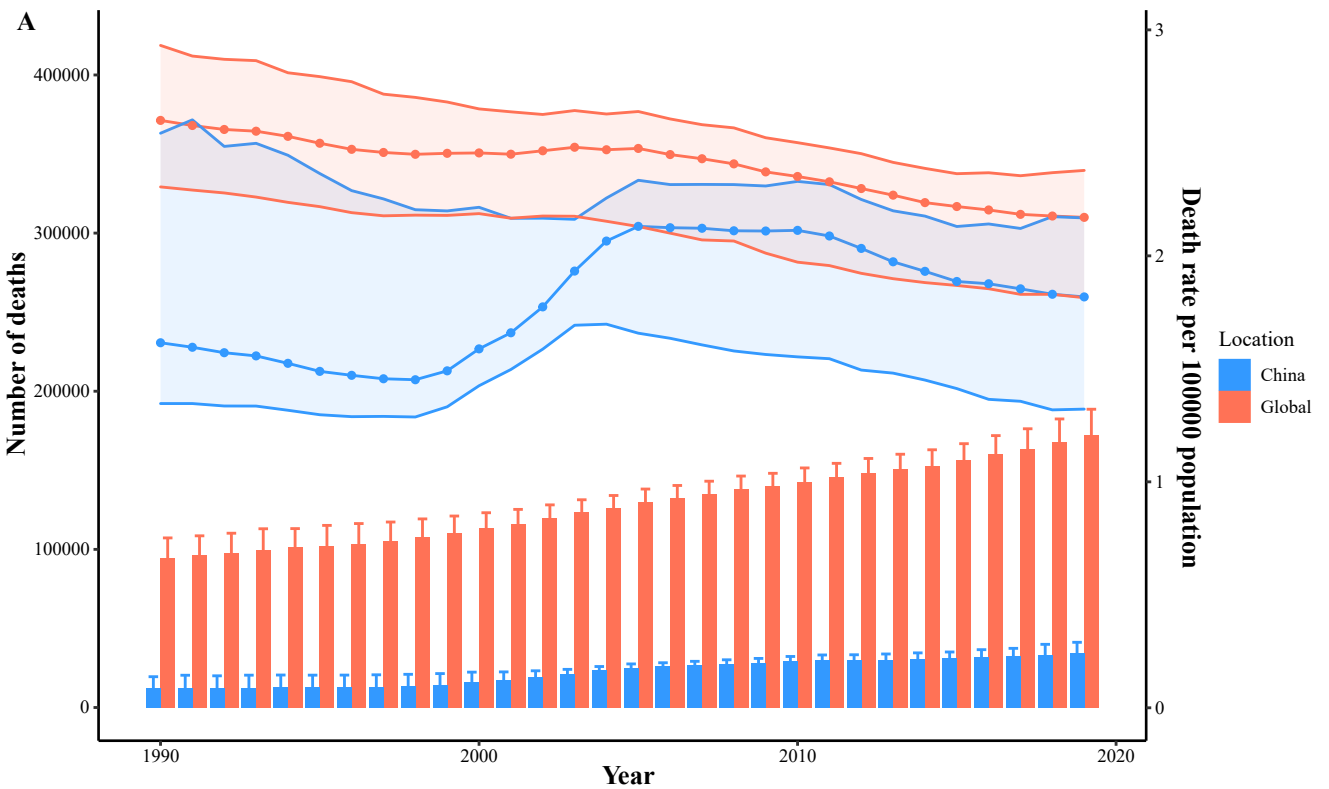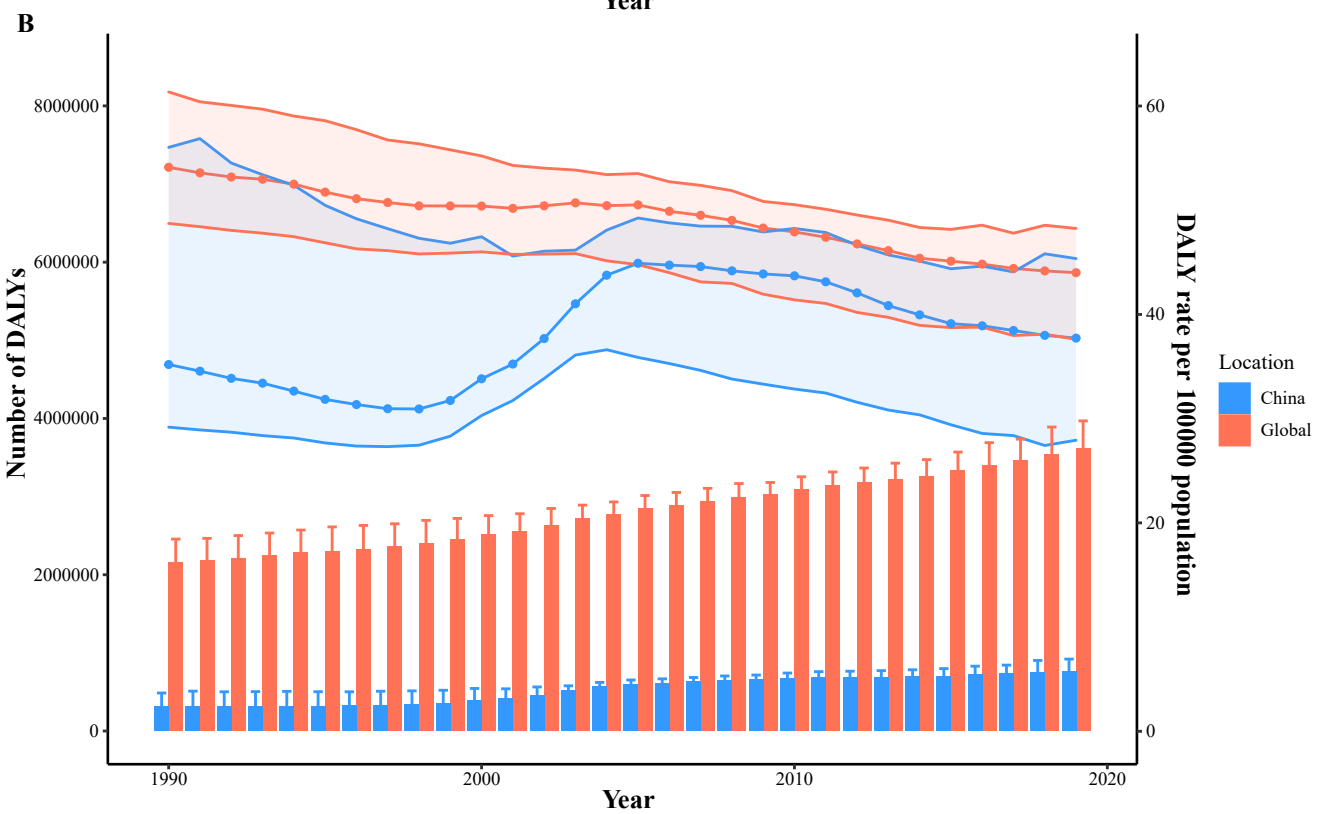

Supplement: Supplementary file 1 [file Data_Sheet_1.ZIP › Supplementary Material/Fig. s16.pdf]

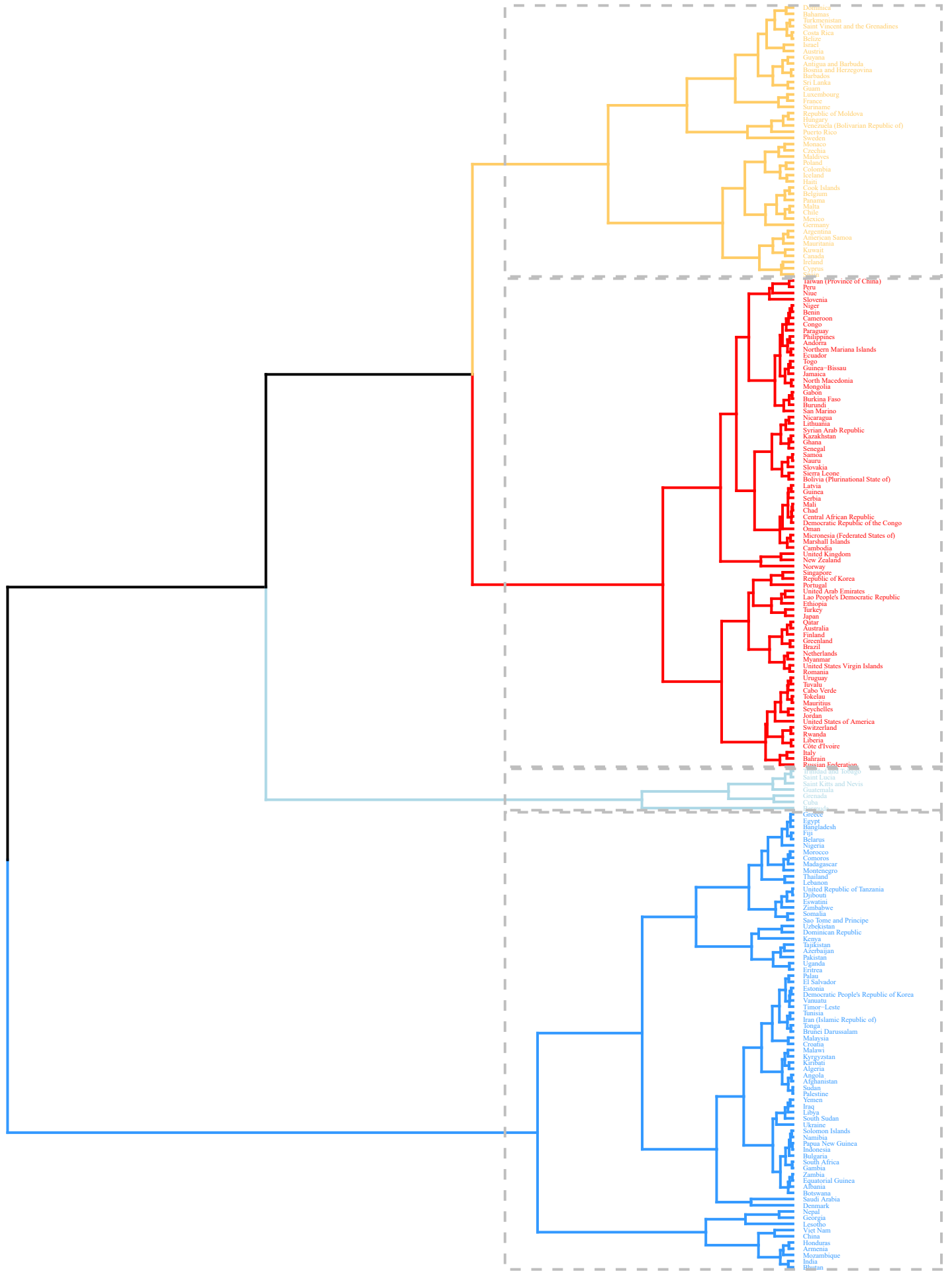

Supplement: Supplementary file 1 [file Data_Sheet_1.ZIP › Supplementary Material/Fig. s17.pdf]

A

Location

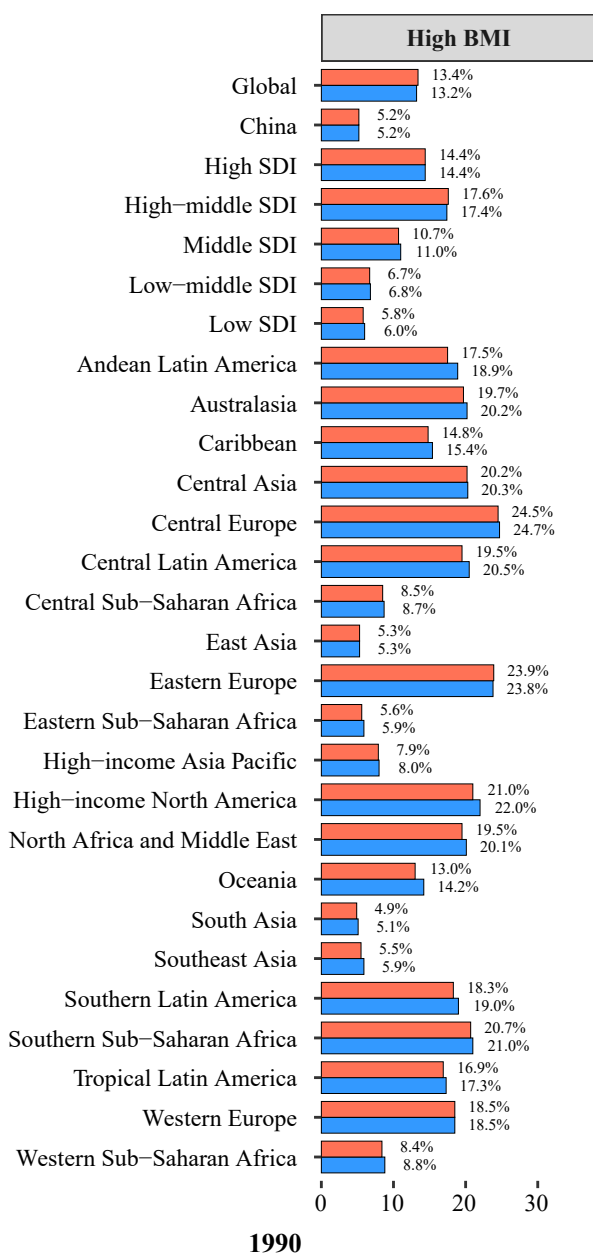

Deaths

B

Location

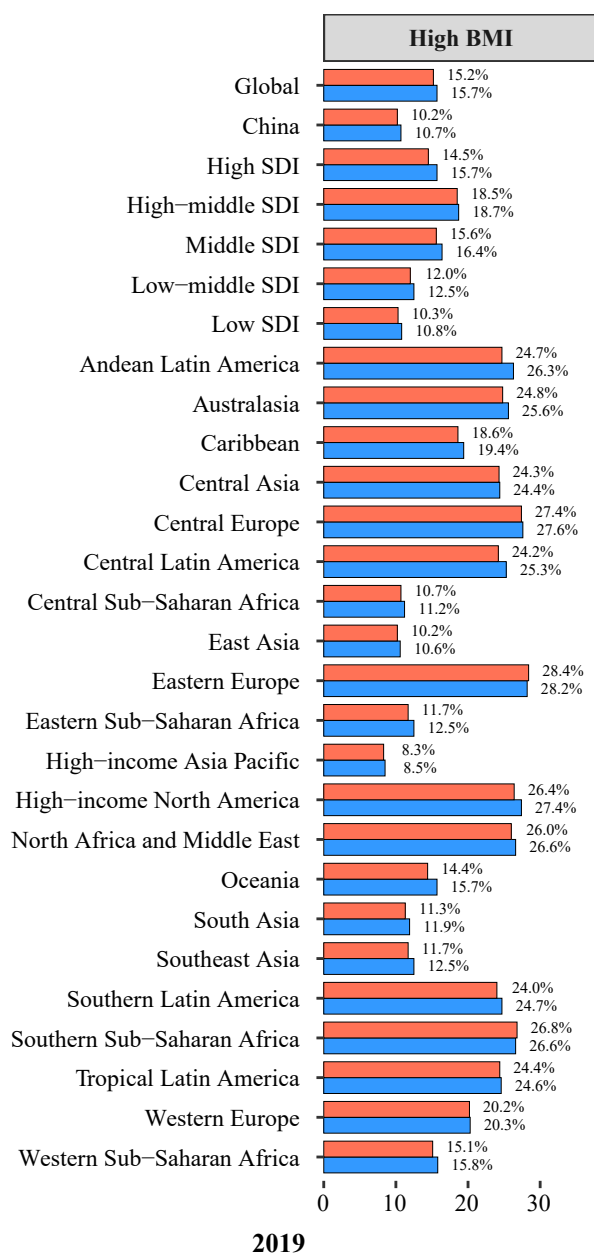

DALYs

Supplement: Supplementary file 1 [file Data_Sheet_1.ZIP › Supplementary Material/Fig. s18.pdf]

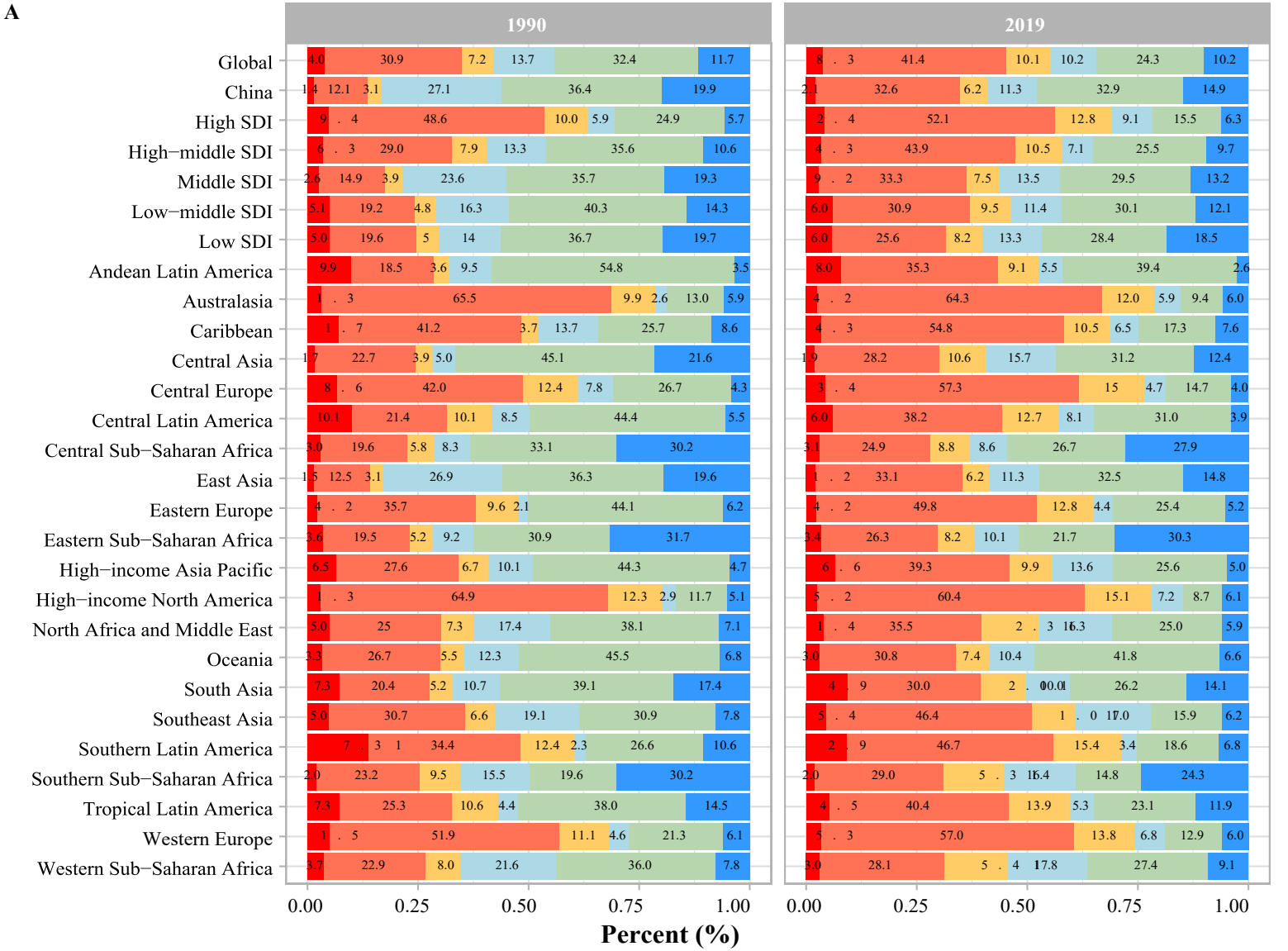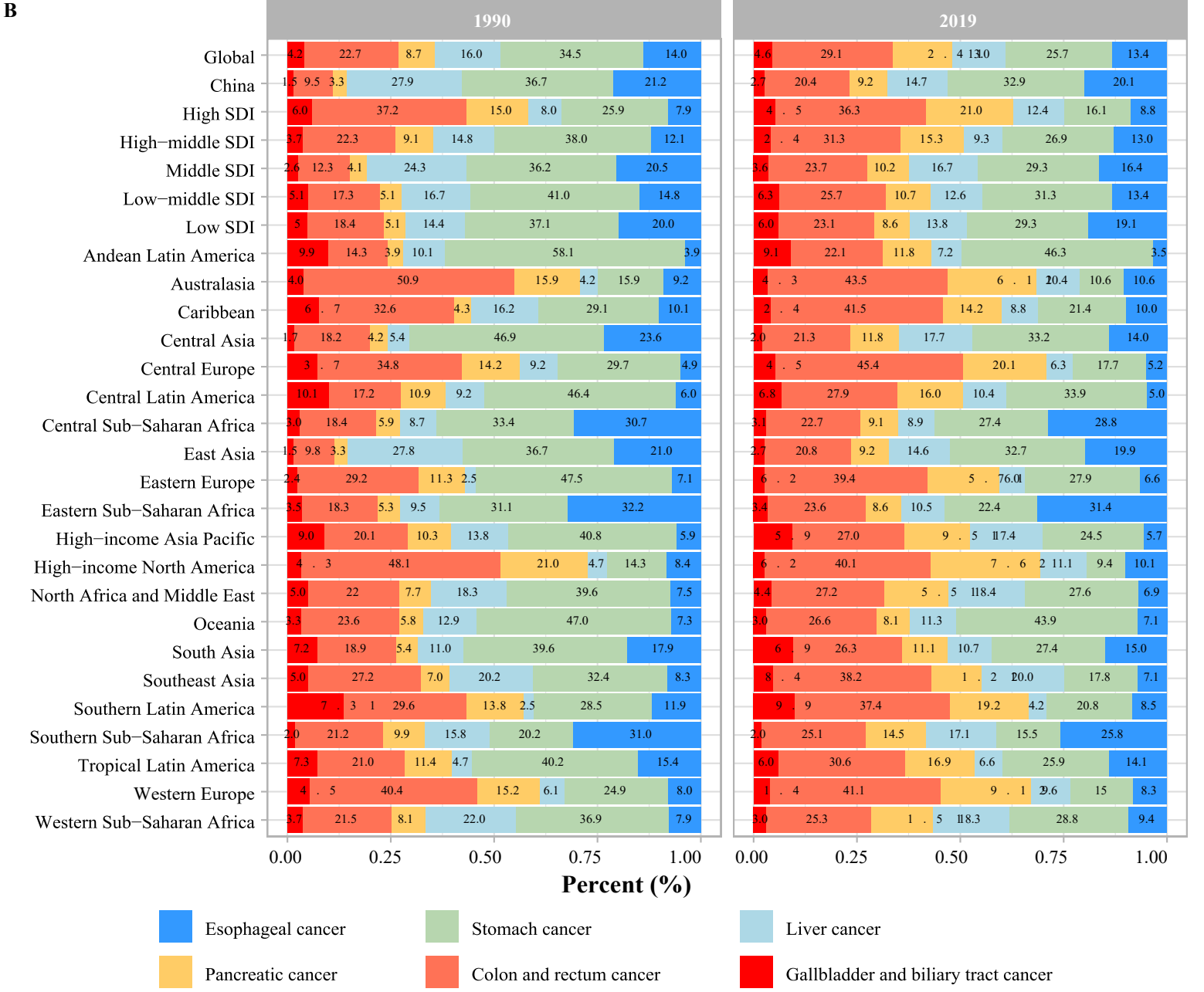

Supplement: Supplementary file 1 [file Data_Sheet_1.ZIP › Supplementary Material/Fig. s19.pdf]

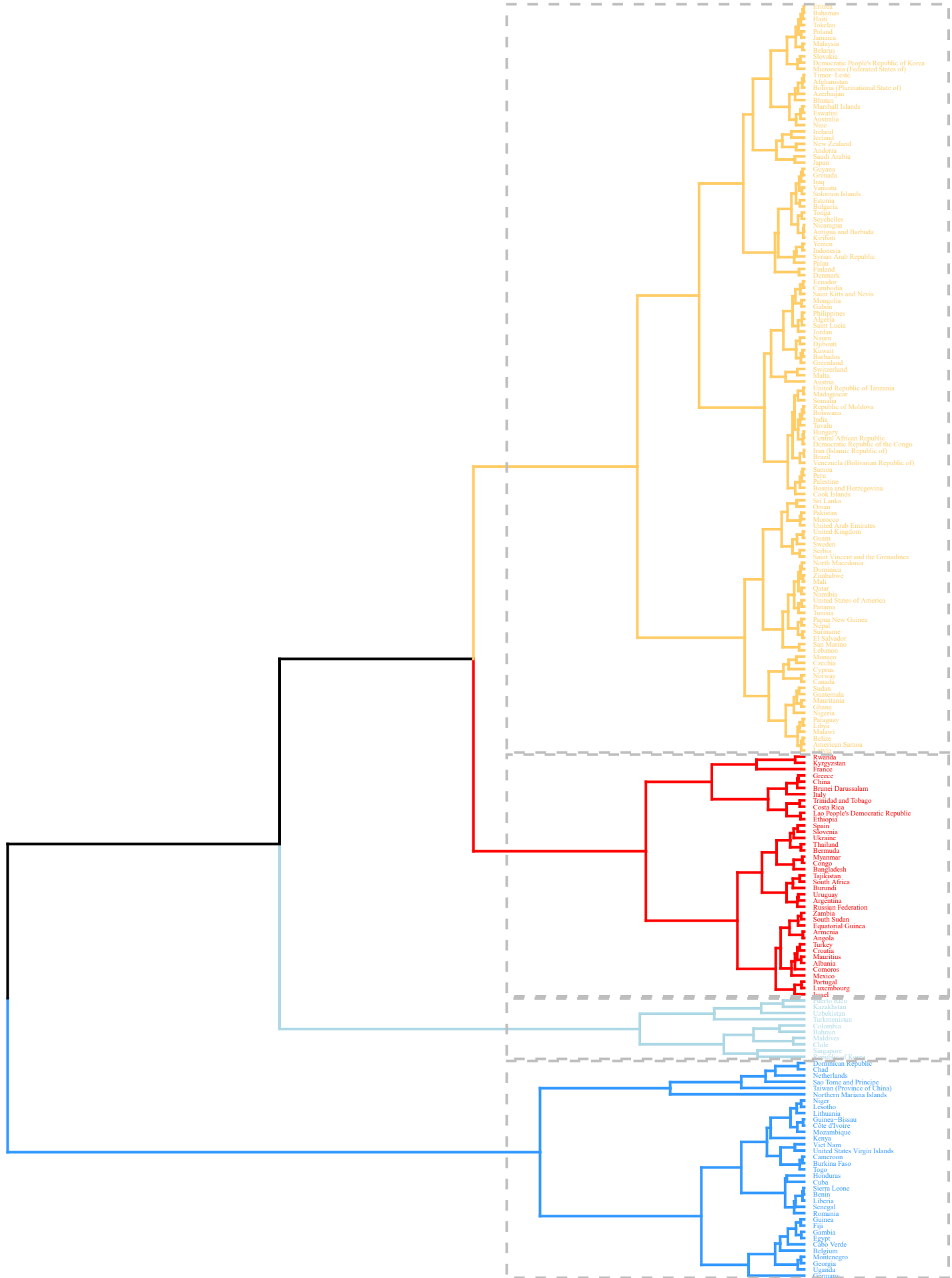

Supplement: Supplementary file 1 [file Data_Sheet_1.ZIP › Supplementary Material/Fig. s2.pdf]

**A**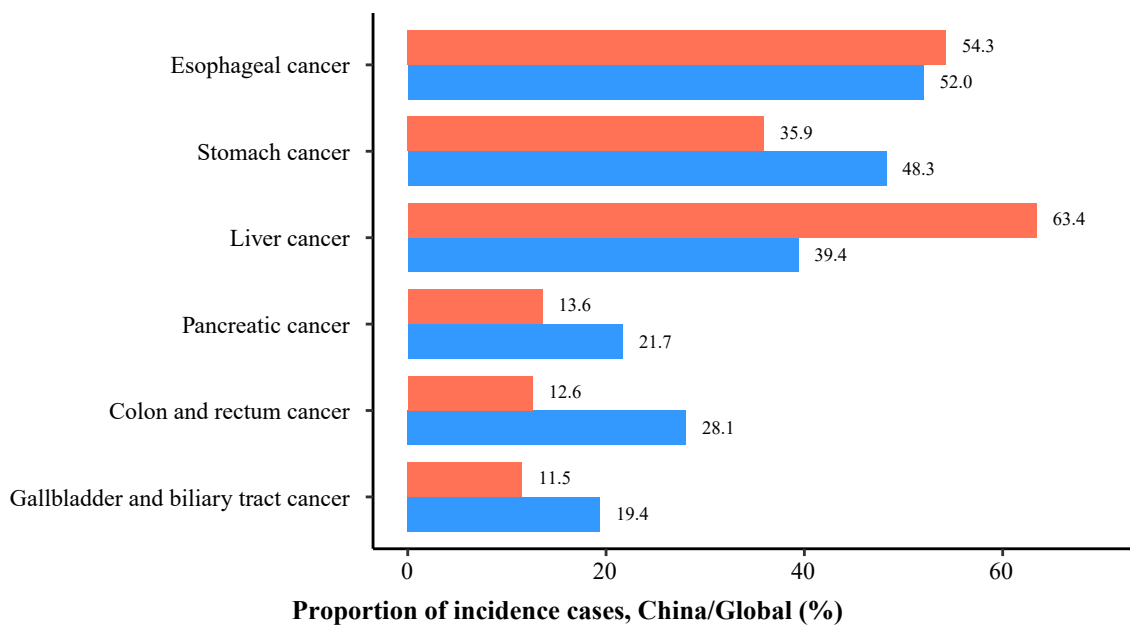**B**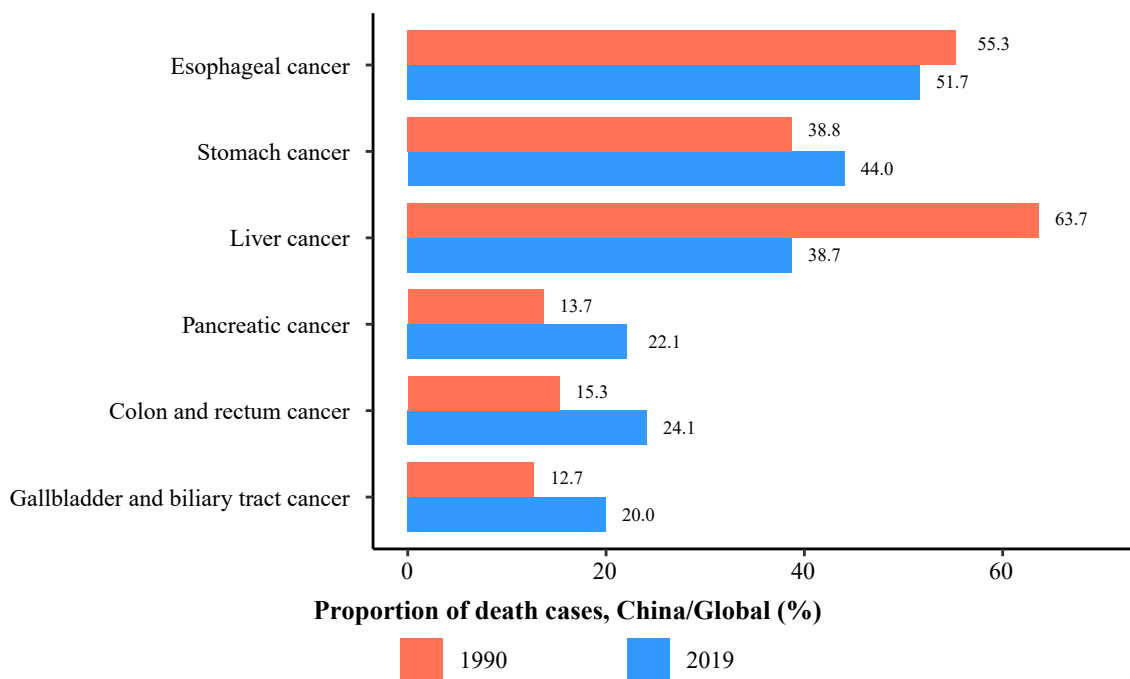

Supplement: Supplementary file 1 [file Data_Sheet_1.ZIP › Supplementary Material/Fig. s20.pdf]

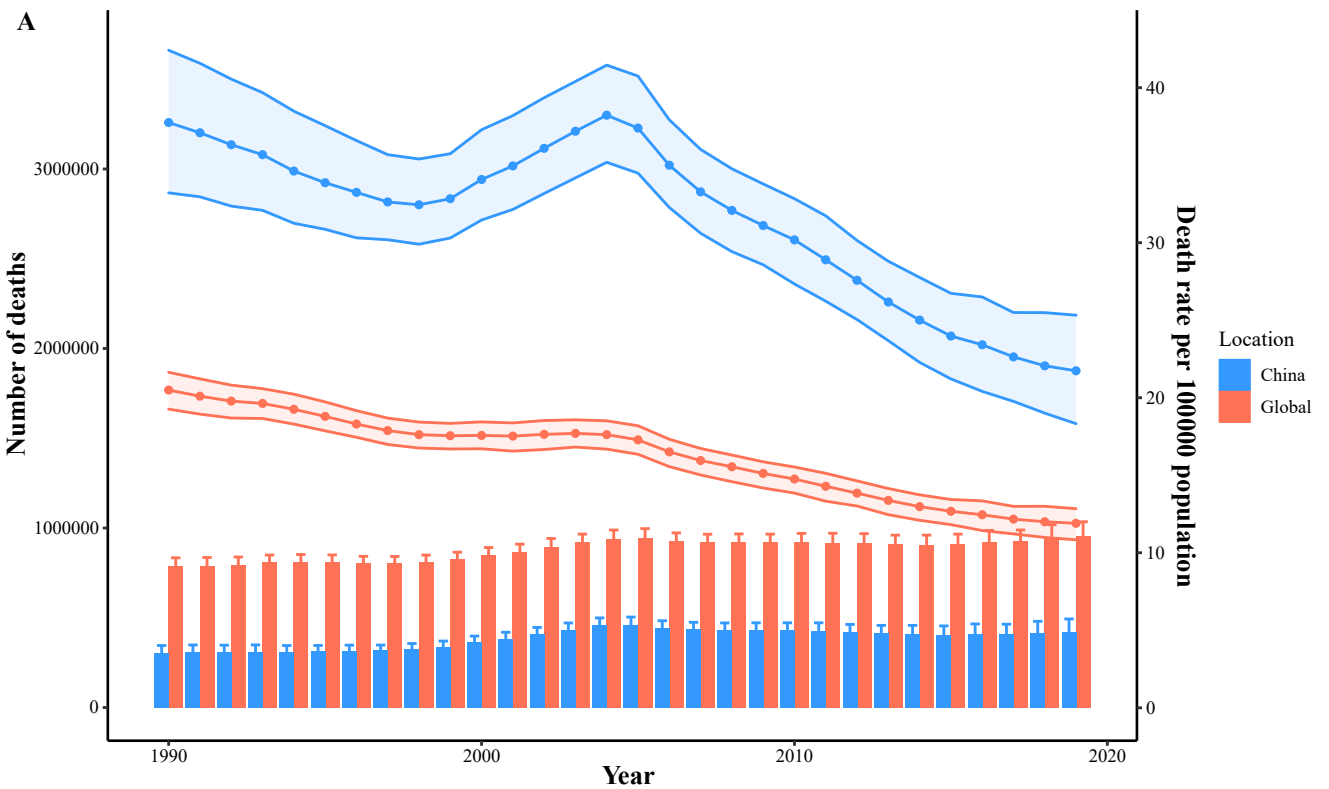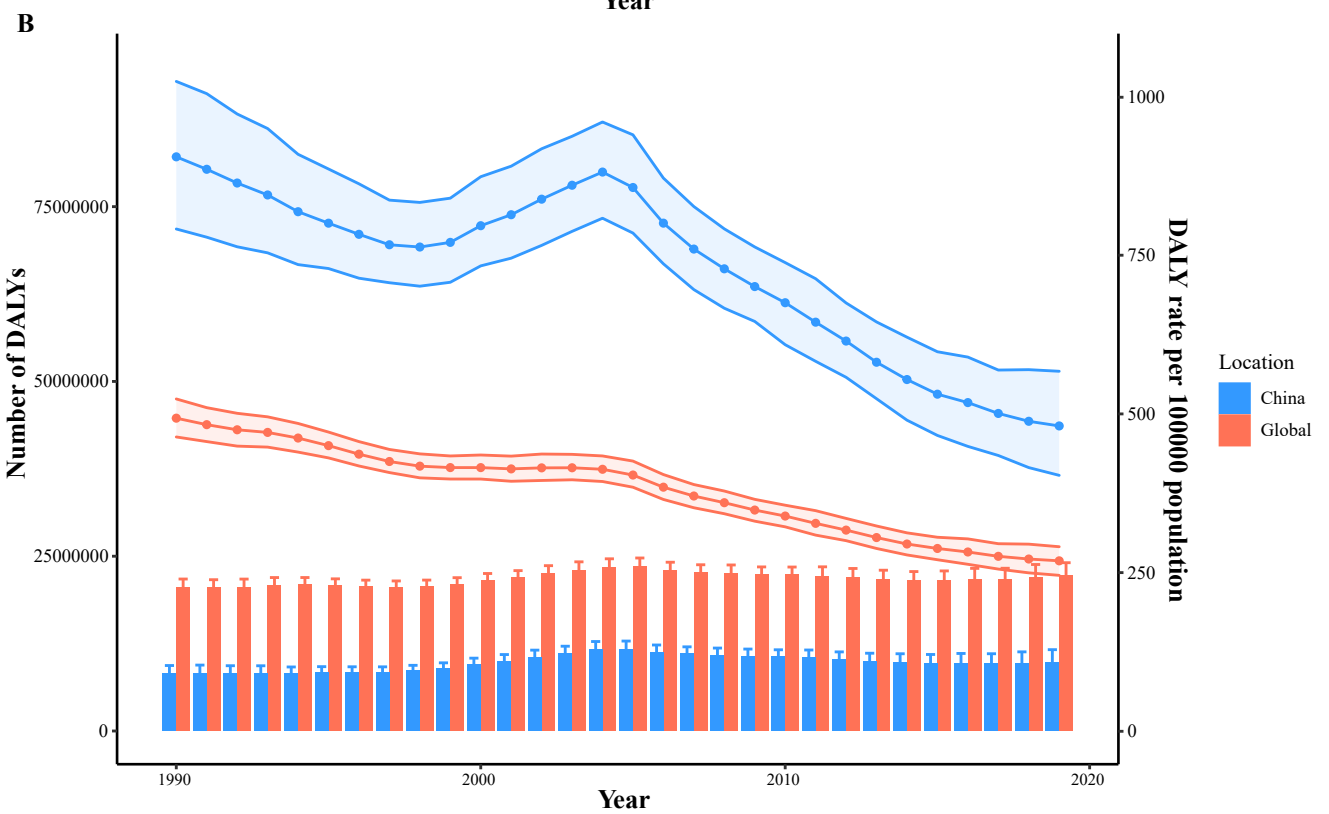

Supplement: Supplementary file 1 [file Data_Sheet_1.ZIP › Supplementary Material/Fig. s4.pdf]

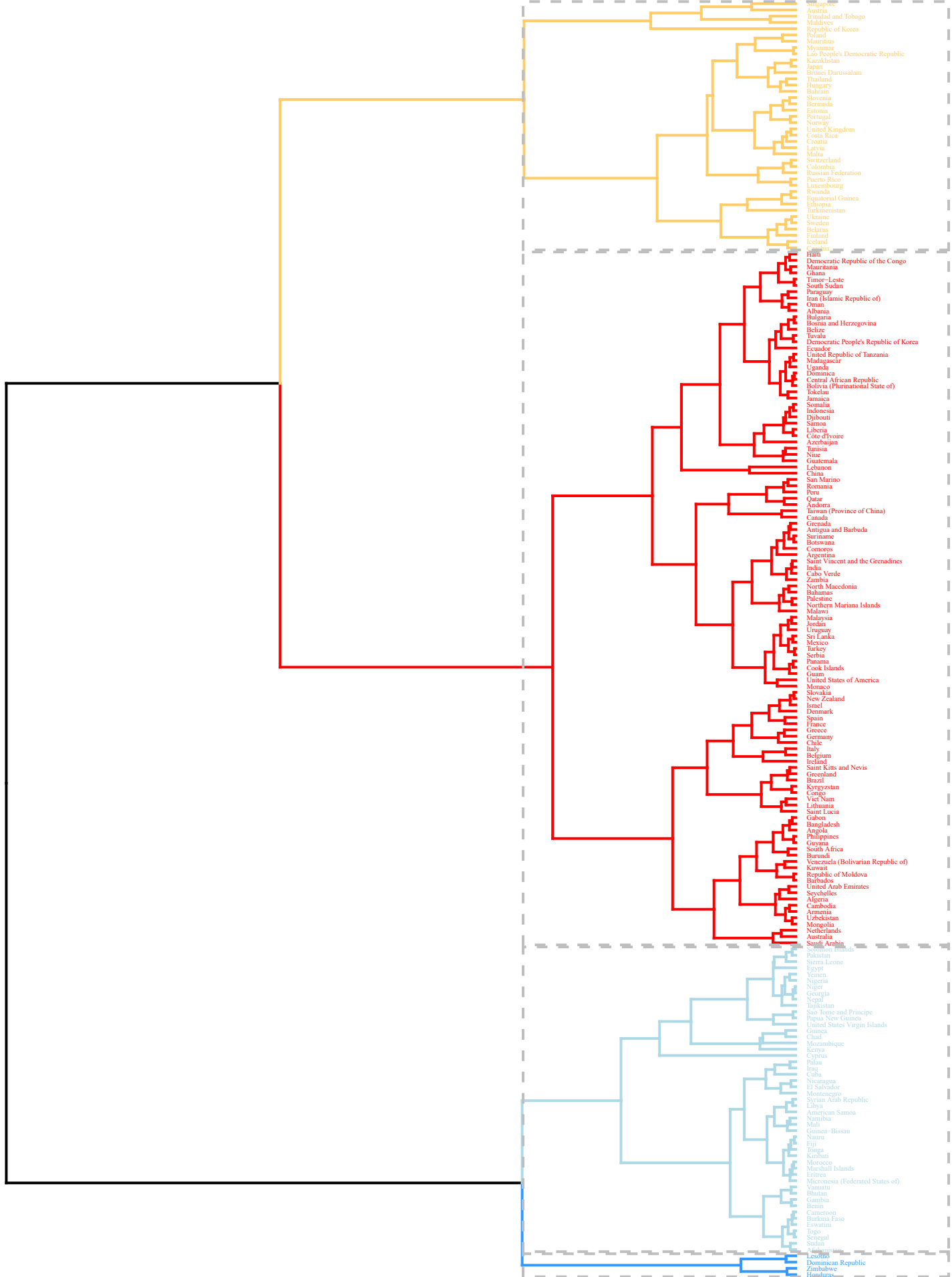

Supplement: Supplementary file 1 [file Data_Sheet_1.ZIP › Supplementary Material/Fig. s5.pdf]

A

Location

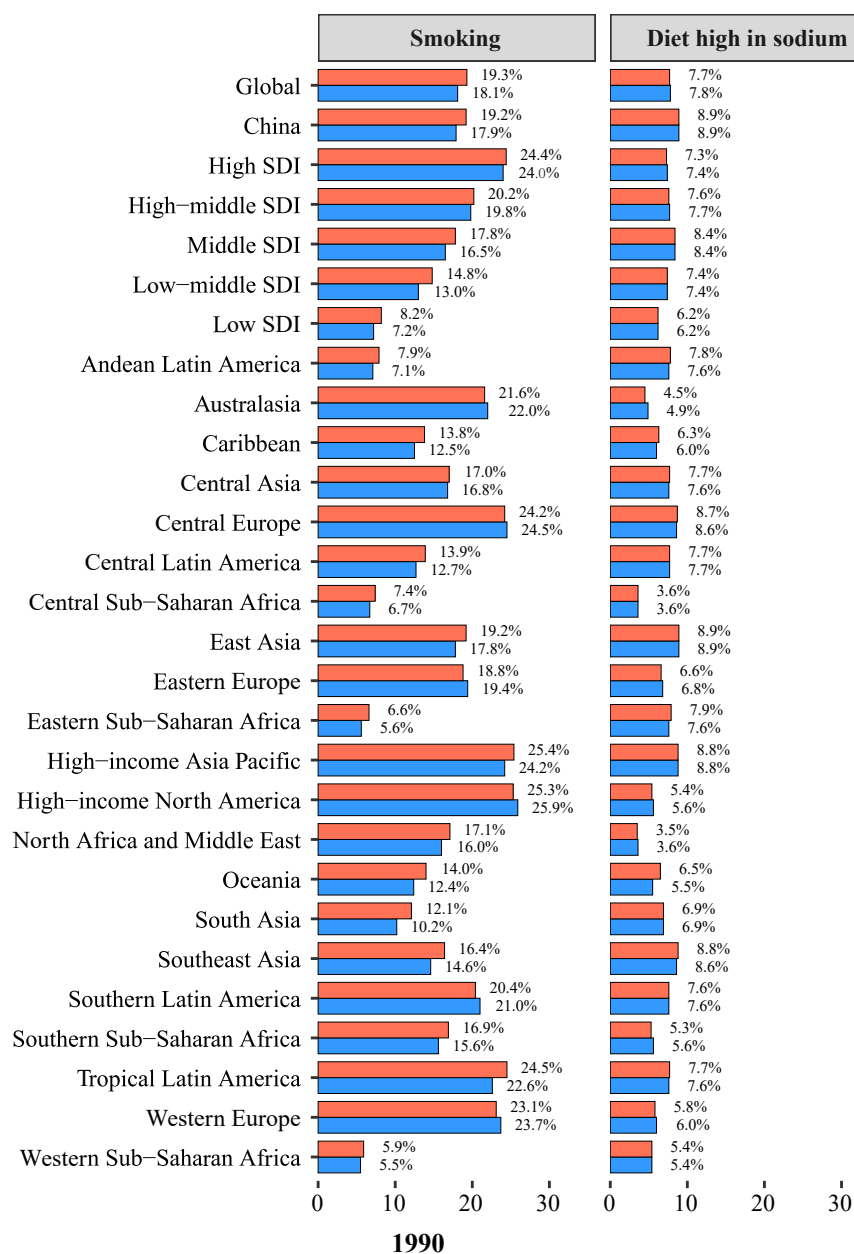

Deaths

B

Location

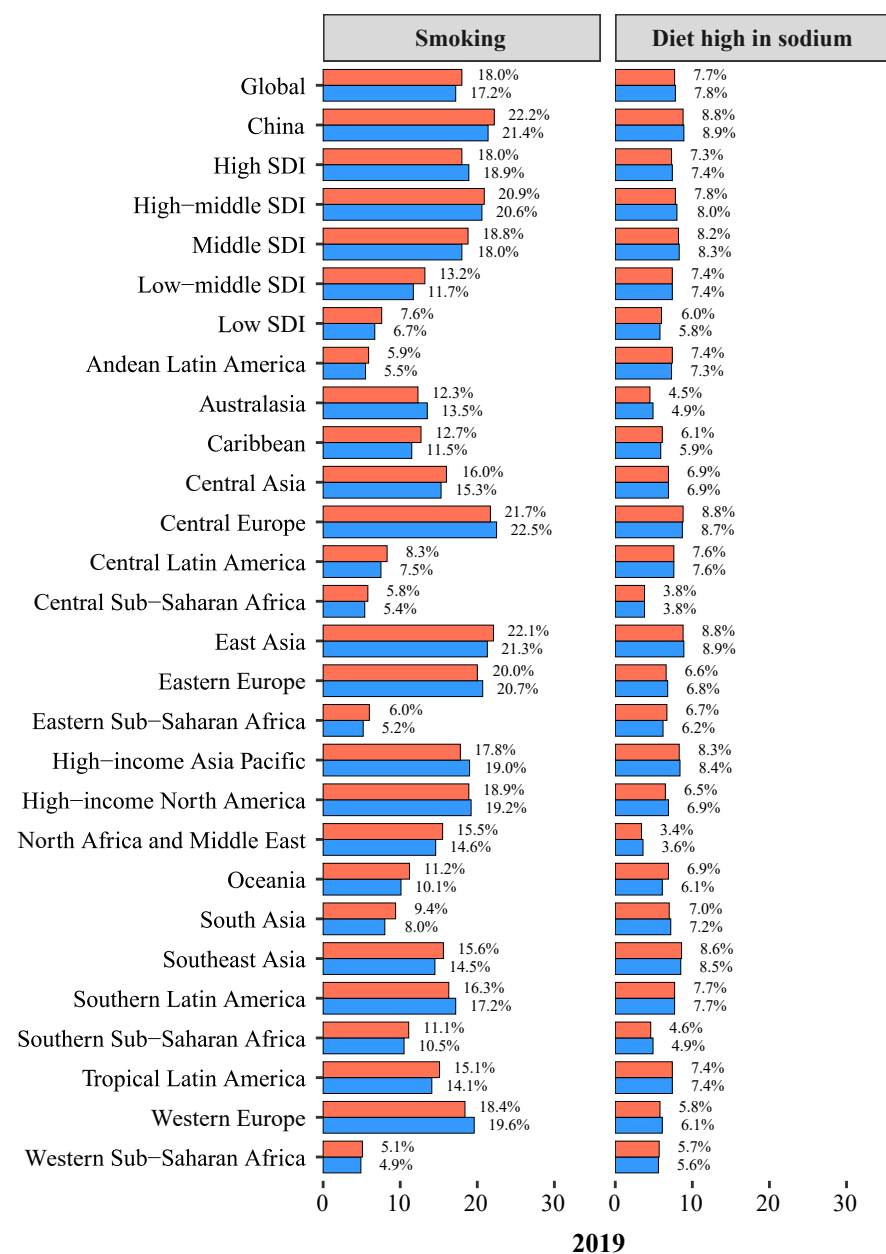

DALYs

Supplement: Supplementary file 1 [file Data_Sheet_1.ZIP › Supplementary Material/Fig. s6.pdf]

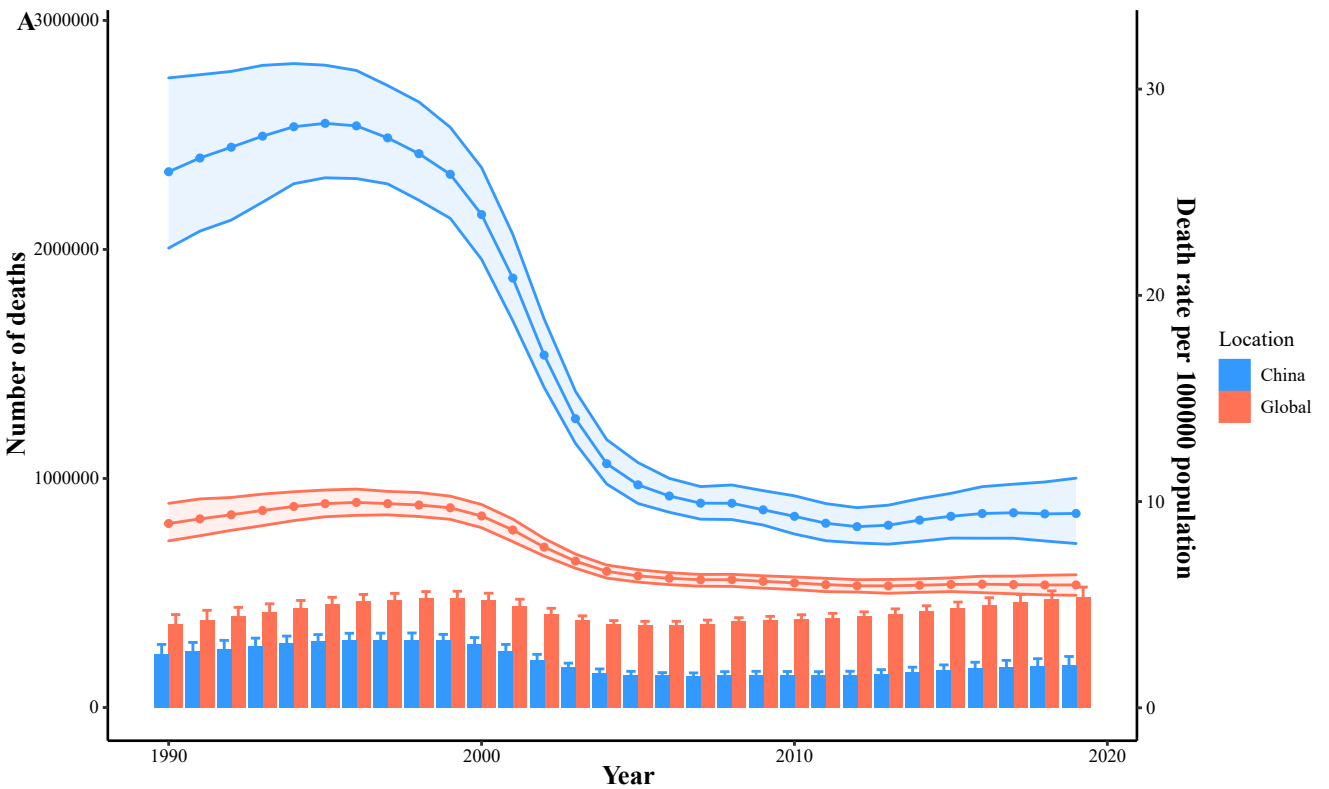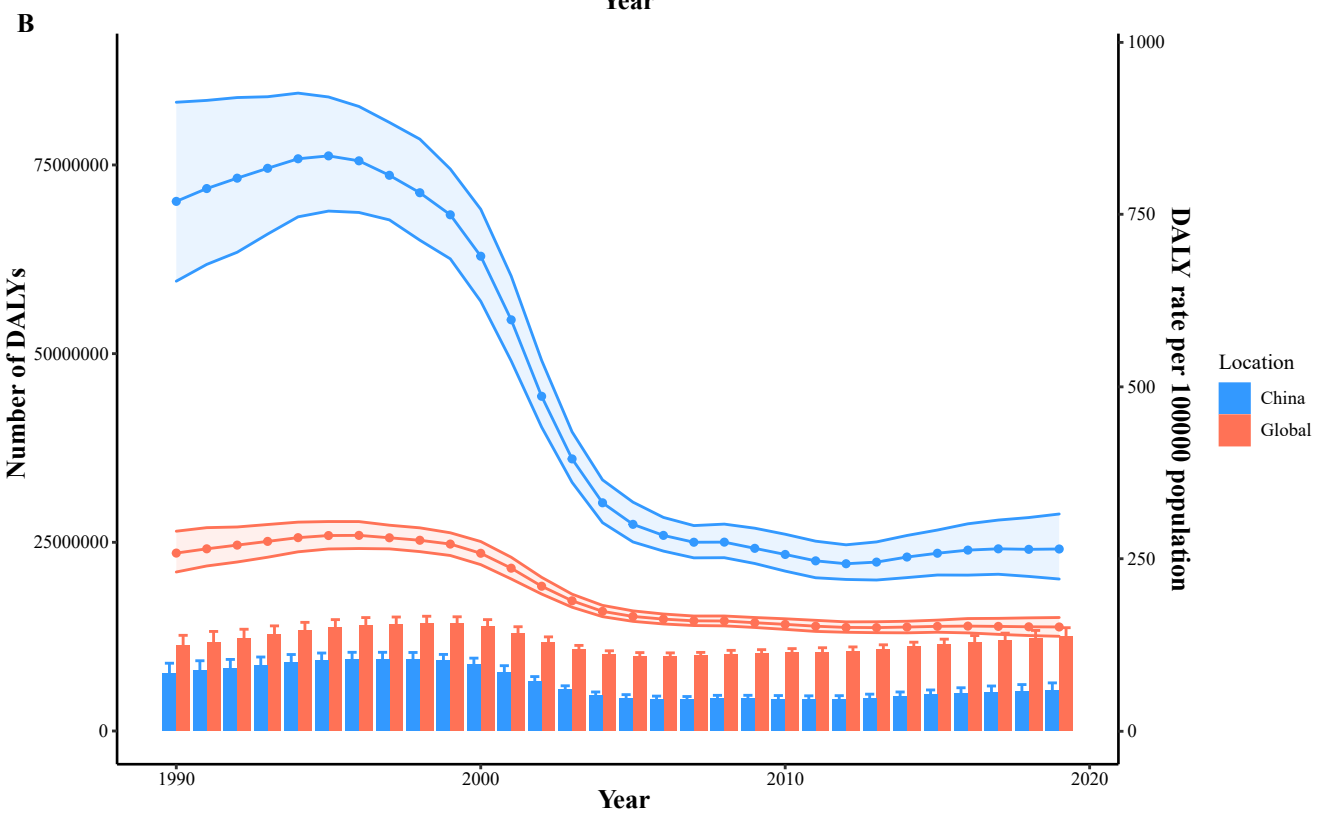

Supplement: Supplementary file 1 [file Data_Sheet_1.ZIP › Supplementary Material/Fig. s7.pdf]

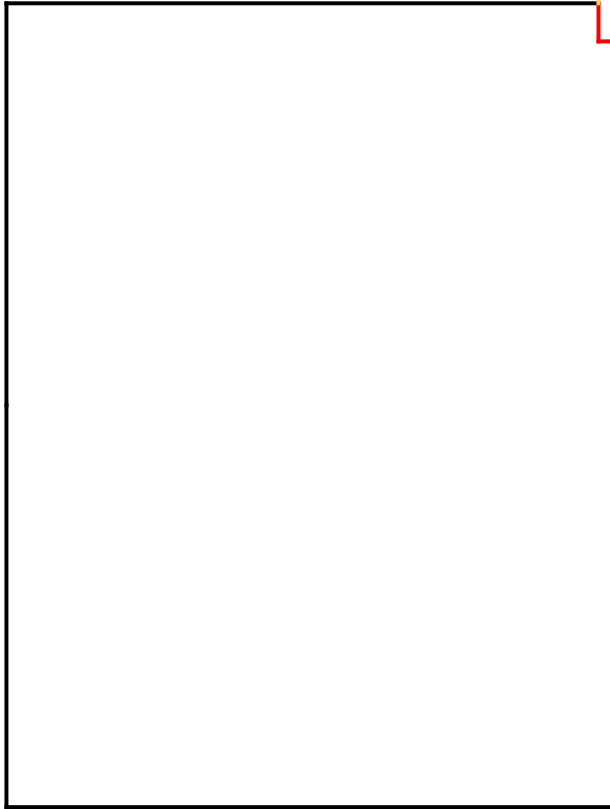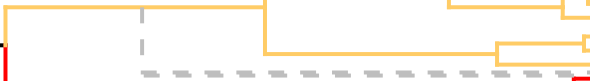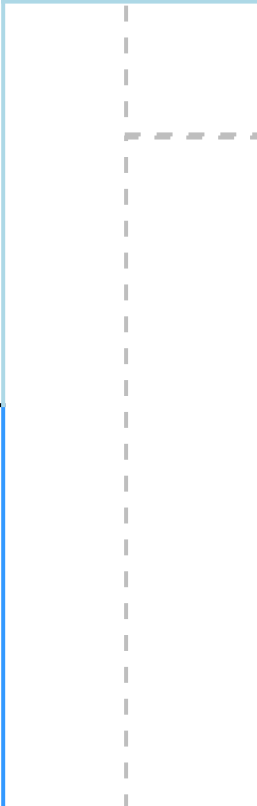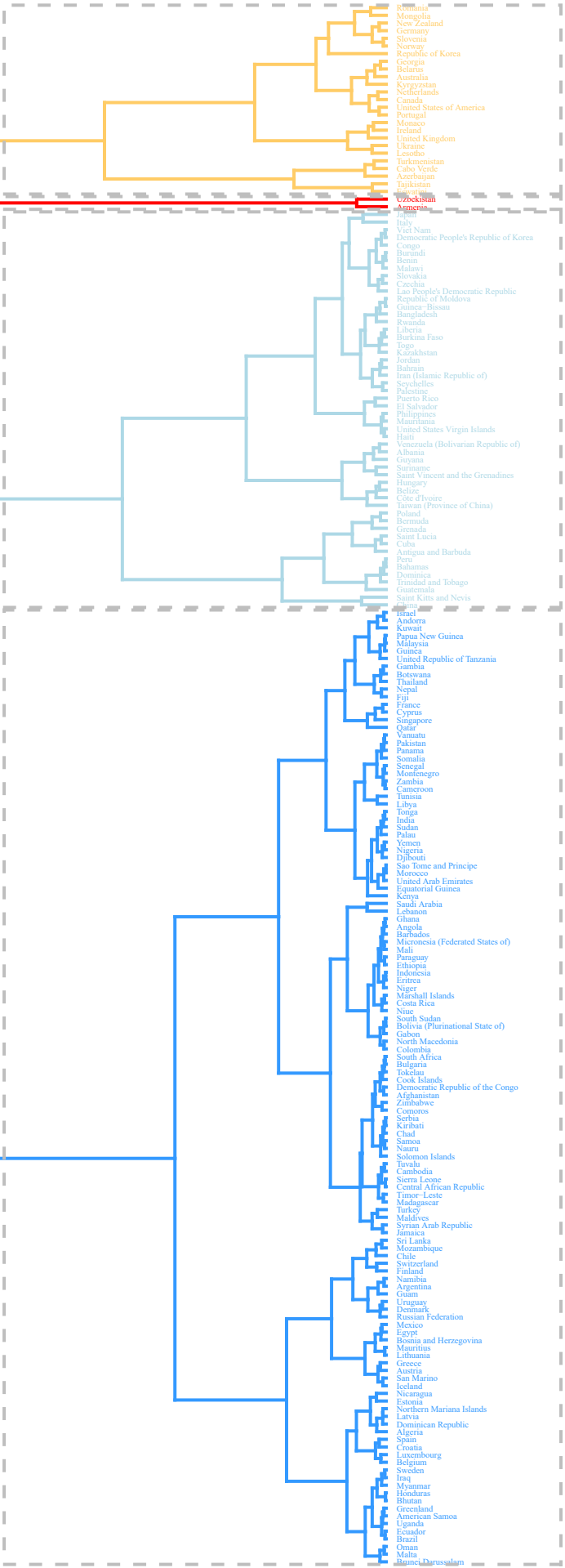

Supplement: Supplementary file 1 [file Data_Sheet_1.ZIP › Supplementary Material/Fig. s8.pdf]

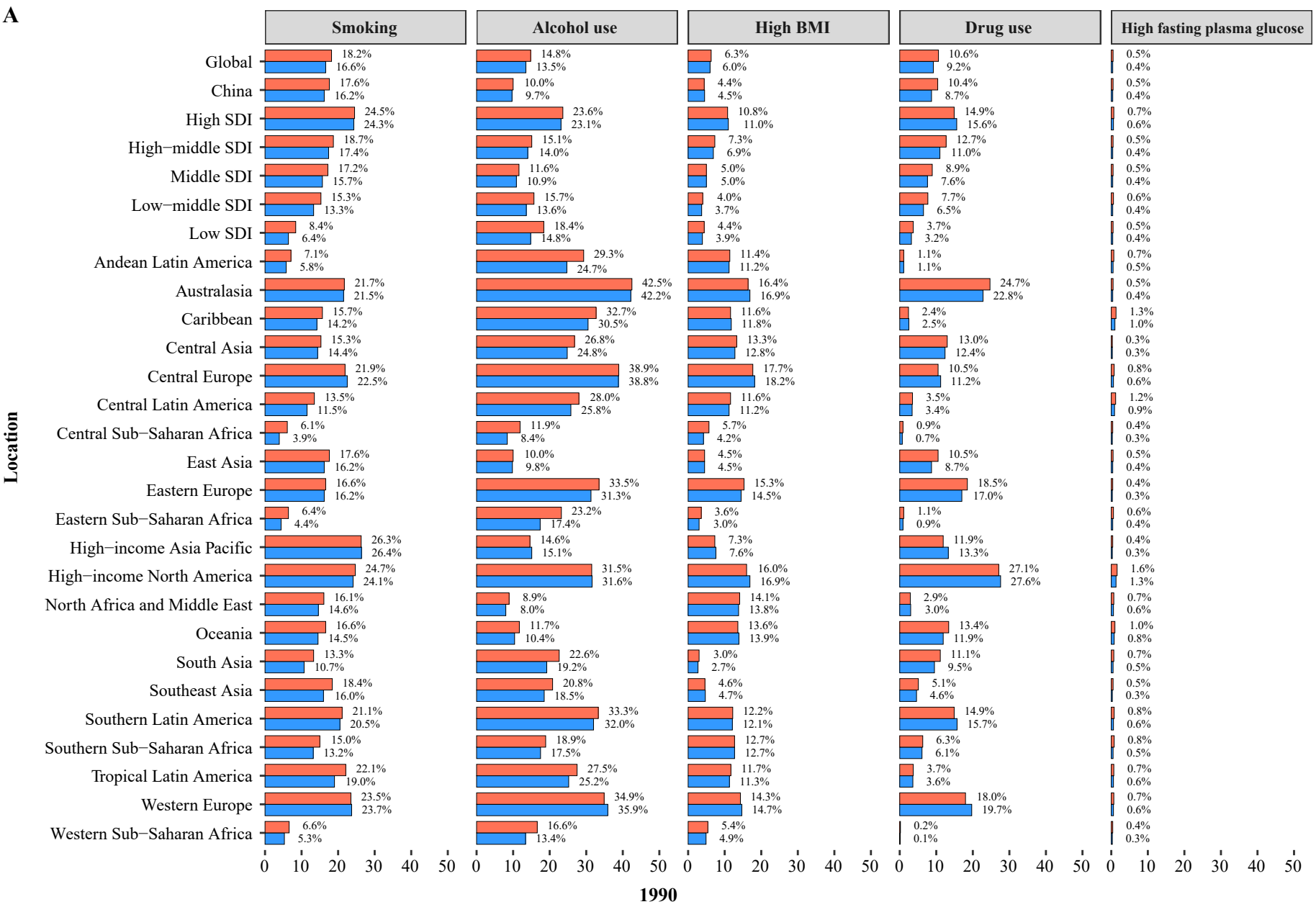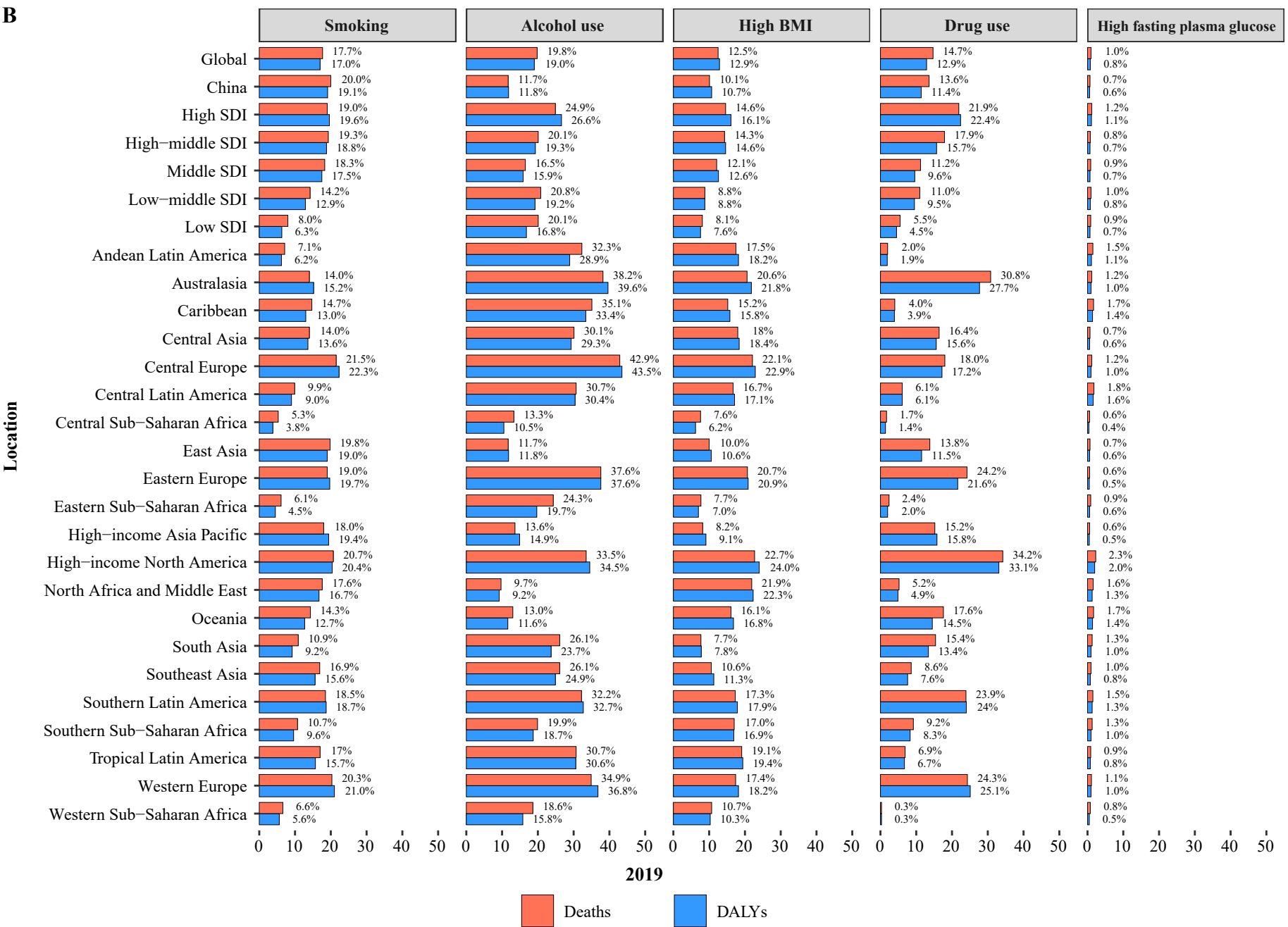

Supplement: Supplementary file 1 [file Data_Sheet_1.ZIP › Supplementary Material/Fig. s9.pdf]

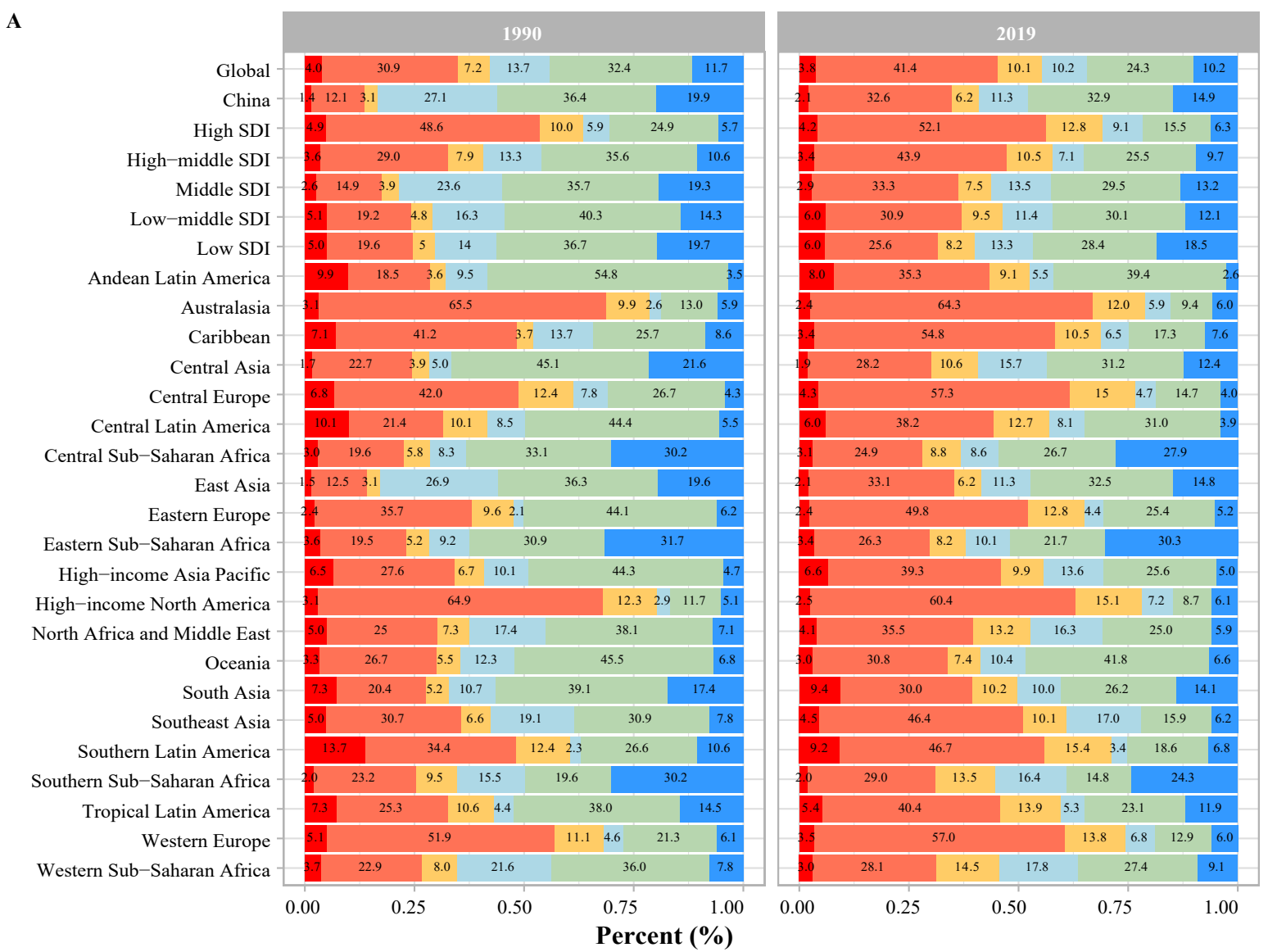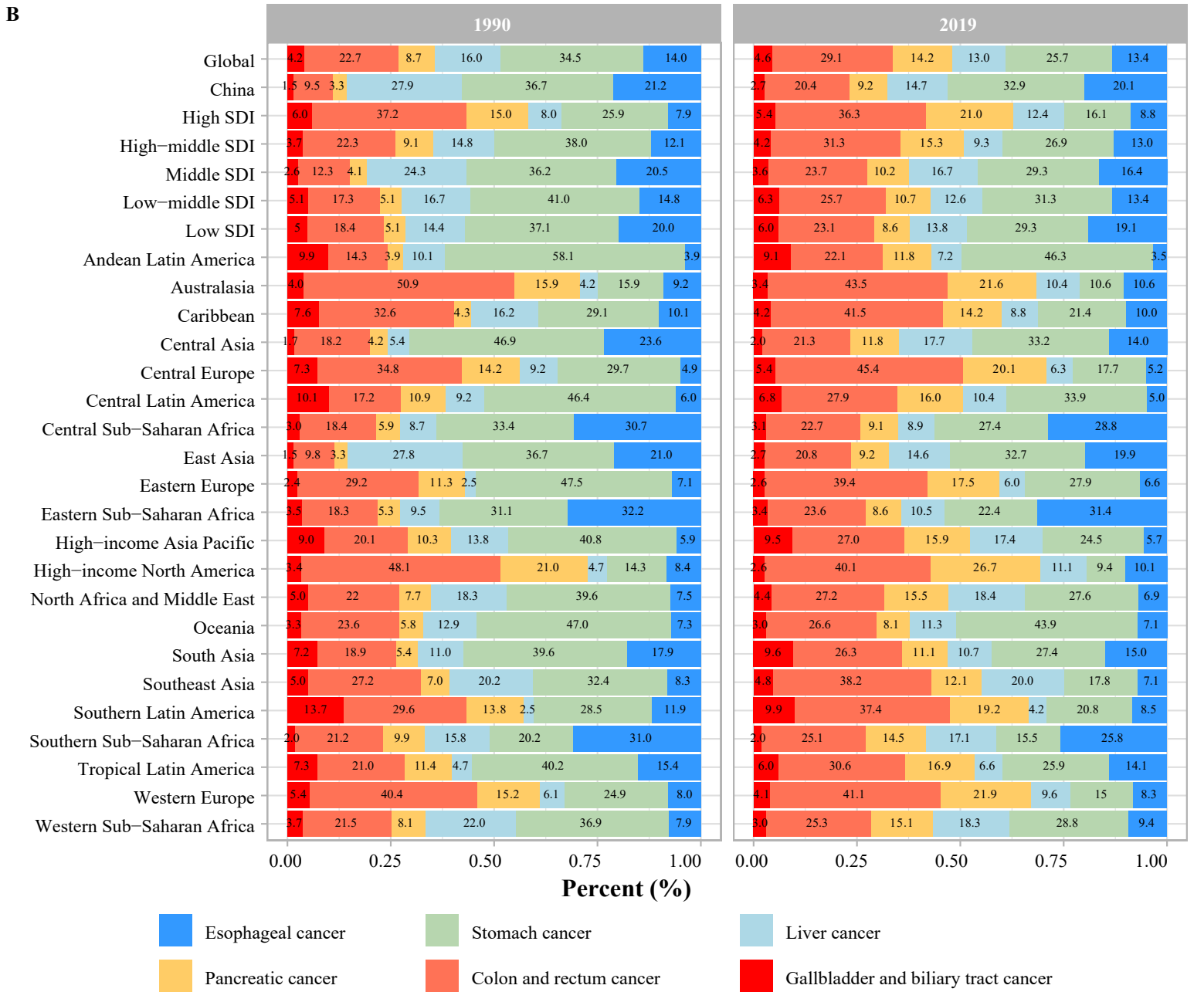

Supplement: Supplementary file 2 [file Image_1.pdf]
