# Supplementary material for: Chinese and Global Burdens of Gastrointestinal Cancers From 1990 to 2019
Source: Front Public Health. 2022 Jul 13;10:941284. doi: 10.3389/fpubh.2022.941284 (PMC9326121; doi:10.3389/fpubh.2022.941284)
Supplement: Supplementary file 1 [file Fig. s3.pdf › Supplementary Material/Fig. s3.pdf]

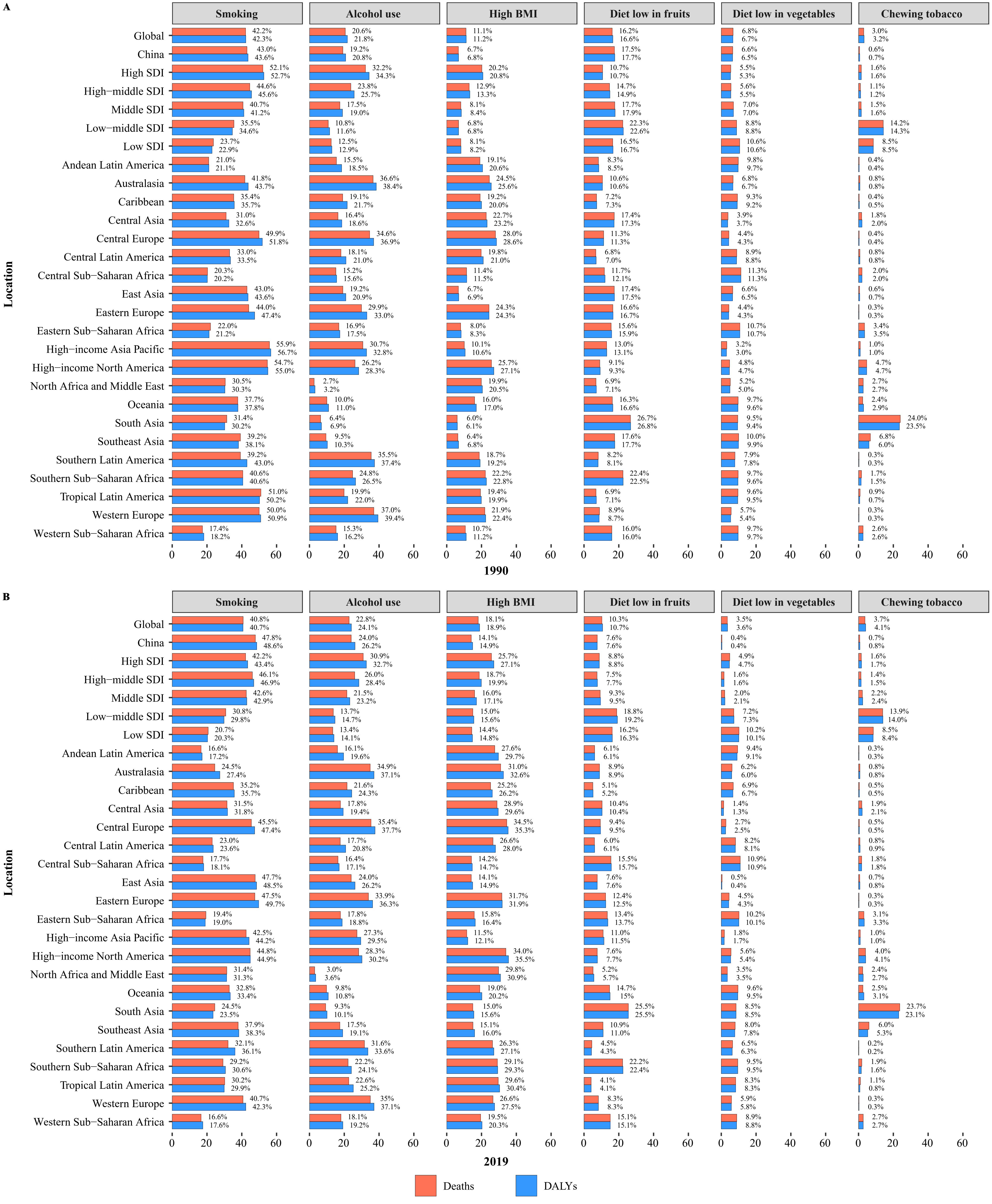
A
Smoking
Alcohol use
High BMI
Diet low in fruits
Diet low in vegetables
Chewing tobacco
Location
Global
China
High SDI
High−middle SDI
Middle SDI
Low−middle SDI
Low SDI
Andean Latin America
Australasia
Caribbean
Central Asia
Central Europe
Central Latin America
Central Sub−Saharan Africa
East Asia
Eastern Europe
Eastern Sub−Saharan Africa
High−income Asia Pacific
High−income North America
North Africa and Middle East
Oceania
South Asia
Southeast Asia
Southern Latin America
Southern Sub−Saharan Africa
Tropical Latin America
Western Europe
Western Sub−Saharan Africa
0 20 40 60
1990
B
2019
Deaths
DALYs
